# Developmental cues from epicardial cells simultaneously promote cardiomyocyte proliferation and electrochemical maturation

**DOI:** 10.1016/j.stemcr.2025.102572

**Published:** 2025-07-03

**Authors:** Sophie E. Givens, Abygail A. Andebrhan, Ruchen Wang, Xiangzhen Kong, Taylor M. Rothermel, Sanaz Hosseini, An Xie, Mohammad Shameem, Andrea A. Torniainen, Somayeh Ebrahimi-Barough, Samuel F. Boland, Maya Johnson, Natalia Calixto Mancipe, Bhairab N. Singh, Samuel Dudley, Patrick W. Alford, Elena G. Tolkacheva, Jop H. van Berlo, Brenda M. Ogle

**Affiliations:** 1 Biomedical Engineering, University of Minnesota, Minneapolis, MN, USA; 2 Lillehei Heart Institute (LHI), Department of Medicine, University of Minnesota, Minneapolis, MN, USA; 3 Electrical Engineering, University of Minnesota, Minneapolis, MN, USA; 4 Stem Cell Institute, University of Minnesota, Minneapolis, MN, USA; 5 Department of Pediatrics, University of Minnesota, Minneapolis, MN, USA; 6 Institute of Engineering in Medicine, University of Minnesota, Minneapolis, MN, USA; 7 Department of Rehabilitation Medicine, University of Minnesota, Minneapolis, MN, USA; 8 Minnesota Supercomputing Institute, University of Minnesota, Minneapolis, MN, USA

**Keywords:** epicardial cells, human induced pluripotent stem cells, cardiac maturation, calcium handling, cardiomyocyte proliferation, engineered heart tissues

## Abstract

Accumulating evidence indicates that maturation limits cardiomyocyte proliferation. We expand on that theory by co-culturing human induced pluripotent stem cell (hiPSC)-cardiomyocytes (CM) with epicardial cells (EPCs) and epicardial-derived cells in both 2D co-cultures and 3D engineered heart tissues (EHTs). In 2D co-cultures, the percentage of proliferating CM increased in parallel with stark electrophysiologic improvements. Single-cell transcriptomics revealed a significant shift in the bulk CM population of the epicardial-CM co-cultures as characterized by more fetal-like myofilament isoforms but with enhanced pathways associated with electrochemical maturation. The 3D-EHTs containing EPCs showed more limited proliferation but a similar improvement in CM electrophysiologic function. Next, epicardial-derived fibroblasts (EPD-FBs) were added to the EHTs containing EPCs, and we observed significant myofilament maturation and increased force generation. Our results suggest that some aspects of CM maturation (i.e., electrochemical) can occur when proliferation rates are relatively high, and that sarcomere-associated mechanical maturation occurs at later developmental stages when proliferation has largely ceased.

## Highlights

• hiPSC epicardial cells simultaneously enhance cardiomyocyte proliferation and maturation
• Single-cell transcriptomics revealed a distinct shift in the epicardial co-culture CM
• Epicardial cells do not enhance cardiomyocyte mechanical function
• Epicardial cells and fibroblasts in EHTs results in electrical and mechanical maturation

## Graphical abstract

**Figure:**
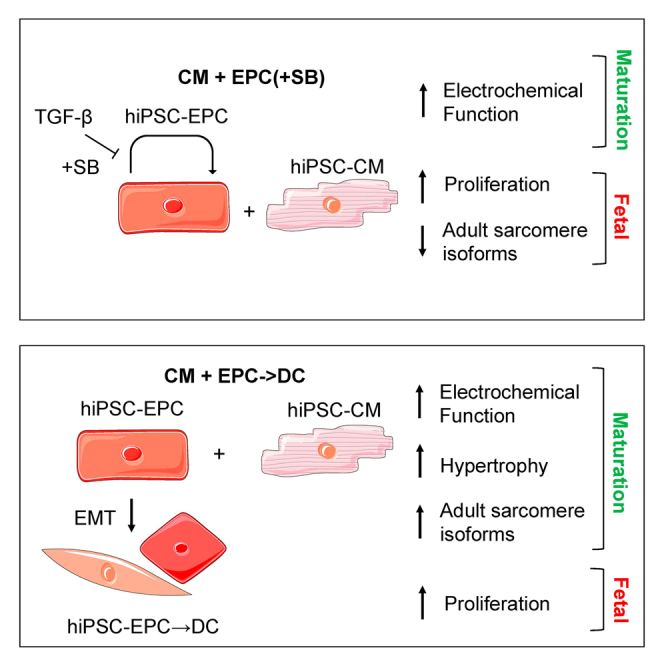

## Teaser

Here we show results that expand on the theory that maturation limits cardiomyocyte proliferation. We show that when hiPSC-cardiomyocytes are co-cultured with epicardial cells and epicardial-derived cells, we see increases in the percentage of proliferating CM at the same time as stark electrophysiologic improvements in calcium handling. This determines that, to some extent, electrochemical maturation can occur while proliferation is high.

## Introduction

The use of human induced pluripotent stem cell (hiPSC)-derived cardiac cells to study health and disease has exploded in the last decade with the advent of robust and reproducible methods for the differentiation of CM (Lian et al., 2012). Unfortunately, CM generated from such protocols are largely immature (Guo and Pu, 2020). This is evident by their small and rounded morphology, sarcomere disorganization, automaticity, the primary use of glycolysis for adenosine triphosphate (ATP) production, the expression of fetal myofibril isoforms, a fetal ion channel composition and lack of expression of major calcium handling proteins of the sarcoplasmic reticulum and T-tubule (Guo and Pu, 2020). Copious efforts to drive hiPSC-CM maturation *in vitro* have been attempted over the last decade with various levels of success. Approaches to drive maturation include the use of 3D culture systems that more accurately mimic the complex milieu of signals CM received *in vivo* such as organoids, engineered heart tissues (EHTs), and 3D printed chambered structures (Kupfer et al., 2020; Lemoine et al., 2017; Mills et al., 2017). The switch from 2D to 3D culture alone promotes some level of CM maturation (Branco et al., 2019; Correia et al., 2018; Ergir et al., 2022). Further, in EHTs, the imposition of increased mechanical “afterload” enhances force generation and maturation (Leonard et al., 2018). Additionally, some more user-friendly techniques exist including the use of “maturation media” to impose a switch from glucose to fatty acid-based metabolism and treatment with small molecule mitogen-activated protein kinase (MAPK) inhibitors that mimic the downregulation of the MAPK pathway seen in human ventricular tissues during development (Garay et al., 2022; Horikoshi et al., 2019). Finally, the co-culture of CM with other cell types present in the developing heart such as cardiac fibroblasts, endothelial cells, and epicardial-derived cells can enhance hiPSC-CM maturation (Bargehr et al., 2019; Beauchamp et al., 2020; Dunn et al., 2019; Giacomelli et al., 2020).

Most of these methods impose changes characteristic of post-natal heart development. The stark changes in afterload and preload caused by birth that result in developmental cardiac hypertrophy, the electrical stimulation present from the development of the cardiac pacemaker system, the switch to fatty acid metabolism, and the inhibition of pathways such as MAPK do not occur until postnatal development (Marchianò et al., 2019; Salameh et al., 2023). Freshly differentiated hiPSC-CM resemble early fetal CM that are largely undefined in patterning that takes place during embryonic development; during embryonic development, atrial, ventricular, compact, and trabecular myocardium show distinct, spatial, genetic, and functional patterning (Lai et al., 2010; Tian et al., 2017; Vicente-Steijn et al., 2015). Therefore, it might be advantageous to first mimic embryonic development before the implementation of post-natal conditions. This study explores the role of epicardial and epicardial-derived cells on hiPSC-CM and their ability to promote hiPSC-CM maturation by mimicking embryonic heart development.

The epicardium originates during fetal heart development in the proepicardial organ by E9.5 and migrates to cover the primary heart tube by E10.5-E12 in mice (Rudat and Kispert, 2012; Vicente-Steijn et al., 2015). Epicardial cells (EPCs) serve three major functions during embryonic development: (1) secretion of key growth factors that drive CM proliferation and formation of the compact myocardium (T. H.-P. Chen et al., 2002; Li et al., 2011); (2) the main source of interstitial fibroblasts in the myocardium (Acharya et al., 2012); and (3) the production of vascular smooth muscle cells that are responsible for driving coronary vascular formation (Grieskamp et al., 2011). The epicardium is so important for proper cardiac development that murine models with deficiencies in the epicardium have thin ventricular walls, impaired coronary artery angiogenesis, and embryonic or immediate perinatal lethality (Wu et al., 2013; Zamora et al., 2007). These findings indicate that the epicardium is essential for proper ventricular morphogenesis. Thus, this study explores the impact of epicardial and epicardial-derived cells on hiPSC-CM maturation by mimicking *embryonic* heart development.

Recently, protocols to differentiate and maintain EPCs using small molecule transforming growth factor β (TGF-β) inhibitors have been described (Bao et al., 2017). So far, only a few studies have looked at the effect of hiPSC-derived epicardial and epicardial-derived cells on hiPSC-CM phenotype and maturation. The first study showed that epicardial to epicardial-derived cells (EPC->DC)-containing EHTs exhibited increased force generation, enhanced electrophysiologic properties, and more mature CM phenotype, as seen by increased sarcomere length.^24^ In another study, EPCs were co-cultured with cardiac progenitor cells with and without TGF-β inhibition (Floy et al., 2022). This study reported an increase in CM proliferation and an associated increase in sarcomere disarray expected from proliferating CM. However, the co-culture of EPCs with cardiac progenitors does not fully recapitulate embryonic development as the primary heart tube is already spontaneously contracting by E8.0 indicating they have the contractile machinery to be defined as CM (Tyser and Srinivas, 2020). Thus, a study determining the effect of epicardial and epicardial-derived cells on differentiated CM might more accurately recapitulate development. More recently, proepicardial cells have been combined with ventricular CM in aggregates, and these showed increased calcium handling and CM sarcomere length as well as IGF2-induced CM proliferation, indicating a role of EPCs in both CM maturation and proliferation (Tan et al., 2021). The tissues were cultured in a media formulated to reduce epicardial epithelial-to-mesenchymal transition (EMT) but there were still ∼8% uncharacterized cells in their culture indicating potential epicardial-EMT that TGF-β inhibitors might have ameliorated (Tan et al., 2021). The CM functional assessment in this study was limited to calcium and contractility and maturation markers were evaluated using bulk qPCR versus more powerful techniques such as single-cell RNA sequencing (scRNA-seq) necessary to determine cell-specific effects of the EPC on the ventricular CM (Tan et al., 2021).

The building literature and new technical advances provided a scientific premise to evaluate the hypothesis that embryonic-to-fetal multicellular interactions drive CM maturation in ways distinct from stimuli of the adult heart. Here we mimic fetal development by creating co-cultures of CM with fetal-like hiPSC-derived EPCs and find that they simultaneously enhance hiPSC-CM electrochemical function, proliferation, and fetal sarcomeric expression profiles. When fibroblasts, cells essential for post-natal heart maturation, are added to this multicellular experimental framework, proliferation is lost, electrochemical maturation is sustained, and sarcomere-mediated mechanical maturation is added. Thus, this work defines an intermediate condition on the road to CM maturation wherein electrochemical maturation, but not mechanical maturation can occur with CM proliferation.

## Results

### Mouse embryonic CM maturation and proliferation at E10, E12, and E17

The degree of CM maturation that occurs during embryonic development is understudied. To explore this and the potential role of the epicardium in embryonic CM maturation, three embryonic time points were identified that correspond to well-known dynamics of the formation of the epicardium. E10 was chosen as it corresponds to a time point where the primary heart tube is spontaneously contracting and the proepicardial organ is fully formed but few EPCs have covered the heart tube (Rudat and Kispert, 2012; Vicente-Steijn et al., 2015). E12 corresponds to a time point where EPCs have fully encapsulated the heart to form the epicardium but substantial amounts of EMT have not occurred (Acharya et al., 2012; Vicente-Steijn et al., 2015). E17 corresponds to a time point where substantial amounts of EMT have occurred and stromal cells are present throughout the myocardium (Acharya et al., 2012).

**Figure 1:**
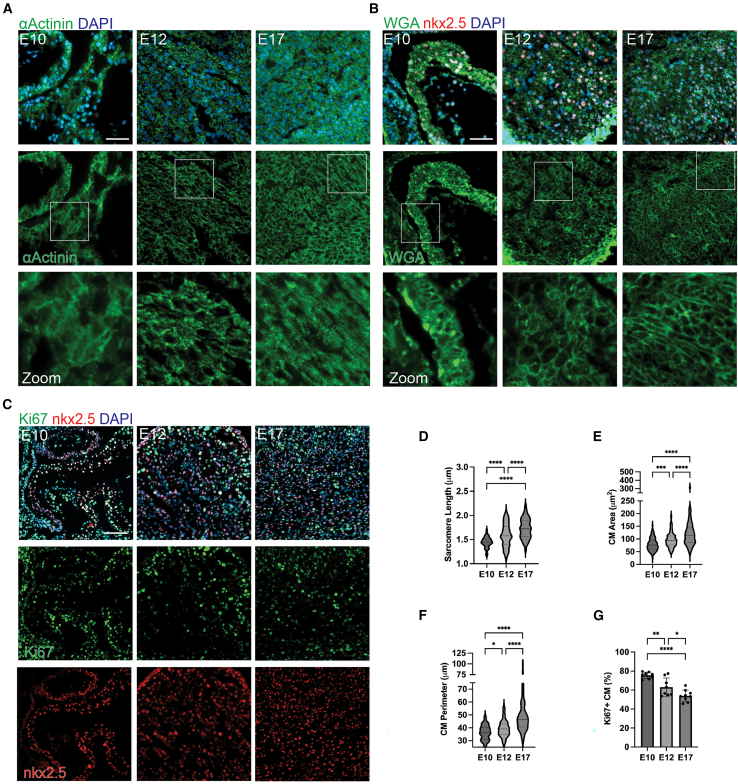
Mouse cardiomyocyte maturation during embryonic development is substantial (A) Representative images from E10 (left), E12 (middle), and E17 (right) mouse hearts stained for α-actinin (green) for CM sarcomere visualization and DAPI (blue). (B) Representative images of wheat germ glutenin (WGA; green) for cell membrane visualization, nkx2.5 (red) marking first heart field cardiomyocytes, and DAPI for nuclei visualization (blue) from E10 (left), E12 (middle), and E17 (right) mouse heart sections. (C) Representative images of mouse heart sections stained for nkx2.5 (red) marking first heart field cardiomyocytes, Ki67 (green) marking proliferation, and DAPI (blue) marking the nucleus from E10 (left), E12 (middle), and E17 (right). The quantified average percent proliferating CM as determined by the co-localization for Ki67 with nkx2.5 and DAPI. Scale bar, 100 μm. (D–G) (D) Quantified CM sarcomere length, (E) CM area, (F) CM perimeter, and (G) the percentage of proliferating CM at each developmental time point. For (D–F), the violin plot center dashed line represents the median and outer dashed lines represent upper and lower quartiles of the distribution. For (G), the bar graph and error bars represent the mean ± SD and each dot represents the average percent Ki67 and nxk2.5 co-positive nuclei from multiple sections across E10 (*n* = 8), E12 (*n* = 8), and E17 (*n* = 9) embryonic mouse hearts. For (D), E10 (*n* = 87), E12 (*n* = 101), and E17 (*n* = 108) sarcomeres across E10 (*n* = 8), E12 (*n* = 8), and E17 (*n* = 9) embryonic mouse hearts were measured. For (E and F), E10 (*n* = 116), E12 (*n* = 122), and E17 (*n* = 128) were measured. ^∗^*p* < 0.05, ^∗∗^*p* < 0.01, ^∗∗∗^*p* < 0.001, and ^∗∗∗∗^*p* < 0.0001.

During embryonic development, CM remains proliferative but almost entirely exits the cell cycle in the first week after birth (Bergmann et al., 2015). Thus, decreases in proliferation are a sign of advancing maturation in CM. CM proliferation was examined in parallel with common hiPSC-CM maturation parameters to see if the maturation indices (1) happened during embryonic development and (2) showed the same proliferation-maturation dichotomy as has been demonstrated in postnatal development (Singh et al., 2023). Sarcomere length was assessed using staining for α-actinin (Figure 1A). CM area and perimeter was assessed via wheat-germ agglutinin (WGA) for membrane visualization, nkx2.5 to mark CM, and DAPI staining (Figure 1B). Lastly, the percent of proliferating CM were visualized and quantified using the co-localization nkx2.5 with Ki67 and DAPI (Figure 1C). The sarcomere length increased substantially over these developmental time points from 1.44 ± 0.11 μm at E10 to 1.59 ± 0.23 μm at E12 and 1.73 ± 0.20 μm at E17 (Figure 1D). CM area and perimeter showed increases as well (Figures 1E and 1F). CM proliferation at E10 was the greatest (75% ± 3%) and decreased by ∼10% at E12 (63 ± 10%) and another 10% at E17 (54% ± 6%) (Figure 1G). These findings confirm that even at an early time point, when CM proliferation is high, CM proliferation is inversely related to maturation *in vivo*. They also confirm that substantial amounts of CM maturation occur during embryonic development; thus, hiPSC-CM can potentially be driven to mature by mimicking embryonic interactions with EPCs.

### Epicardial co-culture composition in 2D

**Figure 2:**
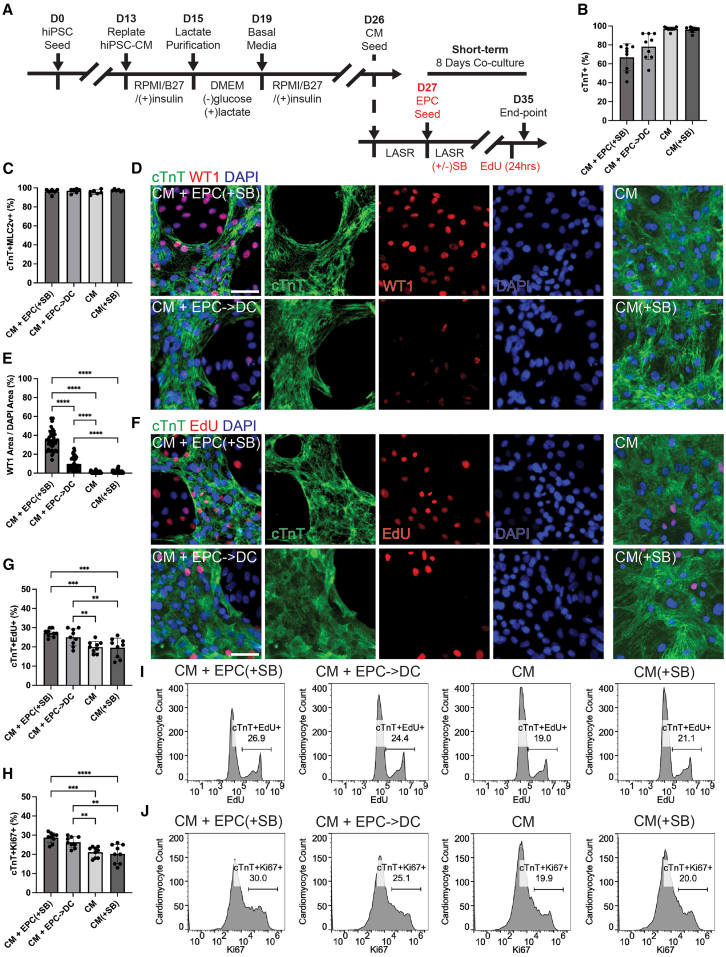
Epicardial cells increase CM proliferation in 2D co-cultures (A) 2D co-culture experimental timeline where red text indicates experimental variables. Where either epicardial cells (EPCs) were seeded on day 27 or not, and each condition with or without EPCs was maintained in LASR media with or without TGF-β inhibitors (+/−SB). Cells fated for flow cytometry were incubated in EdU for 24 h before fixation while the others were assessed functionally. (B) Flow cytometry for cardiac troponin T (cTnT) indicating the percentage of CM in each experimental condition. (C) Flow cytometry for cTnT and myosin light chain 2 (MLC2v) indicating the percentage of ventricular CM in each condition. (D) Representative IHC images from M4-hiPSC 2D co-cultures at day 35 stained for cTnT (green), WT1 (red—epicardial cells), and the nucleus (DAPI—blue). (E) Image quantification of WT1 area/DAPI area to determine the percentage of epicardial cells in all four conditions. (F) Representative images of cTnT (green) and proliferating cells after 24-h EdU incubation (red). (G) Flow cytometry quantification of cTnT^+^EdU^+^ and (H) cTnT^+^Ki67^+^ percentage for all experimental replicates. (I and J) Representative M4 flow cytometry histograms of co-cultures with visualization of the cTnT+ population (CM count) with (I) EdU or (J) Ki67 on the *x* axis. Scale bars, 50 μm. Bar graphs represent the mean ± SD. For (B, E, G, and H), *n* = 3 independent experiments for each hiPSC-line (M4, F7, and CCND2) and (C) *n* = 3 independent experiments for two of the hiPSC-lines (M4 and F7). ^∗^*p* < 0.05, ^∗∗^*p* < 0.01, ^∗∗∗^*p* < 0.001, and ^∗∗∗∗^*p* < 0.0001 for (C, E, G, and H). See also Figure S1.

The effect of EPCs on hiPSC-CM maturation and function was first explored in 2D. EPCs were seeded on top of CM and the co-cultures were either maintained in TGF-β inhibitor (SB431542) to prevent EMT (CM + EPC[+SB]) or in basal epicardial media to allow spontaneous EMT into epicardial derived cells (CM + EPC->DC) to occur (Figure 2A). The CM + EPC->DC condition allowed us to study a situation in which the EPC population is gradually being diluted by the emergence of stromal cells, as in development. Extended exposure to ascorbic acid-containing media was used to drive a ventricular phenotype of the hiPSC-CM in all conditions to limit variation between other CM subtypes (Kim et al., 2023). Due to the expansion of the epicardial population in the presence of the TGF-β inhibitor, the CM + EPC(+SB) conditions contained 67% ± 14% cardiac troponin T (cTnT) positive CM and the CM + EPC->DC condition contained 78% ± 14% after 8 days of co-culture (Figure 2B). The CM population in the CM controls without (97% ± 2%) and with (96% ± 3%) TGF-β inhibition remained high (Figure 2B). Epicardial co-culture did not increase the percentage of ventricular CM in culture since all conditions contained ≥97% myosin light chain 2v positive (MLC2v) CM (Figure 2C).

Immunocytochemistry (ICC) for cTnT, Wilms tumor 1 (WT1), an epicardial marker, and DAPI were used to assess the percentage of EPCs remaining after 8 days of co-culture (Figure 2D). There was substantial migration of EPCs into the CM layer depicted by red nuclei interspersed within the cTnT positive area (Figure 2D). Additionally, large patches of highly confluent epithelial cells can be seen separate from the CM in bright field images (Figure S1A). Quantification of the WT1 cell population determined that the CM + EPC(+SB) condition contained 35% ± 11% WT1 positive cells while a significant portion of the EPCs in the EPC->DC conditions underwent EMT and only 10% ± 7% WT1 positive cells remained (Figure 2E). Three hiPSC-lines were used for these experiments and the maintenance of WT1 positive cells as well as the cTnT positive population is consistent between all three lines in each condition (Figures S1B–S1D). The MLC2v^+^ CM population was consistent across the two lines tested (Figures S1E–S1G). These data serve to validate the composition of cells present in the 2D *in vitro* model.

### Epicardial cells enhance CM proliferation in 2D

Since multiple animal studies show that EPCs are essential for CM proliferation during development, we aimed to explore this using hiPSC-derived cells. The co-cultures and controls were given ethynyl deoxy-uridine (EdU) for the last 24 h in culture and stained for both cTnT and EdU or Ki67 a nuclear transcription factor that marks cell proliferation (Figures S1H and S1I). The percent proliferation, as indicated by the cTnT^+^EdU^+^ population, increased from ∼20%, in both CM and CM(+SB) conditions, to 27% ± 2% in the EPC(+SB) conditions and 25% ± 4% in the EPC->DC condition (Figures 2F and 2G). This increase in proliferation was consistent with the expression of Ki67 (Figure 2H). Histograms of the CM population versus EdU or Ki67 were used to determine the percentage of proliferative CM (Figures 2I and 2J). This confirms that hiPSC-EPCs, which are maintained or are currently undergoing EMT, both increase CM proliferation as in animal models during development. This finding was consistent for the CM + EPC(+SB) condition in all three hiPSC-lines (Figures S1J and S1K). It was also consistent for the CM + EPC->DC condition for the M4 and F7 lines but not the CCND2-hiPSC line. We noted that the co-cultures from the CCND2 line exhibited a lower percentage of EPCs in the CM + EPC->DC condition (Figure S1B). This could reflect a more robust EMT of the CCND2-EPCs (Figure S1C). In summary, EPCs spur proliferation, but a large enough dose may be required to do so, and that dose might be dependent on the characteristics of the stem cell source line.

### Epicardial cells enhance select indices of CM functional maturation in 2D

**Figure 3:**
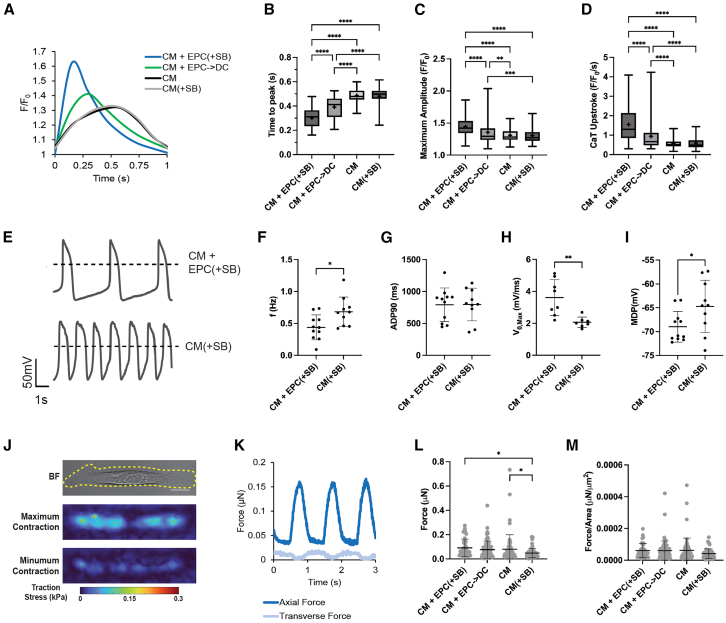
Epicardial cells enhance CM function in 2D co-cultures (A) Representative direct co-culture calcium transient (CaT) traces of CM from the M4-line for each condition. (B–D) Quantification of CaT parameters (B) time to peak, (C) maximum amplitude, and (D) CaT upstroke velocity. (E) Representative patch clamp trace for the CM + EPC(+SB) and CM(+SB) conditions. (F–I) Quantification of patch clamp parameters (F) beating frequency (f), (G) the AP duration at 90% repolarization (APD90), (H) the AP maximum upstroke velocity (V_0,max_), and (I) the maximum diastolic potential (MDP). (J) The maximum and minimum traction stress maps for a representative CM determined using traction force microscopy (TFM). (K) Axial and transverse dorce versus time plot for three contractions of a representative cell. Scale bars, 20 μm. (L and M) (L) CM contractile force determined by TFM and (M) CM contractile force normalized to ell area. (B–D) Box and whisker plots with + marking the mean. (B–D) Data compiled from *n* = 3 independent experiments from each hiPSC-lines (M4, F7, and CCND2). For (F–I) and (L and M), center lines and error bars represent the mean ± SD. For (F, G, and I), CM + EPC(+SB) (*n* = 11) and CM(+SB) (*n* = 10); for (H), CM + EPC(+SB) (*n* = 8) and CM(+SB) (*n* = 7) cells across *n* = 3 independent experiments from the M4-line; and for (L and M), CM + EPC(+SB) (*n* = 41), CM + EPC->DC (*n* = 74), CM (*n* = 60), and CM(+SB) (*n* = 54) across *n* = 3 independent experiments from each hiPSC-line. ^∗^*p* < 0.05, ^∗∗^*p* < 0.01, ^∗∗∗^*p* < 0.001, and ^∗∗∗∗^*p* < 0.0001. See also Figure S2.

To determine whether and to what extent functional maturation of CM in the epicardial and epicardial-derived cell co-cultures occurred, calcium transient (CaT) assessment was conducted at the experimental endpoint. From embryonic to post-natal and adult development, there are stark increases in the amount and rate of release and sequestration of calcium from the sarcoplasmic reticulum as calcium is the key mediator of excitation-contraction coupling (Liu et al., 2002). The CM co-cultures calcium transients were assessed using calcium sensitive dye under paced conditions (Figure 3A). Most notably, the presence of EPCs resulted in a stark decrease in CaT time to peak (Figure 3B). The robust decrease in time to peak for the epicardial co-culture conditions was consistent across all three hiPSC-lines (Figure S3). Overall, the CM + EPC(+SB) condition showed the most mature calcium handling dynamics followed by the CM + EPC->DC condition. Both conditions showed increased maximum amplitude and CaT upstroke velocity (Figures 3C and 3D). This increase was seen across all three hiPSC-lines in the CM + EPC(+SB) condition but was lacking in the CCND2 CM + EPC->DC condition most likely due to the lowest remaining amount of EPCs in this line (Figure S3). There was also a significant increase in downstroke velocity for the CM + EPC(+SB) condition on average (Figures S2A–S2C).

To further assess the electrophysiologic properties of CM in the CM + EPC(+SB) condition and its control, CM(+SB), the cells were replated, and patch clamp for CM action potential (AP) was performed (Figure 3E). The APs of ventricular hiPSC-CM have slower upstroke velocities during the rapid depolarization phase, lack of a “notch,” and shorter plateau phases due to immature ion channel compositions in comparison to adult CM (Goversen et al., 2018). The CM from the epicardial co-culture had a significant decrease in beat rate frequency (f) (Figure 3F). Both CM(+SB) and CM + EPC(+SB) have a long AP duration at 90% repolarization (APD90) of ∼800 ms, which is characteristic of ventricular CM (Figure 3G). Consistent with the CaT data, the maximum upstroke velocity was almost doubled in the CM + EPC(+SB) (Figure 3H). Additionally, a decrease in maximum diastolic potential (MDP) from −65 ± 6 to −69 ± 3 mV was seen in the epicardial co-cultures suggesting a trend toward the resting potential of mature adult ventricular CM of −85 mV (Guo and Pu, 2020). In summary, patch clamp data further support increased electrochemical maturation of CM in epicardial co-cultures.

Another functional attribute of maturation is increased force generation at the tissue and cellular levels (Wheelwright et al., 2018). To explore the cellular level mechanical function of the hiPSC-CM, the co-cultures were singularized and traction force microscopy (TFM) was performed (Figures 3J and 3K). There were no significant differences between the CM-only conditions and the EPC co-culture conditions. There was, however, a significant decrease in force in the CM(+SB) condition compared to the CM condition (Figure 3L). The negative effect of the TGF-β inhibitor were ameliorated by the positive effect of the EPCs (Figure 3L). However, when normalized to the CM area, there are no significant differences in CM stress generation (Figure 3M). The force generation data are broken down by line in the supplement (Figures S2D–S2F). These data indicate that the effect of EPCs on CM maturation is more substantial with regard to electrochemical parameters and does not appear to include mechanical maturation at a cellular level.

### Epicardial cells enhance CM morphologic maturation in 2D

**Figure 4:**
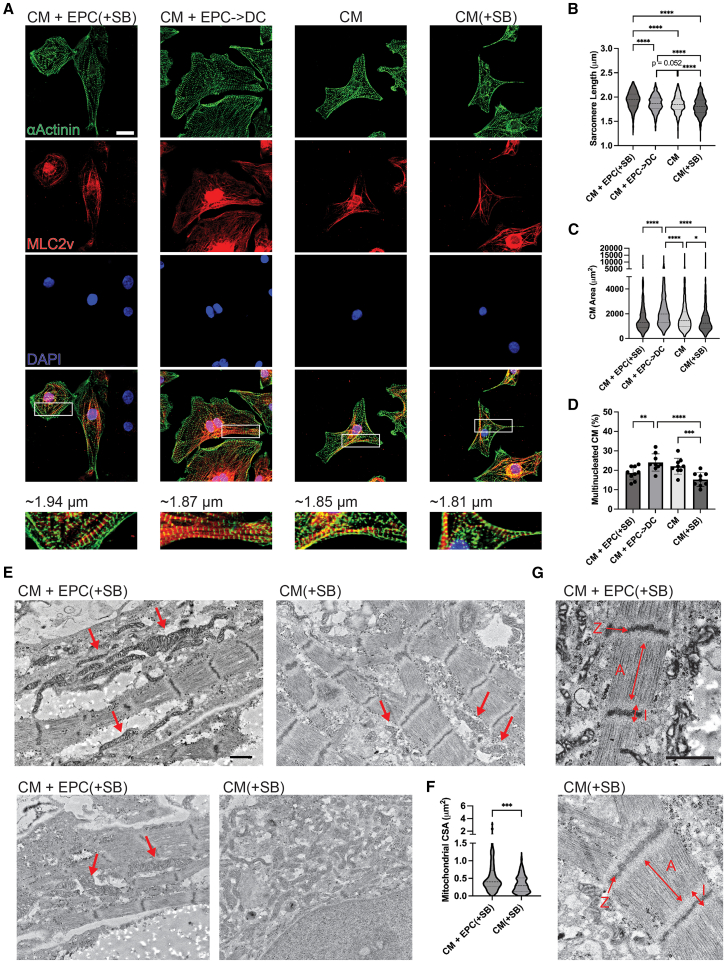
Epicardial cells modestly increase CM morphologic maturation (A) Representative IHC images of 2D co-cultures replated onto glass slides and stained with ⍺-actinin (green) for sarcomere visualization, MLC2v for ventricular phenotype validation (red), and DAPI (blue) for nuclear visualization. Scale bars, 20 μm. (B–D) Violin plots showing (B) quantified sarcomere length (C) CM area, and (D) the percent multinucleated CM. (E and F) (E) Representative TEM images with red arrows indicating mitochondria in close apposition to sarcomeres and (F) the quantified mitochondrial cross-sectional area (CSA). (G) TEM image of sarcomeres showing the Z-line (Z), I-band (I), and A-band (A) for each condition. Scale bars, 1 μm. In (B, C, and F), violin plots center dashed line represents the median, and outer dashed lines represent the upper and lower quartiles. In (B and C), the data represent CM across *n* = 3 independent experiments for all three hiPSC-lines (M4, F7, and CCND2); for (B), CM + EPC(+SB) (*n* = 784), CM + EPC->DC (*n* = 839), CM (*n* = 799), and CM(+SB) (*n* = 713); and for (C), CM + EPC(+SB) (*n* = 878), CM + EPC->DC (*n* = 878), CM (*n* = 873), and CM(+SB) (*n* = 817). For (D), the bar represents the mean ± SD for *n* = 3 independent experiments for all three hiPSC-lines (M4, F7, and CCND2) and each dot represents the averages of 5 fields of view across three wells for each independent experimental replicate. In (F), the CSA of CM + EPC(+SB) (*n* = 79) and CM(+SB) (*n* = 81) mitochondria across 10 cells per condition for *n* = 1 independent experiment from the M4-hiPSC line was measured. ^∗^*p* < 0.05, ^∗∗^*p* < 0.01, ^∗∗∗^*p* < 0.001, and ^∗∗∗∗^*p* < 0.0001. See also Figure S2.

We next evaluated the CM of the co-cultures for morphologic features of maturation. The sarcomeres were visualized via staining for α-actinin and the ventricular phenotype was validated with MLC2v staining (Figure 4A). The adult myocardium has an average sarcomere length of 2.25 μm (Sonnenblick et al., 1967). The longest sarcomere length was detected in the CM + EPC(+SB) co-cultures at 1.94 ± 0.17 μm followed by 1.87 ± 0.16 μm in the CM + EPC->DC condition, while the CM condition had an average length of 1.85 ± 0.17 μm (Figure 4B). Without the aid of EPCs, the TGF-β inhibition resulted in a significant decrease in CM(+SB) sarcomere length (1.81 ± 0.18 μm). The increase in CM sarcomere length with EPC co-culture was consistent across all three hiPSC-lines whereas TGF-β inhibition only negatively affected sarcomere length in the M4-hiPSC line (Figures S2G–S2I).

Additionally, the epicardial-derived co-culture resulted in CM hypertrophy, an indicator of maturation, as seen by a significant increase in CM area (Figure 4C). This could indicate a specific role of epicardial-derived cells on CM hypertrophy not afforded by the EPCs. The increased CM area in the CM + EPC->DC condition is consistent across all three hiPSC-lines (Figure S5). Another morphologic indicator of maturation is the percent of multinucleation in hiPSC-CM as the adult human heart contains ∼26% multinucleated CM (Bergmann et al., 2015). No significant increases in multinucleation were seen in the epicardial co-cultures (Figure 4D). Though TGF-β inhibition caused a significant reduction in the percentage of multinucleated CM in the CM(+SB) condition, the presence of EPCs ameliorated this negative effect. The results were similar in all three hiPSC-lines (Figures S2G–S2I). In summary, in the CM + EPC(+SB) condition, CM showed increases in sarcomere length, while in the CM + EPC->DC condition, the CM showed increased hypertrophy; this outcome indicates differential effects of EPCs and EPDCs on hiPSC-CM phenotype.

Since the most significant maturation effects were seen in the CM + EPC(+SB) condition, transmission electron microscopy (TEM) was conducted on the CM + EPC(+SB) conditions and its control, CM(+SB), to better visualize CM ultrastructure as well as to quantify the size of mitochondria present. The mitochondria of CM + EPC(+SB) tended to be in direct apposition to sarcomeres, a characteristic of maturing CM, whereas the mitochondria of CM(+SB) were present most frequently in separate clusters (Dorn et al., 2015) (Figure 4E). The CM + EPC(+SB) condition had a significant increase in mitochondrial cross-sectional area in comparison to the CM(+SB) control (Figure 4F). Mature adult-CM sarcomere TEM images contain the distinct presence of Z-lines, I-bands, A-bands, and M-lines (Ronaldson-Bouchard et al., 2018). Both conditions have the distinct presence of Z-lines, I-bands, and A-bands but lack the presence of M-lines in CM (Figure 4G). The M-line typically appears last with long-term culture, 3D cultures, and extended exposure to electrical stimulation and so was not expected here (Ronaldson-Bouchard et al., 2018).

### scRNA-seq reveals a unique population of vCM in both epicardial co-cultures

**Figure 5:**
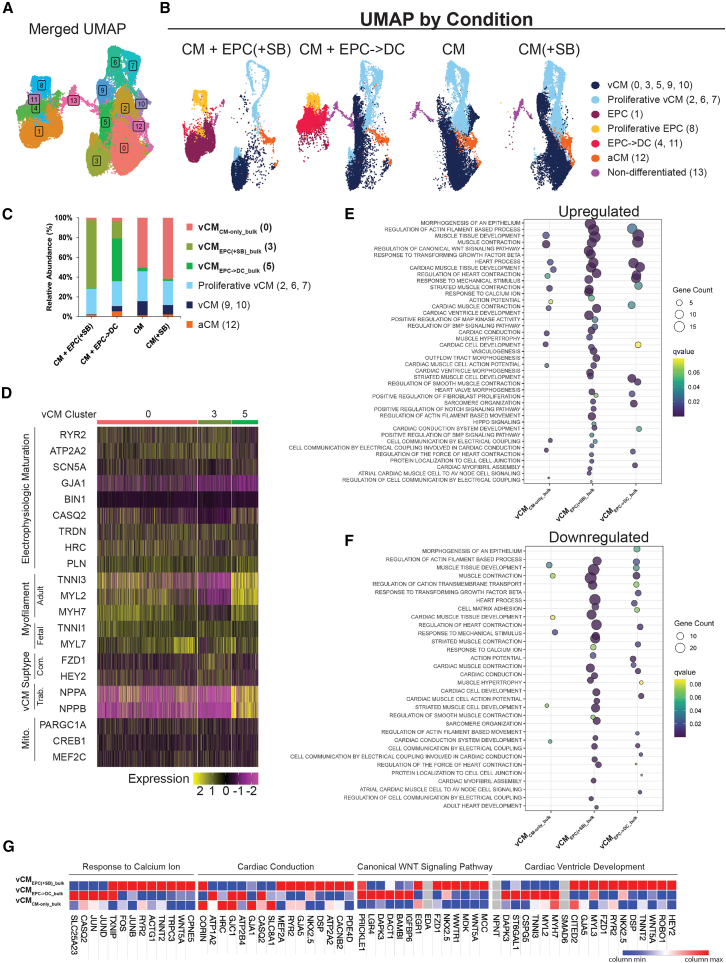
Single-cell RNA-seq reveals unique vCM phenotype in epicardial and epicardial derived co-cultures (A) Single-cell RNA-seq UMAP of clustering where 14 different clusters (0–13) were identified. (B) UMAP of each condition where the clusters were labeled into 7 major cell identities: ventricular CM (vCM; clusters 0, 3, 5, 9, and 10), proliferative vCM (clusters 2, 6 and 7), epicardial cells (EPCs; cluster 1), proliferative EPCs (cluster 8), epicardial to epicardial-derived cells (EPC->DC; clusters 4 and 11), atrial CM (aCM; cluster 12), and non-differentiated cells (cluster 13). (C) Graph of the percentage of CM that fall into each of the 6 CM categories listed. (D) Heatmap of cardiac genes of interest for the bulk vCM clusters present in the CM-only controls (cluster 0), CM + EPC(+SB) (cluster 3), and CM + EPC->DC (cluster 5). (E and F) ORA for biological processes (BP) showing a dot plot for the (E) upregulated and (F) downregulated pathways for the three bulk vCM clusters as compared to all other vCM clusters. (G) Heatmap of some of the differentially expressed genes contributing to enrichment in four BP pathways: (1) cardiac ventricle development; (2) regulation of canonical WNT signaling pathway; (3) cardiac conduction; and (4) response to calcium ions. See also Figures S3–S5.

To further understand the genetic landscape of the CM in the co-cultures scRNA-seq was conducted on all 4 conditions in duplicate. The cells from all the conditions were merged and 14 clusters were identified (Figure 5A). The proportions of cells in each cluster by conditions can be visualized in the supplemental information (Figure S3A). These were identified as seven cell type categories: the ventricular CM (vCM; clusters 0, 3, 5, 9, and 10), proliferative vCM (clusters 2, 6, and 7), EPC (cluster 1), proliferative EPC (cluster 8), EPC->DC (clusters 4 and 11), atrial CM (aCM; cluster 12), and non-differentiated cells (cluster 13) that represent the 1%–4% cTnT-negative population from the initial hiPSC-differentiation (Figure 5B). The identity of each cluster was determined by looking at the differentially expressed genes (DEGs) between each cluster (Figure S3B). In particular, the vCM expressed cardiac markers as well as the ventricular-specific myofilament isoform *MYL2*. The proliferative vCM had similar gene expression as the vCM but also expressed markers related to mitotic cell division, chromosome segregation, and/or cytokinesis. The aCM expressed general cardiac markers, *TNNT2*, and the atrial marker natriuretic peptide A (*NPPA*) but lacked expression of *MYL2*. The proportion of cell types across conditions shows a large population of EPC in the CM + EPC(+SB) and a genetically distinct population of cells emerging in the CM + EPC->DC conditions labeled EPC->DC (Figure S3C). To guide analysis, the 9 clusters expressing CM marker genes were grouped into 6 categories: (1) vCM_CM-only_bulk_ (cluster 0); (2) vCM_EPC(+SB)_bulk_ (cluster 3); (3) vCM_EPC->DC_bulk_ (cluster 5); (4) proliferative vCM (clusters 2, 6, and 7); (5) vCM (clusters 9 and 10), and (6) aCM (cluster 12) (Figure 5C). The majority of CM from the CM-only conditions fall into the vCM_CM-only_bulk_ category while 69% and 17% of the CM in the CM + EPC(+SB) and CM + EPC->DC conditions respectively fall into the vCM_EPC(+SB)_bulk_ category (cluster 3). The majority of the CM from the CM + EPC->DC condition falls into the vCM_EPC->DC_bulk_ category (Figure 5C). Separate clustering of vCM from the co-cultures indicated significant differences in gene expression stimulated by EPC and EPC->DC in the co-cultures.

To further understand these genetic shifts, genes related to CM maturation were examined, between the vCM_CM-only_bulk_, vCM_EPC(+SB)_bulk,_ and vCM_EPC->DC_bulk_ (clusters 0, 3, and 5). Largely consistent with functional characterization, the vCM_EPC(+SB)_bulk_ (cluster 3) showed an increase in the key electrochemical maturation genes ryanodine receptor 2 (*RYR2*), ATPase sarcoplasmic reticulum calcium transporter 2 (*ATP2A2*) and triadin (Figure 5D) (Clemens et al., 2023; Guo et al., 2023). Surprisingly, they also showed a marked decrease in other key electrochemical maturation genes, such as calsequestrin 2 (*CASQ2*), histidine-rich calcium-binding protein (*HRC*), and phospholamban (*PLN*) (Arvanitis et al., 2011; G. Chen et al., 2015; J. Liu et al., 2009). Additionally, though sarcomere length was increased in the CM + EPC(+SB) co-culture, there was a decrease in the adult ventricular myofilament isoforms cardiac muscle troponin I (*TNN13*), myosin light chain 2 ventricular form (*MYL2*), and myosin heavy chain 7 (*MYH7*). Nonetheless, the vCM were more specified showing increases in the compact ventricular markers frizzled 1 (*FZD1*) and the related family bHLH transcription factor with YRPW motif 2 (*HEY2*) along with decreases in trabecular ventricular myocardium markers NPPA and natriuretic peptide B (*NPPB*) (Funakoshi et al., 2021; Li et al., 2016). Conversely, the vCM_EPC->DC_bulk_ (cluster 5) showed a distinct increase in the adult myofilament isoforms *TNN13* and *MYL2* with an accompanying decrease in the fetal isoforms skeletal muscle troponin I (*TNNI1*) lacking in the vCM_CM-only_bulk_; this fits with the CM hypertrophy seen in the morphological assessment. Interestingly, the vCM_EPC->DC_bulk_ (cluster 5) also showed high expression of the trabecular myocardial markers *NPPA* and *NPPB*. These shifts corresponded to an increase in the electrophysiologic maturation marker *CASQ2* and *PLN* but decreases in *RYR2* and *ATP2A2*. These results indicate differential enhancement of electrophysiologic and myofilaments maturation in vCM spurred by either EPCs or EPDCs.

The vCM_CM-only_bulk_ did not have a defined up- or downregulation of compact or trabecular markers consistent with early fetal development before ventricular patterning is spurred by the endocardium and epicardium (Tian et al., 2017). The same heatmap was made for all the vCM pooled by condition (Figure S4A). In this iteration, the trends are the same as described previously, but the CM + EPC->DC condition has a portion of cells resembling the vCM_EPC(+SB)_bulk_ in addition to the vCM_EPC->DC_bulk_. This outcome is consistent with the composition of the CM + EPC->DC condition wherein most, but not all, EPCs undergo EMT. The proliferative vCM show a similar heatmap but with muted expression patterns due to the downregulation of cardiac muscle genes during the proliferation process (Figure S5A).

### Differentially expressed genes and pathways analysis of vCM in epicardial co-cultures

DEGs were determined between each vCM cluster (clusters 0, 3, 5, 9, and 10) (Figure S3D). The largest number of DEGs was present in the vCM_EPC(+SB)_bulk_ (cluster 3), followed by the vCM_EPC->DC_bulk_ (cluster 5, Table S1). Over-representation analysis (ORA) of gene ontology terms related to biological processes was performed, resulting in many pathways being up- and downregulated between the different vCM clusters (Table S1). For the vCM_EPC(+SB)_bulk_ multiple pathways related to calcium ion handling, cardiac conduction, and ventricular development were upregulated (Figure 5E). Conversely, some of these pathways were also downregulated, particularly those related to heart contraction due to decreases in myofilament genes MYL2 and TNNI3 (Figure 5F). Additionally, pathways related to key signaling cascades that occur during heart embryogenesis were upregulated in the vCM_EPC(+SB)_bulk_ such as WNT, bone morphogenic protein, Notch, and MAPK and Hippo signaling pathways. These signaling pathways are all highly interconnected and multiple of the same DEGs occur in each pathway. Of particular interest are the WNT signaling pathways because of the role they play in CM proliferation and specification of the compact myocardium *in vivo* and *in vitro* (Buikema et al., 2020; Fan et al., 2018; Funakoshi et al., 2021). The vCM_EPC->DC_bulk_ showed significant increases in pathways related to myofibrils and muscle contraction but downregulation in some conduction-related pathways (Figures 5E and 5F). The vCM_CM-only_bulk_ had the fewest upregulated pathways and among them were a few pathways related to conduction due to increased expression of *GJA1*, *SLC8A1*, and *HRC* (Figures 5E and 5F). A heatmap showing specific genes that were up- and downregulated in four of the enriched pathways shows some of the key DEGs that contributed to the enrichment (Figure 5G). DEGs and pathways enrichment was also done for the vCM by condition (Figures S4B–S4E; Table S1). The same pathways of interest were identified in this analysis. When the proliferative vCM were analyzed by condition, there were fewer DEGs and BP pathways that were enriched (Figures S5B–S5E; Table S1). However, the ones that were statistically significant confirm results seen when the vCM are analyzed by cluster and by condition.

Overall, these results indicate a significant genetic shift in vCM occurs when they are co-cultured with EPC or EPC->DCs. EPCs promote a more fetal-like but electrically conductive vCM population, which could be driven by the upregulation of the WNT signaling pathway. The EPC->DC co-cultures bring a population of vCM further along the developmental progression where they are electrically conductive (though with gene expression distinct from EPC co-cultures), beginning to express adult myofilament isoforms, and with a population of both trabecular and compact myocardium-like vCM. Next, we moved onto a 3D EHT model to study the effects of EPCs in a more complex system.

### CM proliferation and function in EHTs generated with epicardial and epicardial derived cells

**Figure 6:**
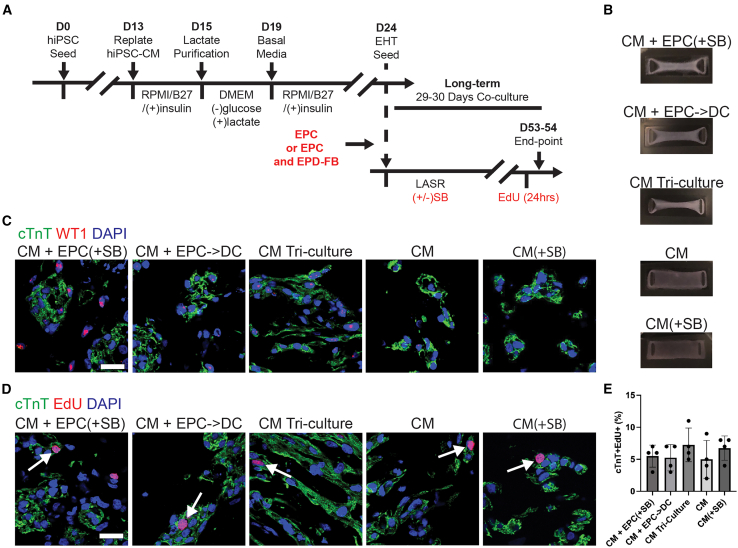
Epicardial engineered heart tissue proliferation (A) Experimental timeline for engineered heart tissue seeding where the red text represents experimental variables. (B) Images of engineered heart tissues at day 29 or 30 in PDMS molds. (C) Representative EHT images of WT1 (red), cTnT (green), and DAPI (blue). (D) Representative images of cTnT (green), EdU (red), and DAPI (blue) where the white arrows point to proliferating CM. (E) The quantified EHT CM proliferation via the percentage of cTnT^+^EdU^+^ cells. Scale bars, 20 μm. In (E), the bar and error bars represent the mean ± SD where each dot represents the average across three sections of one EHT and *n* = 4 EHTs across two independent experiments for each condition was assessed. ^∗^*p* < 0.05, ^∗∗^*p* < 0.01, ^∗∗∗^*p* < 0.001, and ^∗∗∗∗^*p* < 0.0001.

To determine whether the impact of EPCs on CM proliferation and maturation could persist in the context of the more intricate interplay of cell and matrix in a 3D tissue, EHTs were generated with the same four conditions used in 2D and assessed for proliferation and function after 29–30 days of culture (Figures 6A and 6B). In addition, a CM Tri-culture condition was added that included CM, EPCs, and fully differentiated epicardial-derived fibroblasts (EPD-FBs) that predominate in the postnatal myocardium. The results for this condition are described in detail later. After 29–30 days of co-culture and similar to 2D, EPCs were still present in the CM + EPC(+SB) condition and minimally present in all other conditions (Figure 6C). EHT proliferation was assessed with cryosections that were co-stained for cTnT and EdU (Figure 6D). After 30 days of culture, there were no differences in proliferation between any of the conditions (Figure 6E). The proliferation of all conditions was substantially decreased from the 2D short-term culture to an average of 5%–7% indicative of more advanced maturation of CM in 3D and a transient effect of EPCs on CM proliferation that eventually subsides in the context of 3D tissue development.

**Figure 7:**
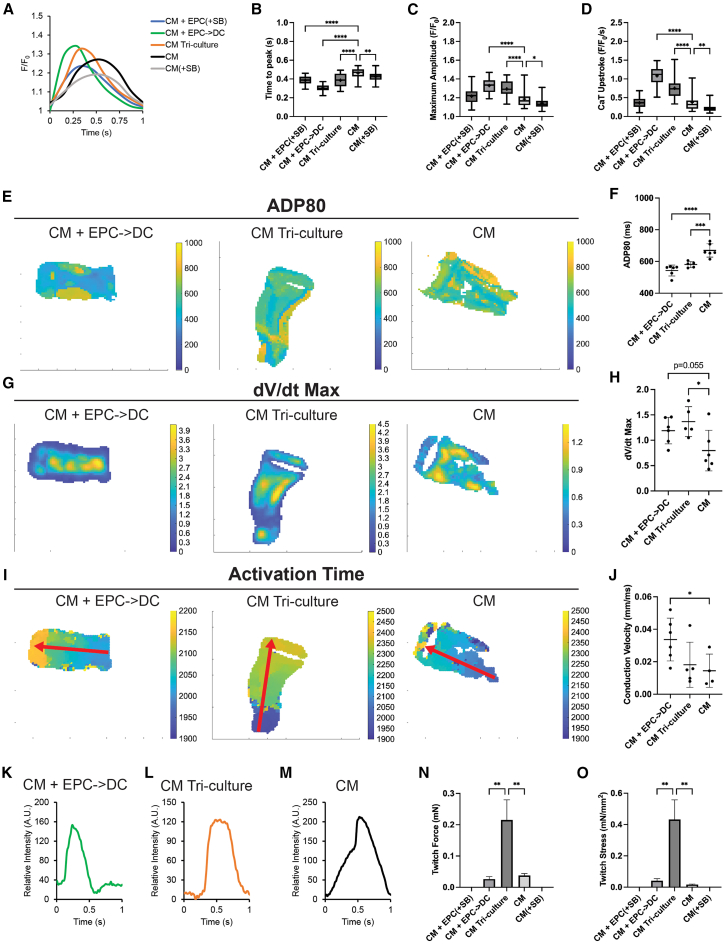
Epicardial cells enhance engineered heart tissue conduction while the CM tri-culture enhances electrochemical function and force (A–D) (A) Representative EHT calcium transient (CaT) traces and quantified (B) time to peak, (C) maximum amplitude, and (D) CaT upstroke velocity. (E and F) Voltage optical mapping for the CM + EPC->DC (left), CM Tri-culture (middle), and CM (right) control showing (E) representative APD80 maps and (F) quantified average APD80. (G and H) (G) Representative optical mapping maximum upstroke velocity maps and (H) average maximum upstroke velocity. (I) Optical mapping activation time maps showing signal propagation from where the point electrical stimulator was placed (end of the red arrow) to the other side of the EHT (point of the red arrow). The red arrow shows the distance and direction for which the conduction velocity was calculated across for these three EHTs. (J) The quantified conduction velocity for all EHTs that showed an analyzable conduction pattern. (K–M) Representative AP trace cropped to 1 s for the (K) CM + EPC->DC, (L) CM Tri-culture, and (M) CM conditions. (N and O) (N) The quantified twitch force for EHTs with a detectable force and those with no detectable force (N.D) as well as the (O) twitch stress. For (B and C), the data are represented as box and whisker plots with + marking the mean and *n* = 12 EHTs per condition from 3 independent experiments. For (F, H, and J), each dot represents the average across all pixels for one EHT, the center line is the mean, and the error bars represent the SD. For (F and H), *n* = 6 EHT across two independent experiments and for (J), *n* = 6 EHTs for CM + EPC->DC and CM Tr-culture and *n* = 4 for CM across two independent experiments. For (N and O), the bar graph represents the mean ± SEM and CM + EPC(+SB) (*n* = 10), CM + EPC->DC (*n* = 10), CM Tri-culture (*n* = 8), CM (*n* = 12), and CM(+SB) (*n* = 12) EHTs across 3 independent experiments were measured. ^∗^*p* < 0.05, ^∗∗^*p* < 0.01, ^∗∗∗^*p* < 0.001, and ^∗∗∗∗^*p* < 0.0001. See also Figure S7.

As in 2D, CaT measurements were taken from all five conditions (Figure 7A). Like 2D, all the co-culture conditions resulted in a robust decrease in time to peak indicating more mature calcium handling (Figure 7B). In 3D, the CM(+SB) condition also resulted in a slight decrease in time to peak but a marked decrease in all other parameters such as maximum amplitude and CaT upstroke velocity (Figures 7C and 7D). Differing from the 2D results, the long-term 3D co-cultures required epicardial-derived cells (CM + EPC->DC) to attain a significant increase in maximum amplitude and CaT upstroke velocity (Figures 7C and 7D). This effect may be driven by a combination of epicardial-derived signaling promoting maturation (Bargehr et al., 2019) and the absence of long-term TGF-β inhibition (Umbarkar et al., 2019), which was used in the CM + EPC(+SB) condition and may have impaired long-term function. To further explore the tissue level electrophysiologic maturation, optical mapping was conducted for the CM + EPC->DC condition in comparison to a CM-only control. Maps of APD80 showed extended APD80 in the ventricular CM range for all conditions (Figure 7E). Upon quantification, the longest APD80 was present in the CM-only control (Figure 7F). The maximum upstroke velocity (dV/dt Max) was mapped and quantified, showing a strong but not significant trend upwards in the CM + EPC->DC condition (*p* = 0.055) (Figures 7G and 7H). Lastly, activation time maps were plotted to determine if there was signal propagation from one end of the EHT to another with point electrical stimulation (Figure 7I). If propagation was present, the conduction velocity across the EHT could be calculated. The conduction velocity showed a significant increase in the CM + EPC->DC condition (Figure 7J). Representative AP traces show a more regular ventricular AP shape of the co-culture AP and the reduced upstroke present in some of the CM-only APs (Figures 7K–7M).

Tissue level mechanical function was determined by assessing the twitch force of the EHTs. The long-term incubation with TGF-β inhibitors was detrimental to force generation and both conditions maintained in TGF-β inhibitors did not have a detectable force (Figure 7N). Both CM and CM + EPC->DC either had no detectable force or produced small forces in the 0.01–0.02 mN range (Figure 7N). A similar trend was seen in twitch stress generation (Figure 7O). Taken together, these data largely mirror those of 2D. While EPCs play a role in electrophysiologic maturation in 3D, they do not support robust tissue-level force generation.

### Addition of an epicardial-derived fibroblast population in combination with EPCs results in improved force and electrophysiologic function

To determine the impact of a more advanced embryonic-to-fetal developmental stage, EPD-FBs were added to EHTs containing CM and EPCs (CM Tri-culture). In particular, EPCs were maintained in TGF-β inhibitors for 9 days, mimicking the 2D condition, and then the TGF-β inhibitors were removed allowing epicardial EMT. It was necessary to differentiate the EPD-FB separately as the EPCs used in this study lacked *TCF21* and did not spontaneously undergo EMT into fibroblasts on their own. The lack of FB in the EPC->DC conditions was validated by staining for TE7, a fibroblast marker, in the 2D and 3D co-cultures (Figures S6A–S6C). The addition of fibroblasts did not change CM proliferation in the EHTs (Figure 6E). When CaT were assessed for the CM Tri-culture, similar improvements were present as in the CM + EPC->DC conditions (Figures 7A–7D). Videos of the calcium handling for all five conditions can viewed in Video S1. Calcium transients of CM + EPC(+SB) EHT, Video S2. Calcium transients of CM + EPC->DC EHT, Video S3. Calcium transients of CM Tri-culture EHT, Video S4. Calcium transients of CM EHT, Video S5. Calcium transients of CM(+SB) EHT. Optical mapping of voltage determined a significant decrease in the average EHT APD80 as well as a significant increase in the AP dV/dt Max (Figures 7E–7H). All CM Tri-culture EHTs measured did have a measurable conduction velocity (Figures 7I and 7J). A representative AP trace of the CM Tri-Culture EHTs shows a similar shape to the CM + EPC->DC condition (Figure 7L). Most significantly, the addition of the fibroblasts to the EHTs resulted in an order-of-magnitude increase in twitch force and stress generation (Figures 7N and 7O). Taken together, when the last major cellular component of myocardial development is added to engineered cardiac mimics containing EPCs and EPC->DCs, proliferation is lost, electrophysiology is maintained, and force generation is significantly enhanced.

Video S1. Calcium transients of CM + EPC(+SB) EHT

Video S2. Calcium transients of CM + EPC->DC EHT

Video S3. Calcium transients of CM Tri-culture EHT

Video S4. Calcium transients of CM EHT

Video S5. Calcium transients of CM(+SB) EHT

### Further phenotypic evaluation of EHTs reveals enhanced tissue level morphologic maturation and restoration of adult sarcomeric isoforms in CM Tri-culture EHTs

To evaluate CM morphologic maturation in EHTs, tissue cryosections were stained with α-actinin and DAPI to assess CM sarcomere length (Figure S7A). As in 2D, the EPCs resulted in a slight but significant increase in sarcomere length that was present in both co-culture conditions (Figure S7B). The CM Tri-culture condition resulted in the largest increase in sarcomere length. Additionally, cellular alignment was evaluated in the EHTs using phalloidin. All co-cultures increased cellular alignment, but the largest increase was present in the CM Tri-culture: the phalloidin coherency is 0 when cells are randomly aligned and 1 when perfectly aligned (Figure S7C). To evaluate the level of myofibril adult/fetal isoform switching, RT-qPCR was used to determine the ratio of *MYH7* to *MYH6* expression (Figure S7D). We found a significant decrease in the *MYH7/MYH6* ratio in both the epicardial and epicardial-derived co-cultures. The presence of EPD-FBs ameliorated this effect and led to a slight increase in the *MYH7/MYH6* ratio.

Some key markers of electrophysiologic maturation were also assessed. The presence of *GJA1* was significantly increased in the CM + EPC(+SB) condition, possibly because the EPCs themselves were expressing large amounts of *GJA1* as determined by scRNA-seq (Figure S7E). There were no significant differences in the expression levels of *ATP2A2* and *KCNJ2* detected between any of the co-cultures with the CM controls (Figures S7F and S7G). Additionally, EPCs alone did not influence the bulk *RYR2* expressions but the CM Tri-culture conditions had a significant increase compared to the CM-only controls (Figure S7H). *PLN*, a regulator of SERCA2 or *ATP2A2*, was significantly decreased in both the epicardial co-cultures but this effect was ameliorated with the addition of fibroblasts in the CM Tri-culture condition (Figure S7J). To assess protein-level expression, we performed immunostaining for RYR2 and SERCA2 in EHT cryosections. We observed a significant increase in RYR2 expression in both conditions containing EPCs. In contrast, SERCA2 expression remained unchanged, consistent with the RT-qPCR results for *ATP2A2*. In summary, while EPCs spur a more fetal but electrochemically functional phenotype, the addition of EPD-FBs pushes the tissues one step further by sustaining electrochemical function and enhancing mechanical function.

## Discussion

Intrigued by embryonic development and the lack of knowledge with regard to CM maturation during this stage, we investigated murine hearts during E10, E12, and E17 and found that substantial amounts of CM maturation occur at this time. We then studied the effect of EPCs on ventricular CM *in vitro* and found that they simultaneously spurred a more electrochemically functional but fetal-like phenotype characterized by increases in proliferation and expression of fetal myofilament isoforms. Single-cell sequencing revealed the upregulation of multiple signaling pathways, including WNT, that are implicated in CM proliferation and compact myocardium specification during development. The increased electrochemical function was validated in 3D-EHTs. To mimic the multicellularity present during development, fully differentiated EPD-FBs were added to the EHTs in a CM Tri-culture. These tissues showed robust electrochemical maturation and force generation as well as increased adult myofilament isoforms. Taken together, we found that mimicking the multicellular progression of the fetal to late neonatal environment best supports hiPSC-CM functional maturation. These results leave us to speculate whether EPCs play a role in electrochemical maturation during *in vivo* development and, more importantly, whether embryonic development is the missing stage of hiPSC-CM maturation *in vitro*.

Here we show that EPCs are powerful mediators of hiPSC-CM electrochemical function. Enhanced calcium handling and AP parameters were seen in both 2D and 3D co-cultures. Some of the potential mediators for this increase were determined to be *RYR2* and *ATP2A2*, as they were significantly upregulated in the EPC(+SB) co-culture. *RYR2* is the dominant calcium channel that releases calcium from the sarcoplasmic reticulum. In a recent mouse model, a RYR2-depleted hearts showed functional deficits, decreased presence of T-tubules, and decreased morphologic maturation (CM sarcomere length, CM area, and elongation) (Guo et al., 2023). Therefore, the epicardial-induced *RYR2* expression could point to the mechanism for morphologic maturation increases as well as more mature calcium handling. Conversely, *ATP2A2* is responsible for bringing calcium back into the sarcoplasmic reticulum. This could be related to the significant increase in CaT downstroke velocity seen in the EPC(+SB) co-cultures. CACNB2 encodes the predominant β-subunit for cardiac L-type calcium channel; CM-specific knockout (KO) of this gene leads to reduced L-type calcium current and vascular dysfunction leading to embryonic lethality (Weissgerber et al., 2006). L-Type calcium currents are regulators of excitation-contraction coupling, and this could serve as another mediator for enhanced conduction seen in the epicardial co-cultures. When *RYR2* and *ATP2A2* were looked at in the EHTs, no changes were detected; this could be due to the loss of single-cell resolution in dense cultures and the challenge of finding a stable cardiac gene to normalize to in these highly heterogeneous cultures. In the epicardial-derived co-culture, *CASQ2* was significantly upregulated. This is the most abundant calcium-binding protein of the sarcoplasmic reticulum. This could be responsible for increased calcium handling seen in these conditions as genetic over-expression of *CASQ2* in hiPSC-CM has this effect (Liu et al., 2009).

Studies of CM electrophysiology at early time points in development are scarce. Multiple studies show that cardiac calcium handling and AP parameters shift between embryonic, post-natal, and adult phases of development but few encompass multiple points of embryonic development (Liu et al., 2002; Swift et al., 2020; Ziman et al., 2010). However, one study looking at mice hearts encompassed three embryonic time points as well as multiple post-natal time points (Peinkofer et al., 2016). Interestingly, the largest increase in AP amplitude, coupled with the largest decrease in MDP occurred between E9-10 and E12-14 of embryonic development. This corresponds to the developmental time point where EPCs have covered the heart and substantial amounts of EMT have occurred (Acharya et al., 2012; Vicente-Steijn et al., 2015; Von Gise et al., 2011). Though this is by no means evidence of epicardial-induced CM electrochemical maturation *in vivo,* it provides the rationale for future study. It also provides evidence that electrochemical maturation does occur during embryonic development and that by immediately pushing post-natal conditions in hiPSC-CM we might be missing this critical phase of maturation.

The parallel findings of increased maturation and proliferation in this study are intriguing as CM proliferation and maturation are described as dichotomous (Singh et al., 2023). This may indeed be the case *in vivo* as our murine data suggest CM proliferation does decrease as sarcomere length and cell area increase. It is important to note that the *in vitro* case is highly artificial in the way that it can exist without the epicardium since mouse models without the epicardium will not survive. It is also important to note that mouse embryonic CM maturation seen in this study occurs while CM proliferation is extremely high (∼50%), meaning some amount of maturation does happen *in vivo* while CM are still in a highly proliferative state. The increases in proliferation we see in parallel with maturation could simply be a result of more accurately mimicking *in vivo* cardiac multicellularity. Additionally, the simultaneous increase in proliferation and calcium handling has been seen in previous *in vitro* hiPSC-CM and epicardial co-cultures (Tan et al., 2021). Taken together with the embryonic patch clamp data discussed previously, there is evidence to suggest that proliferation and aspects of CM maturation, such as electrochemical function, do occur simultaneously *in vitro*. Unlike the 2D co-cultures, the 3D EHTs containing EPCs did not have enhanced proliferation after 29–30 days of co-culture. This was the first study that looked at co-cultures for this long and it is very likely that an increase in CM proliferation would be temporal and eventually subside as the 3D environments promotes enhanced CM maturation. Future studies are needed to look at the temporal aspects of epicardial-induced CM proliferation to determine if a transient enhancement is present in the EHTs.

Our data suggest that mechanical maturation as well as the robust expression of adult myofilament isoforms likely occurs after CM-proliferation and some aspects of electrochemical maturation have occurred. We are the first to report that EPCs, before EMT, drive more fetal CM myofilament expression. Interestingly, epicardial-derived cells (in 2D) and EPD-FBs (in 3D) ameliorated this effect resulting in increased TNNI3 and MYL2 expression in 2D and an increase in the MYH7/MYH6 ratio in 3D. This finding fits with the progression seen in embryonic development where EPCs spur the expansion of CM, which leads to the formation of the dense compact myocardium in fetal development, and then myofilament isoform switching does not occur until postnatal maturation (Lu et al., 2022; Vicente-Steijn et al., 2015). The finding that myofilament maturation cannot occur while CM are proliferative is not necessarily new as it is commonly postulated that the highly organized nature of mature sarcomeres provides a mechanical barrier for cell division as the complex myofilament bundles must disassemble for cytokinesis to occur. However, the uncoupling of sarcomere length and myofilament isoform switching in the proliferation maturation dichotomy is a new finding as we found the longest sarcomere length in the CM + EPC(+SB) condition. Our murine data support this uncoupling as substantial increases in murine sarcomere length occur *in vivo* while the CM are still highly proliferative at a time point in embryonic development where fetal myofilament isoform expression is still dominant (DeLaughter et al., 2016; Lu et al., 2022).

Our TFM data also suggest that myofilament isoform expression is not necessarily related to hiPSC-CM single-cell force generation. TFM reported no significant changes in single cell force generation even though fetal isoforms prevailed in the CM + EPC(+SB) co-culture. Further, sarcomere length seemed to be a better predictor of increased force generation as the only conditions with a significantly different force were the CM + EPC(+SB) and the CM(+SB) conditions, which had the highest and lowest sarcomere length, respectively. This finding is supported by another study where single hiPSC-CM contractility and APs were looked at in a reporter system allowing visualization of MYH6 and MYH7 (Weber et al., 2020). This study found that the myosin heavy chain isoform expression did not correlate to CM mechanical or electrophysiologic function (Weber et al., 2020). In our study, the most mature electrochemical function was seen in the population determined to have a more fetal myofilament composition (CM + EPC[+SB]). Taken together, our results beg the question of whether this “intermediate” stage of development should be achieved before post-natal conditions are imposed on the hiPSC-CM. If we push myofilament switching and reductions in CM proliferation too soon, will proper CM maturation ever be achieved? Here, we provide evidence that mimicking embryonic development can have a powerful impact on the maturation state of the CM. Future studies could impose additional post-natal conditions, such as electrical stimulation, increased afterload, and a switch to fatty acid/glucose depleted media after the imposition of embryonic developmental cues.

Here, we also report the first single-cell sequencing experiment of hiPSC-CM and EPCs in co-culture. We found that EPCs have enormous effects on the phenotype of the hiPSC-CM and vice versa. In addition to the shift in calcium handling and myofilament genes discussed previously, these results also pointed to multiple signaling cascades that were only upregulated in the epicardial co-cultures. Among these, the WNT signaling cascade is of interest because of the role it has been seen to play in spurring CM proliferation *in vivo* and *in vitro* (Buikema et al., 2020; Fan et al., 2018). WNT signaling was increased in both the vCM and the EPCs from the co-culture conditions. Additionally, CM proliferation seen *in vivo* is also coupled with the appearance of the compact myocardium, a portion of the myocardium lacking in epicardial KO mice models (Vicente-Steijn et al., 2015). Interestingly, small molecule WNT activation has also been used to induce expression of the compact myocardium markers *HEY2* and *FZD2* in hiPSC-CM (Funakoshi et al., 2021). These markers, along with increased CM proliferation, were both significantly increased here in the CM co-cultured with EPCs(Funakoshi et al., 2021). This indicates WNT as a potential mechanism for EPC-induced proliferation and compact myocardium specification. In development, the growth factor IGF2 has also been seen to mediate CM proliferation/ventricular compaction (Brade et al., 2011; Li et al., 2011). This mechanism could possibly converge on WNT signaling as well since IGF2 has been seen to activate *WNT5A* expression and the WNT signaling pathway in cancer cells (Belharazem et al., 2016). *WNT5A* is a DEG in that was upregulated in the vCM of our CM + EPC(+SB) co-cultures. This indicates that *WNT5A* could be explored as the potential mediator of IGF2 and WNT-induced CM proliferation perhaps as a more refined and less variable approach to promote CM proliferation, compact myocardium specification, and potentially even electrochemical functionality, than the use of EPC co-culture.

Taken together, this study is the first to describe the robust electrochemical maturation conferred by pure populations of EPCs. It also holds significant implications for the field of cardiac tissue engineering as it identifies the embryonic phase of CM maturation as a unique and perhaps critical component of hiPSC-CM maturation. As developmental biology was mimicked to determine the pathways involved in CM-specification to yield robust hiPSC-CM differentiation protocols, the temporal modulation of CM embryonic maturation cues followed by post-natal cues could be envisioned for the realization of a fully functional adult phenotype in hiPSC-CM.

## Methods

### 2D co-culture construction and characterization

Briefly, hiPSCs were maintained on Matrigel in mTesR1. The hiPSCs were differentiated into hiPSC-CM and EPCs using WNT modulation. After differentiation, the hiPSC-CM were seeded onto Matrigel; 24 h later, the EPCs were seeded on top of the hiPSC-CM at a ratio of 25% EPCs to 75% hiPSC-CM. The co-cultures were maintained with or without TGF-β inhibitors (SB) for 8 day, and then assessed via flow cytometry, for hiPSC-CM morphology, calcium transients, patch clamp, TFM, TEM, and scRNA-seq. Detailed methods for the characterization of these co-cultures and the maintenance and differentiation of the hiPSCs that make up the co-cultures are described in the supplemental methods.

### Engineered heart tissue construction and characterization

The EHTs are constructed by combining hiPSC-CM and EPCs or EPCs and EPD-FBs (CM Tri-culture) in media with fibrinogen and thrombin to form a fibrin gel. The EHTs are then maintained in media containing trypsin inhibitors for 29–31 days until they were assessed for force generation via a force transducer, calcium transients, protein expression via immunohistochemistry (IHC), and gene expression via RT-qPCR. The detailed methods for EHT construction and characterization are outlined in the supplemental methods.

### Statistical analyses

All statistical analyses were done in PRISM using a one-way or two-way ANOVA and post-hoc Fisher least significant difference comparison of means. Significance was indicated as follows: ^∗∗∗∗^*p* ≤ 0.0001, ^∗∗∗^*p* ≤ 0.001, ^∗∗^*p* ≤ 0.01, and ^∗^*p* ≤ 0.05.

## Resource availability

### Lead contact

The data supporting this manuscript can be found in the figures of the manuscript and supplemental materials or by request to the lead contact, Brenda M. Ogle (ogle@umn.edu).

### Material availability

No new materials were generated in the making of this manuscript.

### Data and code availability

The single-cell sequencing data are available on GEO: GSE293435. The original code generated for this manuscript is available on GitHub: Sanaz081/OM-Stem-Cell-Analysis. FAIR and CARE data management practices were followed.

## Acknowledgments

The authors would like to awknowledge our funding sources RO1 HL137204, RO1 HL160779, NSF GRFP 2019272039, and the University of Minnesota Doctoral Dissertation Fellowship. The authors would like to thank the University of Minnesota 3D Bioprinting Facility for use of the Mach-1 Micromechanical Tissue Tester; Caleb Vogt for assembly of the Mach-1 for EHT mounting and force measurements; the University of Minnesota Genomics Center (UMGC) for single-cell library preparation and sequencing; Noah Stanis for calcium transient MATLAB code; and the University of Minnesota Imaging Center (UIC) for instruction, housing, and maintenance of the Olympus FluoView IX2 Inverted Confocal Microscope.

## Author contributions

S.E.G. designed the study, performed the experiments, analyzed the data, and wrote the manuscript. A.A.A., R.W., X.K., T.M.R., S.H., A.X., M.S., and A.A.T. performed experimentation and analysis. S.F.B., M.J., and N.C.M. performed analysis. B.N.S., S.D., P.W.A., E.G.T., and J.H.v.B. contributed to the interpretation of data and writing of the manuscript. B.M.O. contributed to experimental design, interpretation of data and writing of the manuscript.

## Declaration of interests

The authors declare no competing interests.

## Supplemental information

Document S1. Figures S1–S9, Tables S1–S3, and supplemental methods

Document S2. Article plus supplemental information
