# Supplementary material for: Developmental cues from epicardial cells simultaneously promote cardiomyocyte proliferation and electrochemical maturation
Source: Stem Cell Reports. 2025 Jul 3;20(8):102572. doi: 10.1016/j.stemcr.2025.102572 (PMC12365841; doi:10.1016/j.stemcr.2025.102572)
Supplement: Document S1. Figures S1–S9, Tables S1–S3, and supplemental methods [file mmc1.pdf]

Stem Cell Reports, Volume *20*

Supplemental Information

Developmental cues from epicardial cells simultaneously promote cardiomyocyte proliferation and electrochemical maturation

Sophie E. Givens, Abygail A. Andebrhan, Ruchen Wang, Xiangzhen Kong, Taylor M. Rothermel, Sanaz Hosseini, An Xie, Mohammad Shameem, Andrea A. Torniainen, Somayeh Ebrahimi-Barough, Samuel F. Boland, Maya Johnson, Natalia Calixto Mancipe, Bhairab N. Singh, Samuel Dudley, Patrick W. Alford, Elena G. Tolkacheva, Jop H. van Berlo, and Brenda M. Ogle

Supplemental Materials: Developmental cues from epicardial cells simultaneously promote cardiomyocyte proliferation and electrochemical maturation

Sophie E. Givens[1], Abygail A. Andebrhan[1], Ruchen Wang[2], Xiangzhen Kong[1], Taylor M. Rothermel[1], Sanaz Hosseini[3], An Xie[2], Mohammad Shameem[1,7], Andrea A. Torniainen[2], Somayeh Ebrahimi-Barough[1], Samuel F. Boland[1], Maya Johnson[1], Natalia Calixto Mancipe[8], Bhairab N. Singh[1,4,7], Samuel Dudley[2], Patrick W. Alford[1], Elena G. Tolkacheva[1,2,3,6], Jop H. van Berlo[2,4], Brenda M. Ogle[1,4,5,6]

[1]Biomedical Engineering, University of Minnesota, Minneapolis, MN, USA
[2]Lillehei Heart Institute (LHI), Department of Medicine, University of Minnesota, Minneapolis, MN, USA
[3]Electrical Engineering, University of Minnesota, Minneapolis, MN, USA
[4]Stem Cell Institute, University of Minnesota, Minneapolis, MN, USA
[5]Department of Pediatrics, University of Minnesota, Minneapolis, MN, USA
[6]Institute of Engineering in Medicine, University of Minnesota, MN, USA
[7]Department of Rehabilitation Medicine, University of Minnesota, MN, USA
[8]Minnesota Supercomputing Institute, University of Minnesota, MN, USA

Supplemental Figures and Legends

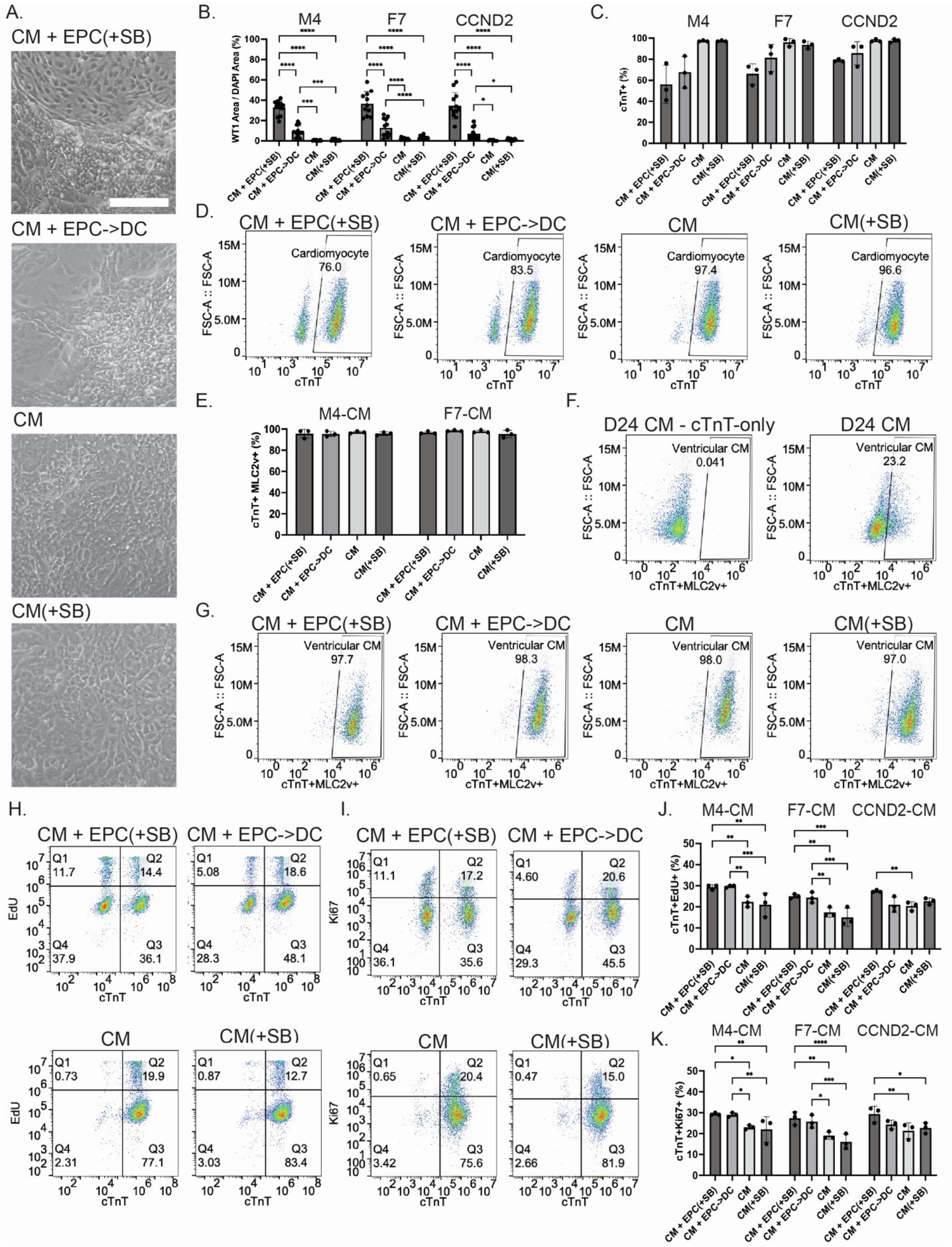

Figure S1 Co-culture composition and proliferation by line, related to Figure 2.

A) Brightfield images of all co-culture conditions at D35. Scale 50 µm. B) Quantification of WT1 Area / DAPI area indicating the percentage of epicardial cells in each co-culture condition broken down by line. C) Quantification of the percentage cTnT+ population from flow cytometry data broken down by lines. D) Representative flow cytometry data for cTnT. E) Quantification of the cTnT+MLC2v+ population for both lines quantified. F) Flow cytometry controls showing the cTnT+ and MLC2v on the x-axis with the day 24 CM stained for cTnT only (left) and D24 CM before treatment with ascorbic acid (right). G) Representative flow cytometry chart of the cTnT+MLC2v+ population for each co-culture condition at the experimental endpoint. H) Representative flow cytometry plots for each co-culture condition with cTnT on the x-axis and EdU on the y-axis as well as another set with I) cTnT on the x-axis and Ki67 on the y-axis. J) Quantification of flow cytometry for cardiomyocyte proliferation showing the percent cTnT+EdU+ population as well as K) the percent cTnT+Ki67+ population. (B-C), (E) and (J-K) Depict bar graphs and error bars representing the mean ± STDEV for n = 3 independent experiments for each condition. *$p < 0.05$, **$p < 0.01$, ***$p < 0.001$, and ****$p < 0.0001$ for (B).

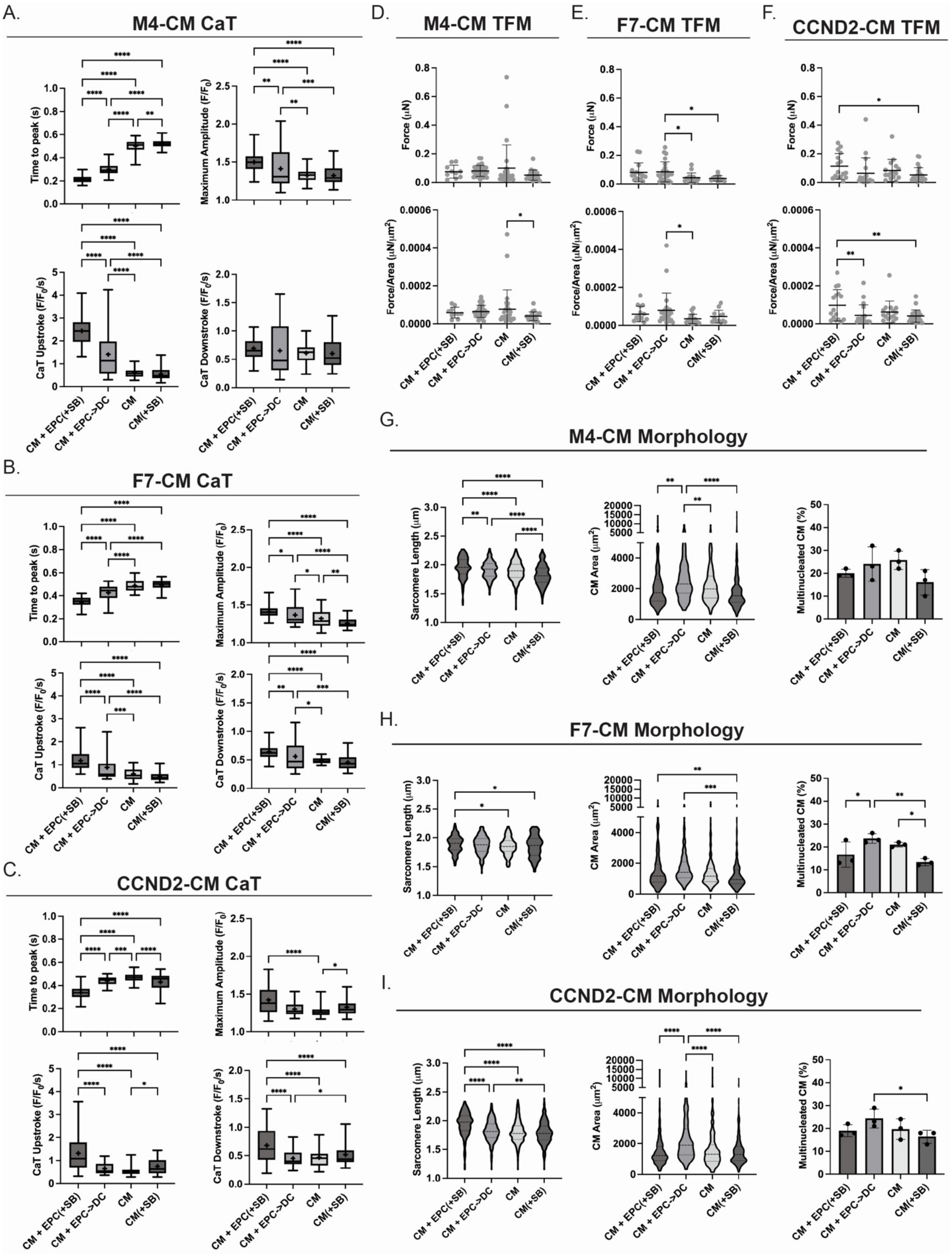
A.
M4-CM CaT
Time to peak (s)
Maximum Amplitude (F/F$_0$)
CaT Upstroke (F/F$_0$/s)
CaT Downstroke (F/F$_0$/s)
CM + EPC(+SB)
CM + EPC->DC
CM
CM(+SB)
B.
F7-CM CaT
C.
CCND2-CM CaT
D.
M4-CM TFM
E.
F7-CM TFM
F.
CCND2-CM TFM
Force (μN)
Force/Area (μN/μm^2)
G.
M4-CM Morphology
Sarcomere Length (μm)
CM Area (μm^2)
Multinucleated CM (%)
H.
F7-CM Morphology
I.
CCND2-CM Morphology

Figure S2. 2D Calcium transients, traction force microscopy, and CM morphology by hiPSC-line, related to Figures 3 and 4.

Quantification of the calcium transient parameters time to peak, maximum amplitude, upstroke, and downstroke velocity under 1 Hz electrical stimulation broken down for the A) M4 B) F7, and C) CCND2 hiPSC-lines. The traction force microscopy for D) M4-CM, E) F7-CM, and F) CCND2-CM showing the force (top) and the force normalized by cell area (bottom) for each co-culture condition. The morphologic assessment of CM replated onto glass slides at D35 and fixed in diastole 24hrs later showing quantification of CM sarcomere length (left), area (middle), and the percent multinucleated (right) for G) M4-CM, H) F7-CM and I) CCND2-CM. For (A-C) each graph is a box and whisker plot where the (+) represents the mean and the data represents three fields of view per well from three wells per replicate and n = 3 independent experiments per line. For (D-F) each data point represents one cardiomyocyte, and the center line and error bars represent the mean ± STDEV across n = 3 independent experiments. For (D) CM + EPC(+SB) (n = 10), CM + EPC->DC (n = 31), CM (n = 28) and CM(+SB) (n = 19). For (E) CM + EPC(+SB) (n = 15), CM + EPC->DC (n = 24), CM (n = 16) and CM(+SB) (n = 12) and for (F) CM + EPC(+SB) (n = 16), CM + EPC->DC (n = 19), CM (n = 16) and CM(+SB) (n = 23). For (G-I) the sarcomere length and area violin plot center dashed line represents the median and outer dashed lines represent upper and lower quartiles. The multinucleated percent bar graph represented the mean ± STDEV where each data point is the average percent of multinucleated CM across three wells and 5 fields of view per well for each independent experimental replicate with n = 3 independent experimental replicates per condition. In (G) for sarcomere length CM + EPC(+SB) (n = 276), CM + EPC->DC (n = 286), CM (n = 272) and CM(+SB) (n = 224) and for CM area CM + EPC(+SB) (n = 275), CM + EPC->DC (n = 280), CM (n = 270) and CM(+SB) (n = 224). In (H) for sarcomere length CM + EPC(+SB) (n = 202), CM + EPC->DC (n = 265), CM (n = 229) and CM(+SB) (n = 181) and for CM area CM + EPC(+SB) (n = 292), CM + EPC->DC (n = 192), CM (n = 263) and CM(+SB) (n = 284). In (I) for sarcomere length CM + EPC(+SB) (n = 410), CM + EPC->DC (n = 393), CM (n = 407) and CM(+SB) (n = 409) and for CM area CM + EPC(+SB) (n = 416), CM + EPC->DC (n = 388), CM (n = 503) and CM(+SB) (n = 413). *p < 0.05, **p < 0.01, ***p < 0.001, and ****p < 0.0001.

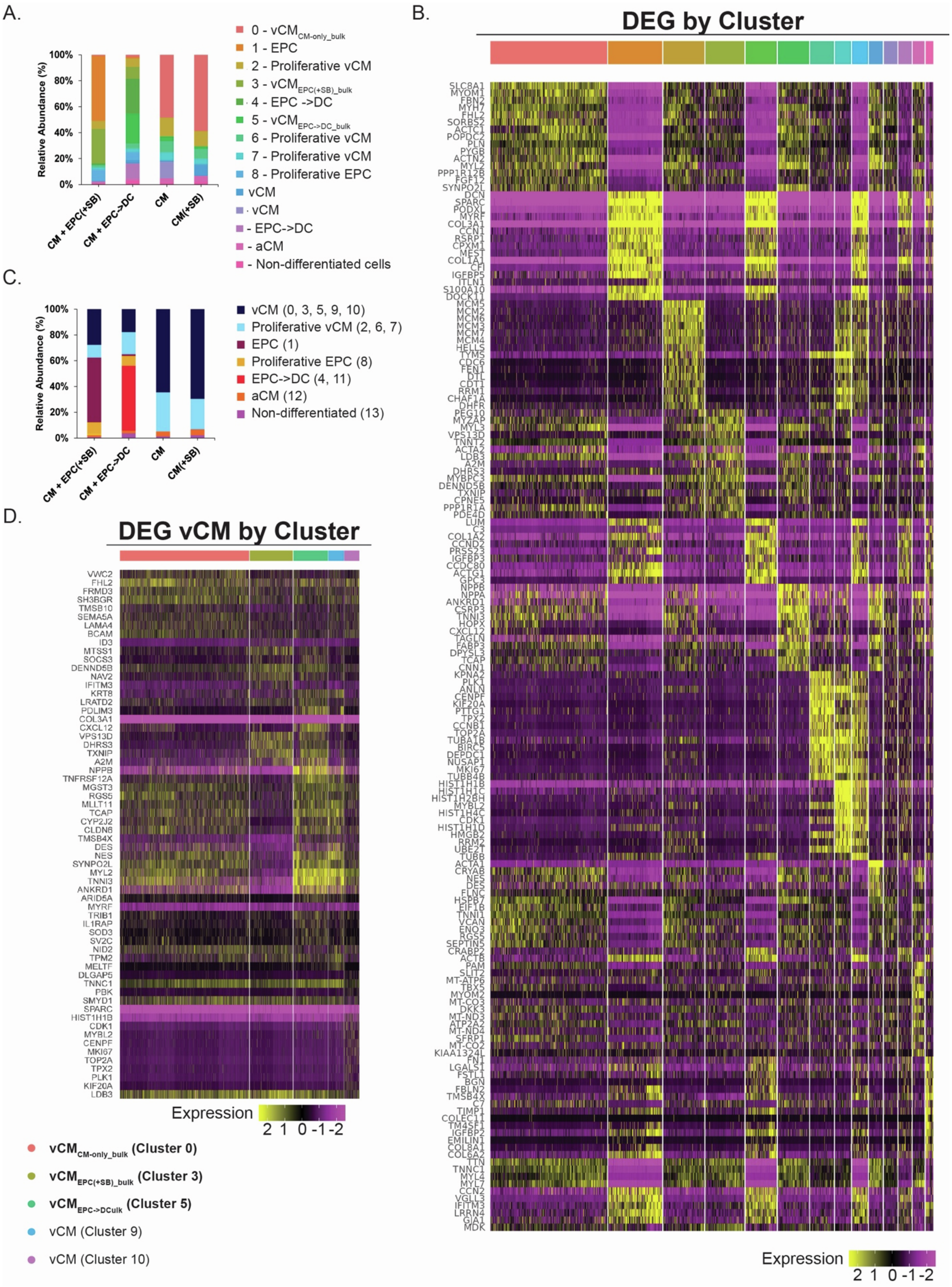

A.
100%
80%
60%
40%
20%
0%
Relative Abundance (%)
CM + EPC(+SB)
CM + EPC->DC
CM
CM(+SB)
0 - vCMCM-only_bulk
1 - EPC
2 - Proliferative vCM
3 - vCMEPC(+SB)_bulk
4 - EPC ->DC
5 - vCMEPC->DC_bulk
6 - Proliferative vCM
7 - Proliferative vCM
8 - Proliferative EPC
vCM
· vCM
- EPC->DC
- aCM
- Non-differentiated cells
B.
DEG by Cluster
SLC8A1
MYOM1
FBN2
MYH7
FHL2
SORBS2
ACTC1
POPDC2
PLN
PYGB
ACTN2
MYL2
PPP1R12B
FGF12
SYNPO2L
DCN
SPARC
PODXL
MYRF
COL3A1
CCN1
RSPR1
CPXM1
MEST
COL1A1
CFI
IGFBP5
ITLN1
S100A10
DOCK11
MCM5
MCM2
MCM6
MCM3
MCM7
MCM4
HELLS
TYMS
CDC6
FEN1
DTL
CDT1
RRM1
CHAF1A
DHFR
PEG10
MYZAP
MYL3
VPS13D
TNNT2
ACTA2
LDB3
A2M
DHRS3
MYBPC3
DENND5B
TXNIP
CPNE5
PPP1R1A
PDE4D
LUM
C3
COL1A2
CCND2
PRSS23
IGFBP3
CCDC80
ACTG1
GPC3
NPPB
NPPA
ANKRD1
CSRP3
TNNI3
HOPX
CXCL12
TAGLN
FABP3
DPYSL3
TCAP
CNN1
KPNA2
PLK1
ANLN
CENPF
KIF20A
PTTG1
TPX2
CCNB1
TOP2A
TUBA1B
BIRC5
DEPDC1
NUSAP1
MKI67
TUBB4B
HIST1H1B
HIST1H1C
HIST1H2BH
MYBL2
HIST1H4C
CDK1
HIST1H1D
HMGB2
RRM2
UBE2T
TUBB
ACTA1
CRYAB
NES
DES
FLNC
HSPB7
EIF1B
TNNI1
VCAN
ENO3
RGS5
SEPTIN5
CRABP2
ACTB
PAM
SLIT2
MT-ATP6
TBX5
MYOM2
MT-CO3
DKK3
MT-ND3
ATP2A2
MT-ND4
SFRP1
MT-CO2
KIAA1324L
FN1
LGALS1
FSTL1
BGN
FBLN2
TMSB4X
C7
TIMP1
COLEC11
TM4SF1
IGFBP2
EMILIN1
COL3A1
COL6A2
TTN
TNNC1
MYL4
MYL7
CCN2
VGLL3
IFITM3
LRRN4
GJA1
MDK
Expression
2 1 0 -1 -2
C.
100%
80%
60%
40%
20%
0%
Relative Abundance (%)
CM + EPC(+SB)
CM + EPC->DC
CM
CM(+SB)
vCM (0, 3, 5, 9, 10)
Proliferative vCM (2, 6, 7)
EPC (1)
Proliferative EPC (8)
EPC->DC (4, 11)
aCM (12)
Non-differentiated (13)
D.
DEG vCM by Cluster
VWC2
FHL2
FRMD3
SH3BGR
TMSB10
SEMA5A
LAMA4
BCAM
ID3
MTSS1
SOCS3
DENND5B
NAV2
IFITM3
KRT8
LRATD2
PDLIM3
COL3A1
CXCL12
VPS13D
DHRS3
TXNIP
A2M
NPPB
TNFRSF12A
MGST3
RGS5
MLLT11
TCAP
CYP2J2
CLDN6
TMSB4X
DES
NES
SYNPO2L
MYL2
TNNI3
ANKRD1
ARID5A
MYRF
TRIB1
IL1RAP
SOD3
SV2C
NID2
TPM2
MELTF
DLGAP5
TNNC1
PBK
SMYD1
SPARC
HIST1H1B
CDK1
MYBL2
CENPF
MKI67
TOP2A
TPX2
PLK1
KIF20A
LDB3
Expression
2 1 0 -1 -2
vCMCM-only_bulk (Cluster 0)
vCMEPC(+SB)_bulk (Cluster 3)
vCMEPC->DCulk (Cluster 5)
vCM (Cluster 9)
vCM (Cluster 10)

Figure S3. Single-cell RNAseq clustering of CM co-cultures, related to Figure 5.

A) Graph showing the proportions of cells from each condition in each cluster. B) A heat map of the top 15 differentially expressed genes (DEG) for each cluster. C) Graph showing the proportion of cells from each condition categorized by the 7 main labels 1) vCM (Cluster 0, 3, 5, 9 and 10), proliferative vCM (Cluster 2, 6 and 7), EPC (Cluster 1), EPC->DC (Cluster 4 and 11), aCM (Cluster 12) and non-differentiated cells (Cluster 13). D) A heat map of the top 15 differentially expressed genes between the 5 vCM clusters (Cluster 0, 3, 5, 9, and 10).

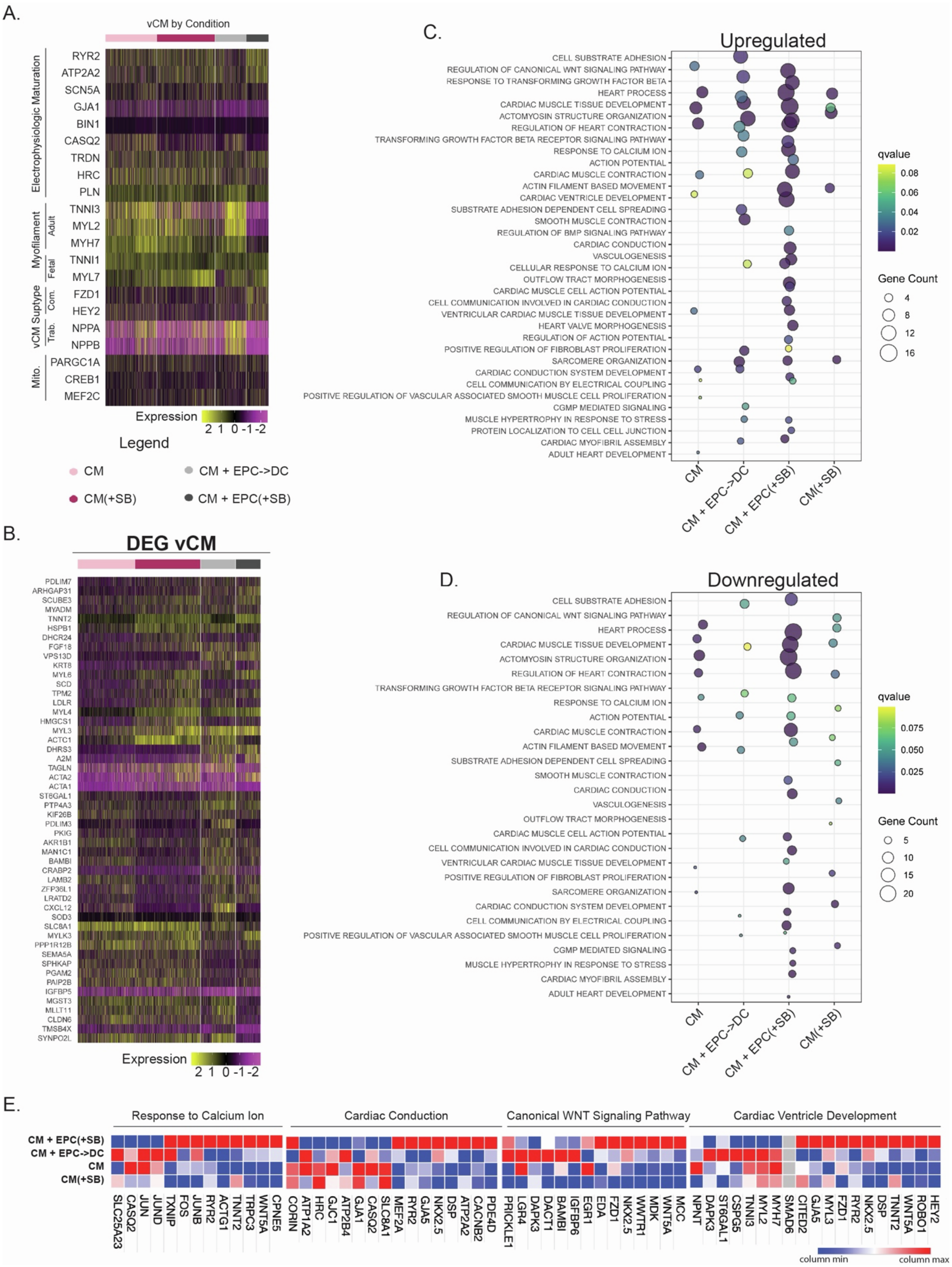
A.
vCM by Condition
Electrophysiologic Maturation
RYR2
ATP2A2
SCN5A
GJA1
BIN1
CASQ2
TRDN
HRC
PLN
Myofilament
Adult
TNNI3
MYL2
MYH7
Fetal
TNNI1
MYL7
vCM Suptype
Com.
FZD1
HEY2
Trab.
NPPA
NPPB
Mito.
PARGC1A
CREB1
MEF2C
Expression
2 1 0 -1 -2
Legend
CM
CM + EPC->DC
CM(+SB)
CM + EPC(+SB)
B.
DEG vCM
PDLIM7
ARHGAP31
SCUBE3
MYADM
TNNT2
HSPB1
DHCR24
FGF18
VPS13D
KRT8
MYL6
SCD
TPM2
LDLR
MYL4
HMGCS1
MYL3
ACTC1
DHRS3
A2M
TAGLN
ACTA2
ACTA1
ST6GAL1
PTP4A3
KIF26B
PDLIM3
PKIG
AKR1B1
MAN1C1
BAMBI
CRABP2
LAMB2
ZFP36L1
LRATD2
CXCL12
SOD3
SLC8A1
MYLK3
PPP1R12B
SEMA5A
SPHKAP
PGAM2
PAIP2B
IGFBP5
MGST3
MLLT11
CLDN6
TMSB4X
SYNPO2L
Expression
2 1 0 -1 -2
C.
Upregulated
CELL SUBSTRATE ADHESION
REGULATION OF CANONICAL WNT SIGNALING PATHWAY
RESPONSE TO TRANSFORMING GROWTH FACTOR BETA
HEART PROCESS
CARDIAC MUSCLE TISSUE DEVELOPMENT
ACTOMYOSIN STRUCTURE ORGANIZATION
REGULATION OF HEART CONTRACTION
TRANSFORMING GROWTH FACTOR BETA RECEPTOR SIGNALING PATHWAY
RESPONSE TO CALCIUM ION
ACTION POTENTIAL
CARDIAC MUSCLE CONTRACTION
ACTIN FILAMENT BASED MOVEMENT
CARDIAC VENTRICLE DEVELOPMENT
SUBSTRATE ADHESION DEPENDENT CELL SPREADING
SMOOTH MUSCLE CONTRACTION
REGULATION OF BMP SIGNALING PATHWAY
CARDIAC CONDUCTION
VASCULOGENESIS
CELLULAR RESPONSE TO CALCIUM ION
OUTFLOW TRACT MORPHOGENESIS
CARDIAC MUSCLE CELL ACTION POTENTIAL
CELL COMMUNICATION INVOLVED IN CARDIAC CONDUCTION
VENTRICULAR CARDIAC MUSCLE TISSUE DEVELOPMENT
HEART VALVE MORPHOGENESIS
REGULATION OF ACTION POTENTIAL
POSITIVE REGULATION OF FIBROBLAST PROLIFERATION
SARCOMERE ORGANIZATION
CARDIAC CONDUCTION SYSTEM DEVELOPMENT
CELL COMMUNICATION BY ELECTRICAL COUPLING
POSITIVE REGULATION OF VASCULAR ASSOCIATED SMOOTH MUSCLE CELL PROLIFERATION
CGMP MEDIATED SIGNALING
MUSCLE HYPERTROPHY IN RESPONSE TO STRESS
PROTEIN LOCALIZATION TO CELL CELL JUNCTION
CARDIAC MYOFIBRIL ASSEMBLY
ADULT HEART DEVELOPMENT
CM
CM + EPC->DC
CM + EPC(+SB)
CM(+SB)
qvalue
0.08
0.06
0.04
0.02
Gene Count
4
8
12
16
D.
Downregulated
CELL SUBSTRATE ADHESION
REGULATION OF CANONICAL WNT SIGNALING PATHWAY
HEART PROCESS
CARDIAC MUSCLE TISSUE DEVELOPMENT
ACTOMYOSIN STRUCTURE ORGANIZATION
REGULATION OF HEART CONTRACTION
TRANSFORMING GROWTH FACTOR BETA RECEPTOR SIGNALING PATHWAY
RESPONSE TO CALCIUM ION
ACTION POTENTIAL
CARDIAC MUSCLE CONTRACTION
ACTIN FILAMENT BASED MOVEMENT
SUBSTRATE ADHESION DEPENDENT CELL SPREADING
SMOOTH MUSCLE CONTRACTION
CARDIAC CONDUCTION
VASCULOGENESIS
OUTFLOW TRACT MORPHOGENESIS
CARDIAC MUSCLE CELL ACTION POTENTIAL
CELL COMMUNICATION INVOLVED IN CARDIAC CONDUCTION
VENTRICULAR CARDIAC MUSCLE TISSUE DEVELOPMENT
POSITIVE REGULATION OF FIBROBLAST PROLIFERATION
SARCOMERE ORGANIZATION
CARDIAC CONDUCTION SYSTEM DEVELOPMENT
CELL COMMUNICATION BY ELECTRICAL COUPLING
POSITIVE REGULATION OF VASCULAR ASSOCIATED SMOOTH MUSCLE CELL PROLIFERATION
CGMP MEDIATED SIGNALING
MUSCLE HYPERTROPHY IN RESPONSE TO STRESS
CARDIAC MYOFIBRIL ASSEMBLY
ADULT HEART DEVELOPMENT
CM
CM + EPC->DC
CM + EPC(+SB)
CM(+SB)
qvalue
0.075
0.050
0.025
Gene Count
5
10
15
20
E.
Response to Calcium Ion
Cardiac Conduction
Canonical WNT Signaling Pathway
Cardiac Ventricle Development
CM + EPC(+SB)
CM + EPC->DC
CM
CM(+SB)
SLC25A23 CASQ2 JUN JUND TXNIP FOS JUNB RYR2 ACTG1 TNNT2 TRPC3 WNT5A CPNE5
CORIN ATP1A2 HRC GJC1 ATP2B4 GJA1 CASQ2 SLC8A1 MEF2A RYR2 GJA5 NKX2.5 DSP ATP2A2 CACNB2 PDE4D
PRICKLE1 LGR4 DAPK3 DACT1 BAMBI IGFBP6 EGR1 EDA FZD1 NKX2.5 WWTR1 MDK WNT5A MCC
NPNT DAPK3 ST6GAL1 CSPG5 TNNI3 MYL2 MYH7 SMAD6 CITED2 GJA5 MYL3 FZD1 RYR2 NKX2.5 DSP TNNT2 WNT5A ROBO1 HEY2
column min
column max

Figure S4. Pooled vCM by condition DEG and ORA, related to Figure 5.

A) A heat map of cardiac genes of interest for all the vCM broken down by condition. B) Heat map of the top 15 differentially expressed genes between the vCM broken down by condition. Dot plots showing some of the ORA biological processes (BP) pathways that were significantly C) upregulated or D) downregulated in the vCM by conditions. E) Heat map of some of the differentially expressed genes contributing to enrichment in four GO BP pathways 1) Cardiac Ventricle Development, 2) Regulation of Canonical WNT Signaling Pathway, 3) Cardiac Conduction, and 4) Response to Calcium Ions.

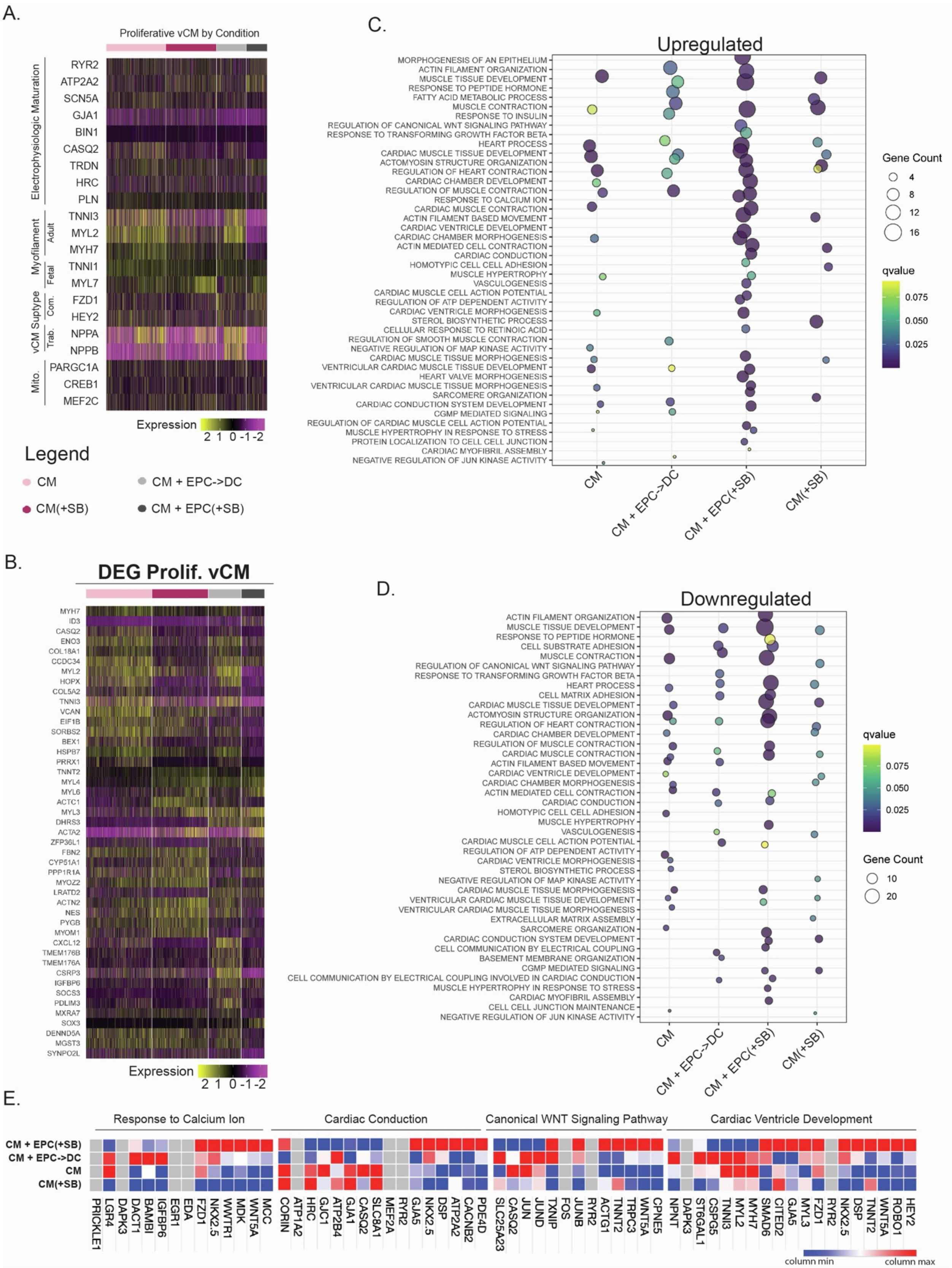
A.
Proliferative vCM by Condition
Electrophysiologic Maturation
RYR2
ATP2A2
SCN5A
GJA1
BIN1
CASQ2
TRDN
HRC
PLN
Myofilament
Adult
TNNI3
MYL2
MYH7
Fetal
TNNI1
MYL7
vCM Suptype
Com.
FZD1
HEY2
Trab.
NPPA
NPPB
Mito.
PARGC1A
CREB1
MEF2C
Expression
2 1 0 -1 -2
Legend
CM
CM + EPC->DC
CM(+SB)
CM + EPC(+SB)
C.
Upregulated
Gene Count
4
8
12
16
qvalue
0.075
0.050
0.025
CM
CM + EPC->DC
CM + EPC(+SB)
CM(+SB)
B.
DEG Prolif. vCM
Expression
2 1 0 -1 -2
D.
Downregulated
qvalue
Gene Count
10
20
E.
Response to Calcium Ion
Cardiac Conduction
Canonical WNT Signaling Pathway
Cardiac Ventricle Development
CM + EPC(+SB)
CM + EPC->DC
CM
CM(+SB)
column min
column max

Figure S5. Pooled proliferative vCM by condition DEG and ORA, related to Figure 5.

A) A heat map of cardiac genes of interest for all the proliferative vCM broken down by condition. B) Heat map of the top 15 differentially expressed genes between the proliferative vCM broken down by condition. Dot plots showing some of the ORA biological processes (BP) pathways that were significantly C) upregulated or D) downregulated in the proliferative vCM by conditions. E) Heat map of some of the differentially expressed genes contributing to enrichment in four GO BP pathways 1) Cardiac Ventricle Development, 2) Regulation of Canonical WNT Signaling Pathway, 3) Cardiac Conduction, and 4) Response to Calcium Ions.

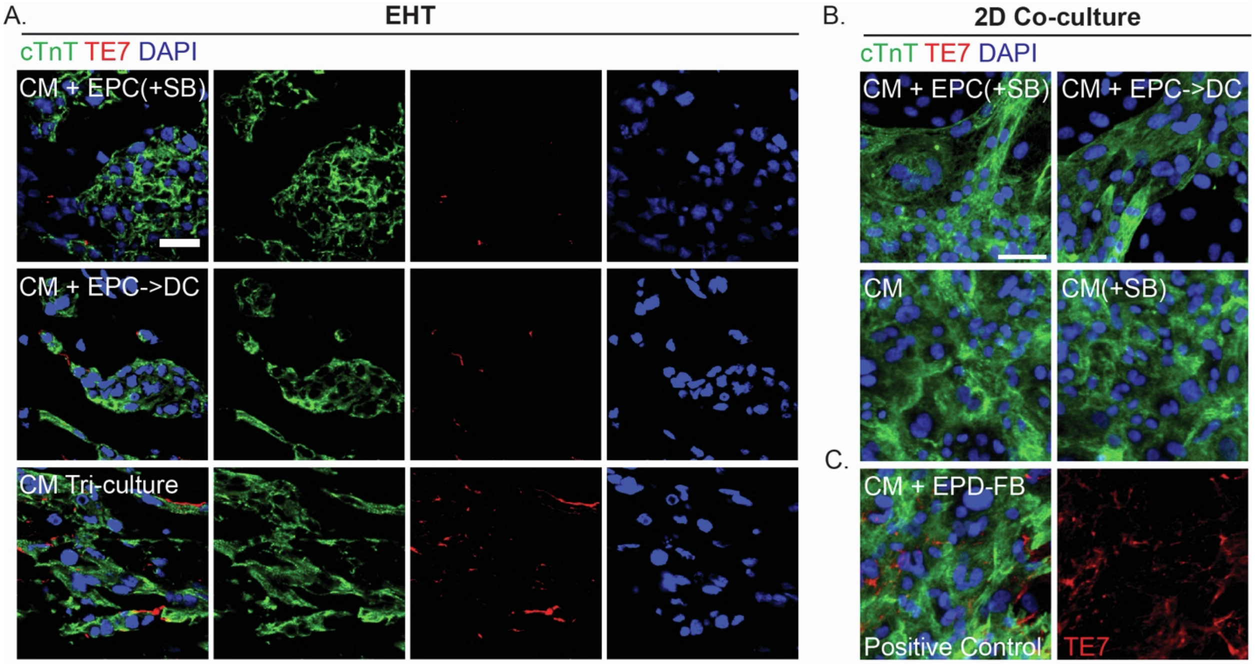

Figure S6. Engineered heart tissues and 2D co-culture fibroblasts content.

A) Staining of cTnT (green – cardiomyocyte), TE7 (red – fibroblasts), and DAPI (blue – nucleus) in EHT sections from the CM + EPC(+SB) (top), CM + EPC->DC (middle) and CM Tri-culture (bottom) conditions. Scale 20 µm. B) Staining of cTnT (green – CM), TE7 (red – fibroblasts), and DAPI (blue – nucleus) in all four 2D co-culture conditions and a C) positive control where fully differentiated EPD-FBs were co-cultured with CM in 2D. Scale 50 µm.

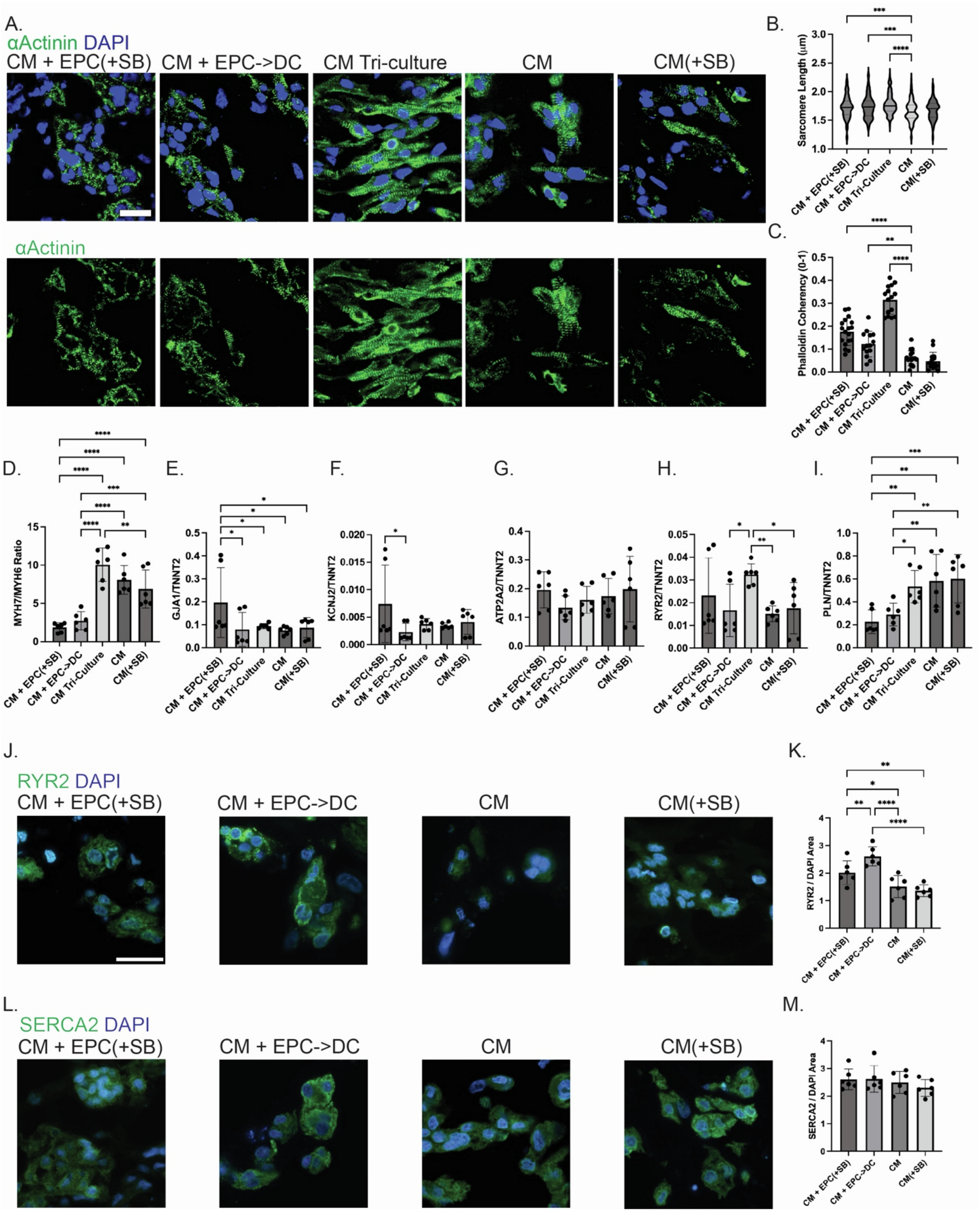
A.
αActinin DAPI
CM + EPC(+SB)
CM + EPC->DC
CM Tri-culture
CM
CM(+SB)
αActinin
B.
Sarcomere Length (μm)
C.
Phalloidin Coherency (0-1)
D.
MYH7/MYH6 Ratio
E.
GJA1/TNNT2
F.
KCNJ2/TNNT2
G.
ATP2A2/TNNT2
H.
RYR2/TNNT2
I.
PLN/TNNT2
J.
RYR2 DAPI
CM + EPC(+SB)
CM + EPC->DC
CM
CM(+SB)
K.
RYR2 / DAPI Area
L.
SERCA2 DAPI
CM + EPC(+SB)
CM + EPC->DC
CM
CM(+SB)
M.
SERCA2 / DAPI Area

Figure S7. EHT Phenotypic maturation, related to Figure 7.

A) Representative images of αActinin (green) and DAPI (blue) in EHT cryosections. Scale 50 μm. B) Quantified sarcomere length and C) Cellular alignment in the EHTs as shown by the average coherency of phalloidin from EHT transverse cross-sections where 0 is randomly aligned and 1 is perfectly aligned. Quantitative rt-PCR of the D) *MYH6/MYH7* ratio and E) *GJA1*, F) KCNJ2, G) *ATP2A2* H) *RYR2,* and I) *PLN* normalized to the housekeeping gene *EDF1* and the cardiac gene *TNNT2*. J) Representative images of RYR2 (green) and DAPI (blue) in EHT cryosections with K) quantification of RYR2 area normalized to DAPI area. L) Representative images of SERCA2 (green) and DAPI (blue) in EHT cryosections with M) quantification of RYR2 area normalized to DAPI area. For (B) violin plot center dashed line represents the median and outer dashed lines represent the upper and lower quartiles of the distribution and CM + EPC(+SB) (n = 162), CM + EPC->DC (n = 174), CM Tri-culture (n = 191), CM (n = 145), CM(+SB) (n = 125) sarcomeres were measured across three sections per EHT and 6 EHTs per condition across three independent experiments. For (C) the phalloidin coherency was determined for CM + EPC(+SB) (n = 18), CM + EPC->DC (n = 14), CM Tri-culture (n = 15), CM (n = 17), CM(+SB) (n = 15) EHT cross-sections across 6 EHTs per conditions from three independent experiments. For (D-I) each dot represents one technical replicate from n = 3 EHTs per condition across three independent experiments. For (K) and (M), each dot represents a field view across two EHTs for three replicates. See also **Table S3**.

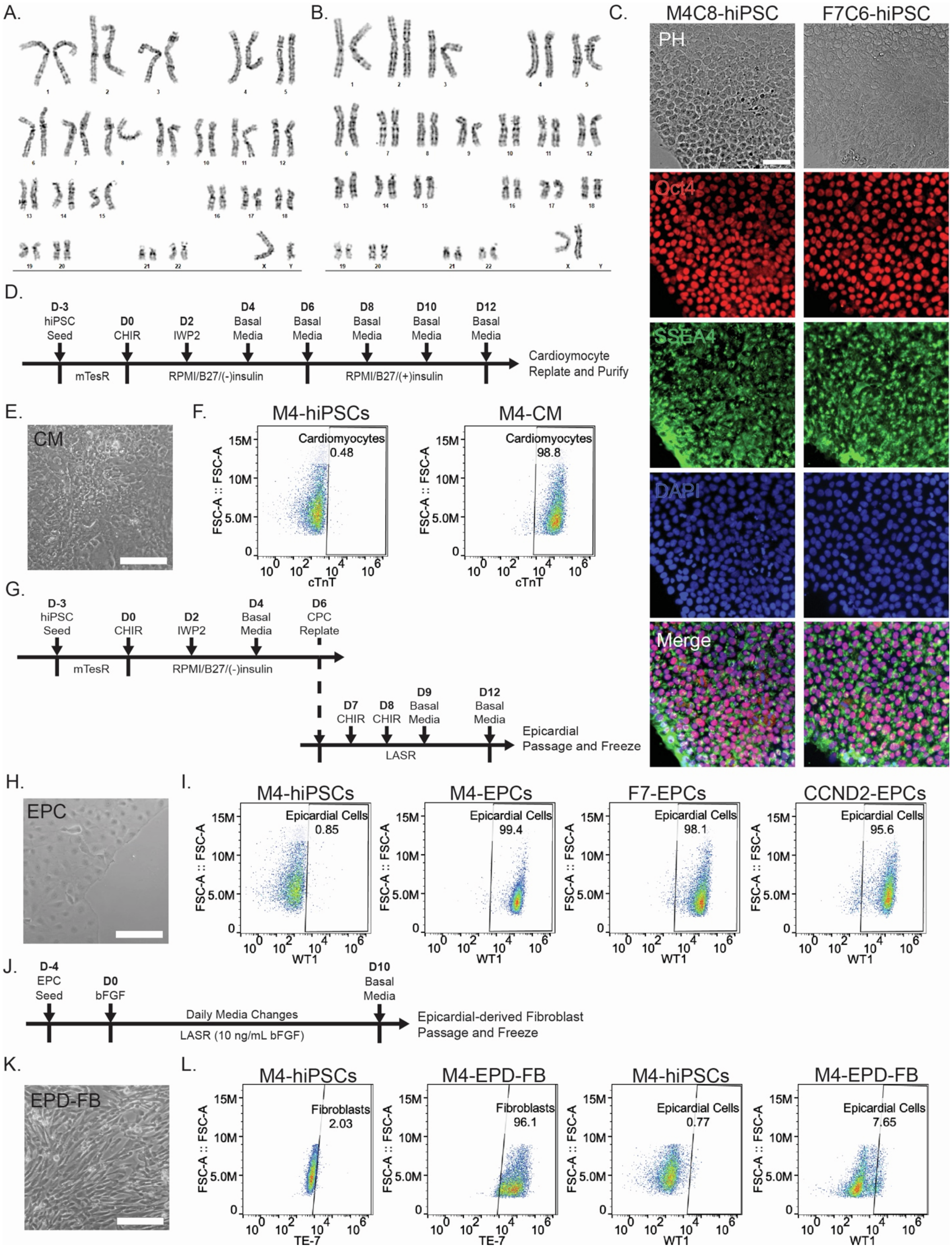
A.
B.
C.
M4C8-hiPSC
F7C6-hiPSC
PH
Oct4
SSEA4
DAPI
Merge
D.
D-3 hiPSC Seed
D0 CHIR
D2 IWP2
D4 Basal Media
D6 Basal Media
D8 Basal Media
D10 Basal Media
D12 Basal Media
mTesR
RPMI/B27/(-)insulin
RPMI/B27/(+)insulin
Cardioymocyte Replate and Purify
E.
CM
F.
M4-hiPSCs
M4-CM
Cardiomyocytes 0.48
Cardiomyocytes 98.8
FSC-A :: FSC-A
cTnT
G.
D-3 hiPSC Seed
D0 CHIR
D2 IWP2
D4 Basal Media
D6 CPC Replate
mTesR
RPMI/B27/(-)insulin
D7 CHIR
D8 CHIR
D9 Basal Media
D12 Basal Media
LASR
Epicardial Passage and Freeze
H.
EPC
I.
M4-hiPSCs
M4-EPCs
F7-EPCs
CCND2-EPCs
Epicardial Cells 0.85
Epicardial Cells 99.4
Epicardial Cells 98.1
Epicardial Cells 95.6
WT1
J.
D-4 EPC Seed
D0 bFGF
D10 Basal Media
Daily Media Changes
LASR (10 ng/mL bFGF)
Epicardial-derived Fibroblast Passage and Freeze
K.
EPD-FB
L.
M4-hiPSCs
M4-EPD-FB
Fibroblasts 2.03
Fibroblasts 96.1
Epicardial Cells 0.77
Epicardial Cells 7.65
TE-7
WT1

Figure S8. M4 and F7-hiPSC Pluripotency and Karyotype and the differentiation and purity of hiPSC-EPC, EPD-FB, and CM.

The full karyotype of the A) M4-hiPSC lines and the B) F7-hiPSC lines. C) Representative images of the M4-hiPSC line (left) and the F7-hiPSC line (right) stained for the pluripotency markers Oct4 (red) and SSEA4 (green) and a nuclear stain DAPI. D) Schematic of the hiPSC-CM small molecule differentiation protocol. E) Brightfield image of D24 CM post-replating and lactate purification as well as F) flow cytometry for CM before engineered heart tissue seeding using M4-hiSPCs as a negative control for cTnT gating. G) Schematic of the hiPSC-EPC small molecule differentiation protocol. H) A brightfield image of the hiPSC-EPCs and I) flow cytometry for the epicardial marker WT1 for M4-hiPSCs as a negative control and P2-4 EPCs from the M4, F7, and CCND2 hiPSC-lines used for this study. J) A schematic of the hiPSC-EPD-FB differentiation protocol and K) a brightfield image of the EPD-FBs. L) Flow cytometry for the fibroblast marker TE7 to assess the purity of the EPF-FB differentiation using M4-hiPSCs as a negative control for gating. Scale 50 µm.

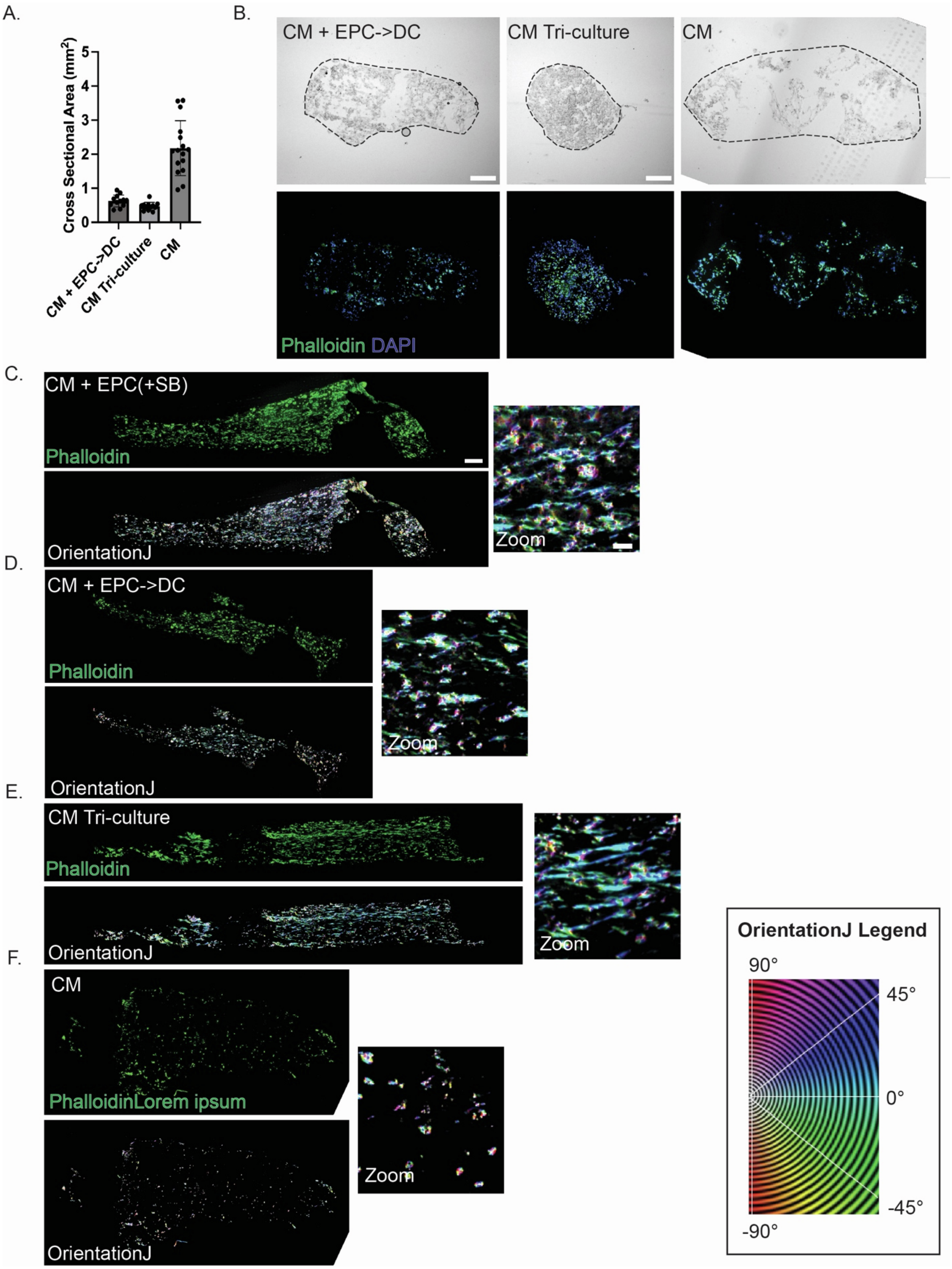
A.
B.
Cross Sectional Area (mm²)
5
4
3
2
1
0
CM + EPC->DC
CM Tri-culture
CM
CM + EPC->DC
CM Tri-culture
CM
Phalloidin DAPI
C.
CM + EPC(+SB)
Phalloidin
OrientationJ
Zoom
D.
CM + EPC->DC
Phalloidin
OrientationJ
Zoom
E.
CM Tri-culture
Phalloidin
OrientationJ
Zoom
F.
CM
PhalloidinLorem ipsum
OrientationJ
Zoom
OrientationJ Legend
90°
45°
0°
-45°
-90°

Figure S9. EHT cross-sectional area measurements for twitch force calculation and EHT phalloidin staining and OrientationJ coherency measurements, related to Figure S7.
A) A bar graph of the average cross-sectional area for each of the force-generating EHT conditions. B) A bright field image with the cross-sectional area of the EHTs highlighted, followed by phalloidin (green) and DAPI (blue) images for better cellular visualization within the EHT cross-sections. Scale 250 μm. Representative transverse cross-sections of EHTs showing F-actin (green - phalloidin - top) and the orientation color map (bottom) showing the average angle of each F-actin fiber. For the C) CM + EPC(+SB), D) CM + EPC->DC, E) CM Tri-culture and F) CM EHTs. Scale 250 μm for whole sections and 20 μm for zoomed-in fields of view.

Supplemental Tables

Table S1 Single-cell RNA sequencing vCM integration DEG and BP pathways analysis, related to Figure 5.

Comparison	Condition	# of DEG	# BP Pathways UP	# BP Pathways DOWN
**vCM by Cluster**	Cluster 0	85	31	2
**vCM by Cluster**	Cluster 3	323	234	147
**vCM by Cluster**	Cluster 5	246	47	81
**vCM by Cluster**	Cluster 9	153	84	4
**vCM by Cluster**	Cluster 10	246	109	131
**vCM by Condition**	CM + EPC(+SB)	332	193	158
**vCM by Condition**	CM + EPC->DC	189	65	9
**vCM by Condition**	CM	106	29	65
**vCM by Condition**	CM(+SB)	139	34	49
**Proliferative vCM by Condition**	CM + EPC(+SB)	295	154	96
**Proliferative vCM by Condition**	CM + EPC->DC	148	15	37
**Proliferative vCM by Condition**	CM	97	28	58
**Proliferative vCM by Condition**	CM(+SB)	119	46	37

Table S2. Antibody dilution and catalog number.

Target	Dilution	Isotype	Conjugate	Assay	Supplier	Catalog #
αActinin	1:200	Mouse IgG1	None	IHC	Abcam	ab9465
cTnT	1:400	Mouse IgG1	None	IHC/FC	Thermo Fisher	MS-295-P1
cTnT	1:200	Rabbit IgG	None	IHC	Abcam	ab209813
Ki67	1:100	Rabbit IgG	None	FC	Thermo Fisher	MA5-14520
Ki67	1:100	Rabbit IgG	None	IHC	Cell Signaling	9129S
MLC2v	1:500	Rabbit IgG	None	IHC	Abcam	ab79935
MLC2v	1:1000	Rabbit IgG	None	FC	Abcam	ab79935
Mouse IgG	1:400	Goat	AF-488	IHC/FC	Thermo Fisher	A11001
Mouse IgG	1:400	Goat	AF-647	FC	Thermo Fisher	A21235
Nkx2.5	1:200	Mouse IgG1	None	IHC	Santa Cruz Biot.	sc-8697
Oct4	1:250	Rabbit IgG	None	IHC	Thermo Fisher	A24869
Rabbit IgG	1:400	Goat	AF-647	IHC/FC	Thermo Fisher	A21245
RYR2	1:500	Mouse	None	IHS	Novus Biologicals	NB300-543
SERCA2 (ATP2A2)	1:100	Mouse	None	IHC	Invitrogen	MA3919
SSEA4	1:250	Mouse IgG3	None	IHC	Thermo Fisher	A24870
TE7	1:100	Mouse IgG1	None	IHC	EMD Millipore	CBL271
WT1	1:200	Rabbit IgG	None	IHC	Abcam	ab88901

Table S3. q-RT-PCR Primers, related to Figure S7.

Gene	Forward Primer	T_m	Reverse Primer	T_m	Product Size
ATP2A2	CCGGACTTTGAAGGCGTGGATTG	62	CCTCAGCAAGGACTGGTTTTCGG	63	132
EDF1	ATCTTAGCGGCACAGAGACGAG	60	TGTCATGGTGCAGCTCCTCTGT	63	133
GJA1	GGAGATGAGCAGTCTGCCTTTCG	62	ATGAGCCAGGTACAAGAGTGTGG	60	150
KCNJ2	AACAGTGCAGGAGCCGCTTTGT	64	AGGACGAAAGCCAGGCAGAAGA	62	160
MYH6	GGAAGACAAGGTCAACAGCCTGT	61	TCCAGTTTCCGCTTTGCTCGC	63	129
MYH7	GGAGTTCACACGCCTCAAAGAGG	61	TCCTCAGCATCTGCCAGGTTGT	63	147
RYR2	TCTTGAGGTTGGCTTTCTGCCAG	61	CTGTGCCAGCAAAGAGAGGAGCA	62	155
TNNT2	AGAAGAGGAAGCAAAGGAGGCTG	61	AAGTCCACTCTCTCTCCATCGG	59	117

Supplemental Methods

Human induced pluripotent stem cell lines

Three hiPSC-lines were used for these studies. All of the lines were derived from left ventricular cardiac fibroblasts—two lines from female donors, CCND2-hiPSC and F7C6-hiPSC, and one line from a male donor M4C8-hiPSC. The CCND2-hiPSCs were obtained from Jianyi Zhang's lab at the University of Alabama Birmingham and were previously characterized(Zhu et al., 2018). The other lines were reprogrammed in-house using Cyto-Tune 2.0 Sendai Virus reagents following the supplier's manual (Thermo A16517). Sendai virus was confirmed to be depleted via qPCR for two passages. A full karyotype was performed on the lines after Sendai virus depletion was confirmed and pluripotency was seen to be high and the karyotype showed no abnormalities (**Figure S7A-C**). Master banks were created and the STEMCELLS were used within 15 passages of the bank being created.

Human induced pluripotent stem cell maintenance

The hiPSCs were cryopreserved in mFresR^{TM} (STEMCELL 05855). The hiPSCs were maintained in a 6 well plate coated with 8.68 µg/cm^2, or 0.5 mg per well, Cultrex-reduced growth factor (RGF) (R&D Systems 3433-005-01) in 2 mL of mTesR^{TM}1 (STEMCELL 85850) and passaged using ReLesSR^{TM} (STEMCELL 05872) upon reaching 70-80% confluency as follows. Exhausted media was aspirated, and cells were washed with 1 mL of DPBS without calcium or magnesium. Then, 1 mL of room temperature ReLesSR^{TM} was added to each well and incubated for 45 seconds. The ReLesSR^{TM} was aspirated, and the plate was put into the incubator at 37°C for 7 minutes. Warm mTesR^{TM}1 was added to the well and the edges of the plate were gently tapped to release and break the hiPSC colonies from the bottom of the plates. The cell suspension was diluted and transferred into a new 6 well-plate. All cultures were visually inspected for the presence of bacterial and fungal contaminants.

CM differentiation and purification

The hiPSCs were maintained until reaching 70-80% confluency and singularized using Accutase® solution (Millipore Sigma A6964) as follows. Exhausted media was aspirated, and the cells were washed with 1mL of DPBS without calcium or magnesium, and 1mL of room temperature Accutase® solution was added to each well and incubated at 37°C for 8 minutes. The hiPSCs were singularized by pipetting them up and down with a P1000 micropipette and quench using ½ mL of mTesR™1. The cell suspension was transferred to a conical tube, mixed, and counted on a hemocytometer using a 1:1 dilution of 0.4% trypan blue. The hiPSCs were seeded onto 8.68 μg/cm^2 Cultrex-RGF at a density of ~132,000 cells/cm^2, or 0.5x10^6 cells per 12 well, into 1mL of mTesR™1 with 5μM Rock Inhibitor (VWR 103538-728). The exhausted media was replaced with 2mL of fresh mTesR™1 until the cells reached 97-100% confluency (2-4 days). All media changes unless otherwise specified were a volume of 2mL. Upon reaching confluency, defined as day 0 of the differentiation, the media was replaced with RPMI/B27(-ins) (Fisher A1895601) containing 6-8 μM CHIR99021 (Sigma SML1046). The exact CHIR99021 concentration for mesoderm specification depends on the hiPSC-line. After 48 hours, on day 2 of differentiation, the media was replaced with RPMI/B27(-ins) containing 7.5 μM of IWP2 (Tocris 686770-61-6). After another 48 hours, on day 4 of differentiation, the media was replaced with fresh RPMI/B27(-ins). On day 6, and every two days until day 13 of differentiation, the media was replaced with fresh RPMI/B27(+ins) (Thermo 17504001). The timeline is depicted visually in the supplemental materials (**Figure S7D**). To aid in lactate purification, the CM were replated at a 1:2 ratio onto 8.68 μg/cm^2 Cultrex-RGF coated 12 well-plates. Briefly, each well was washed with 1mL of DPBS without calcium or magnesium then 1mL 37°C 0.25% trypsin-EDTA solution was added to each well and incubated at 37°C for 20 minutes. The CM were singularized by pipetting with a P1000 micro pipettor and then quenched with 2mL of RPMI/B27(+ins) with 20% FBS. The cell suspension was transferred to a conical tube and centrifuged at 200g for 5 minutes. The supernatant was aspirated from the cell pellet and the cells were resuspended in RPMI/B27(+ins) containing 10 μM Rock Inhibitor. On day 14, the replated cells are given 1mL of RPMI/B27(+ins). The CM were purified using lactate purification media (DMEM no glucose with 4 mM lactate) (Thermo 11966025, Sigma L7022). On day 15 the media was replaced with 1 mL lactate purification medium. On day 17 the cells were washed with 1mL of DPBS without calcium or magnesium, and the media was replaced

with 1 mL lactate medium. On day 19 the cells were washed with DPBS without calcium or magnesium again and given fresh RPMI/B27(+ins) every three days until replated for 2D co-culture or engineered heart tissue seeding. Differentiation quality controls show cTnT > 95% (**Figure S7E-F**).

Epicardial differentiation

The epicardial differentiation protocol was adapted from a previous publication and visually depicted in the supplement (**Figure S7G**)[36]. The CM differentiation as detailed above is followed until day 6. On day 6, the cardiac progenitor population is replated onto a gelatin-coated 6-well plate at a density of 30,000 cells/cm^2. Briefly, each well was washed with 0.5 mL of DPBS without calcium or magnesium then incubated in 0.5 mL of Accutase® for 8 minutes at 37°C. The cardiac progenitors were singularized using a P1000 micro pipettor and then quenched with 0.5 mL LASR media (DMEM/F12 Advanced, 1.25% GlutaMAX, 60 μg/mL Ascorbic Acid). The cell suspension was transferred into a conical tube and counted using a hemocytometer with a 1:1 dilution of 0.4% trypan blue. The cells were plated onto a gelatin-coated 6-well plate in LASR media with a 5 μM Rock Inhibitor. All media changes were a volume of 2 mL for this differentiation process. On days 7 and 8, the medium is replaced with LASR containing 2-3 μM CHIR99021. The exact CHIR99021 concentration for epicardial specification depends on the hiPSC-line. Starting on day 9 the cells are given fresh LASR media every 2-days until day 12. On day 12 the epicardial cells (EPCs) are passaged or cryopreserved at 1 million cells per vial in epicardial freezing medium (LASR media with 30% FBS, 10% DMSO, 5 μM Rock Inhibitor, 2 μM SB431542). Epicardial cells were used between passages 2 and 5 in these experiments and were all cryopreserved before use. Briefly, the epicardial cells can be thawed onto a gelatin-coated 6-well plate into LASR media with 1% FBS, 5 μM Rock Inhibitor, and 2 μM SB431542. The media was changed with LASR media containing 2 μM SB431542 daily until the cells reached 80-90% confluency. At this point, the cells were passaged or used for a co-culture experiment. The epicardial cells were passaged at a 1:3-1:6 ratio onto a gelatin-coated plate using Versene (Thermo 15040066). Each well was washed with 1 mL of DPBS without calcium or magnesium and then incubated in 1 mL of Versene for 8 minutes at 37°C. The Versene was aspirated and 1 mL of fresh LASR media with 1% FBS and 2 μM SB431542 was added to the

cell layer. The cells are broken into clumps using a P1000 micro pipettor, transferred into a conical tube, diluted, and replated into a new gelatin-coated 6-well plate. The differentiated epicardial cells had a distinct epithelial cobblestone morphology (**Figure S7H**) and were determined to be ≥ 95% pure via flow cytometry for WT1 (Wilms tumor 1) (**Figure S7I**).

Epicardial-derived fibroblast differentiation

The epicardial-derived fibroblast differentiation was adapted from a previous publication and can be visualized in the supplemental materials (**Figure S7J**)[38]. At day 12 of epicardial differentiation the epicardial cells were passaged twice, as detailed above without cryopreservation, and then grown until they reached 100% confluency in LASR media with 2 μM SB431542. At this point, the medium was replaced with LASR containing 10 ng/mL bFGF (R&D Systems 233-FB-010) daily for 10 days. At day 10, the cells were replated at a 1:6 ratio onto tissue culture plastic into fully supplemented FibroGRO (Millipore Sigma SCMF002). To passage, the epicardial-derived fibroblasts (EPD-FBs) were washed, with 1 mL of DPBS without calcium or magnesium then incubated in 1 mL of Accutase® for 20 minutes at 37°C. The cells were broken up using a P1000 micro pipettor and plated into FibroGRO on tissue culture plastic at a 1:6-1:18 ratio. The FibroGRO medium was replaced every 2 days with fresh media until the cells reached 70-90% confluency. The EPD-FBs were used in engineered heart tissue experiments between passage 3 and passage 7. The fibroblasts had an elongated stomal cell morphology, were ≥ 95% pure via flow cytometry for TE7, and had less than 10% remaining WT1-positive cells (**Figure S7K-L**).

Direct co-culture of CM with epicardial cells

CM and EPC co-cultures were seeded in a layered fashion to allow for maximum and consistent CM attachment. First, the plates (12 and 48 well-plates) were coated overnight at 37°C with 17.36 μg/cm^2 of Cultrex-RGF. On day 26 of CM differentiation the CM were washed with 0.5 mL of DPBS without calcium or magnesium and 0.5 mL 37°C 0.25% trypsin-EDTA solution was added to each well and incubated at 37°C for 20 minutes. The CM was singularized by pipetting with a P1000 micro pipettor and then quenched with 1mL of RPMI/B27(+ins) with

20% FBS and 5 μM Rock Inhibitor. The cell suspension was transferred to a conical tube and counted with a hemocytometer with a 1:1 dilution of 0.4% trypan blue. The cells were centrifuged at 200g for 5 minutes and plated at a density of 75,000 cells/cm^2 in LASR media containing 10 μM Rock Inhibitor. On day 27, after the CM had fully attached, the epicardial cells were seeded on top of the CM at a density of 25,000 cells/cm^2 in LASR containing 5 μM Rock Inhibitor (CM + EPC->DC) and 2 μM SB431542 (For the CM + EPC(+SB) condition only). On day 27 the CM-only conditions were given LASR containing 5μM Rock Inhibitor (CM) and 2μM SB431542 (For the CM(+SB) condition only). The media was changed to fresh LASR with or without 2μM SB431542 on day 28 and every 2 days until day 35 of CM differentiation (8 days of co-culture total). To label newly synthesized DNA co-cultures controls that were fated for flow cytometry proliferation assessment were given LASR containing 10 μM 5-ethynyl 2'-deoxyuridine (EdU) for 24 hours before singularization and fixation. On day 35, the CM-only controls and co-cultures were assessed and processed in various ways as detailed below. The 2D co-culture timeline is depicted in **Figure 1A**.

Engineered heart tissue seeding

The EHT negative mold was printed on a Stratasys J750 PolyJet 3D Printer using digital acrylonitrile butadiene styrene (ABS). The ABS negative mold was thoroughly cleaned, oxygen plasma treated for 10 minutes (PDC-32G, Harrick Plasma), and treated with silane vapors for 24-hours in a desiccator vacuum. Sylgard-184 polydimethylsiloxane (PDMS) precursor was mixed at a 1:10 mass ratio with a PDMS curing agent and cast onto the ABS negative mold. The curing PDMS was degassed for 1 hour at room temperature and cured overnight at 50°C. The resultant PDMS mold contains 12 rectangular EHT culture wells and each EHT well contains two posts. The PDMS mold was further cured at 50°C for 1 week. The mold was then cut into six 2-mold squares that were sonicated in 70% ethanol for 30 minutes and autoclaved at 121°C for 25 minutes. The sterile two EHT well PDMS squares were transferred into a sterile petri dish and oxygen plasma was treated for 2 minutes. The PDMS EHT molds were then transferred to a 12-well plate and treated with 0.5 w/v% Pluronic F-127 (Sigma P2443) for 30 minutes at room temperature. At day 24 of CM differentiation the purified hiPSC-CM were dissociated in 0.25% Trypsin-EDTA and combined with EPCs or EPCs and EPD-FBs (CM Tri-culture) that were

singularized in Accutase®. The purity of the CM used in the EHTs were assessed to be ≥95% pure via flow cytometry for cardiac troponin T (cTnT) (**Figure S7F**). The hiPSC-CM and EPCs or EPCs and EPD-FBs were combined at a 3:1 ratio at a density of 10x10^6 cells/mL in a fibrin gel mixture containing 20mg/mL fibrinogen from human plasma (Sigma F3879), LASR with 25 µM Rock Inhibitor and 100U/mL thrombin from human plasma (Sigma 605190) at a 6:3:1 ratio. Each PDMS EHT culture well was made with 100 µL of fibrin gel cell suspension which instantly formed a gel at room temperature upon the addition of thrombin. For the CM-only control conditions, 7.5x10^6 CM/mL of fibrin gel mixture was used so that all conditions contained the same number of CM. Two EHTs of the same condition were cultured per well of a 12-well plate. The EHTs were cultured in LASR with 10 µM Rock Inhibitor, 20 µg/mL aprotinin (Sigma 10981532001), and (+/-) 2 µM SB431542 for 24 hours after seeding. The EHTs were then given fresh LASR media with 20 µg/mL aprotinin (+/-) 2µM SB431542 every two days until assessment at 29-31 days after EHT seeding. The EHTs fated for immunohistochemical (IHC) analysis were treated with 10 µM EdU for 24 hours before fixation and processing as detailed below.

Calcium transients of direct co-cultures, transwell co-cultures and EHTs

On day 35, the 2D co-cultures and controls that were plated into 12 well-plates were incubated with 5 µM Rhod-2AM calcium-sensitive dye in LASR medium for 30 minutes at 37°C. For the EHTs calcium was assessed 29-31 days after EHT seeding. The EHTs were kept in the PDMS mold and incubated with 5µM Rhod-2AM calcium-sensitive dye in LASR medium for 30 minutes at 37°C. The dye was aspirated and replaced with Tyrode's Salt Solutions with 1 g/L sodium bicarbonate (TSS) (Sigma Aldrich T2145). The 2D cells or EHTs were equilibrated in TSS for 30 minutes at 37°C and then imaged on the TxRed channel with a 30-millisecond exposure and gain of 1.0 at 10X magnification on a fluorescent microscope (Leica Microsystems). Three 20-second videos per well or EHT were taken, with n=3 wells per condition or n=3-4 EHTs per condition for each replicate. Videos were taken at a frame rate of 7 frames/s. The co-cultures and EHTs were paced using a platinum electrode at 1 Hz with a voltage of 7 V and pulse duration of 0.02- milliseconds. The calcium transient data acquired was

processed in ImageJ by importing the images and converting them into image stacks. Three regions of interest within each video were selected and the Z-axis profile of fluorescent intensity versus time was exported as a text file. The text files were analyzed using an in-house MATLAB code to determine the inter-spike-interval (s) of the calcium peaks, the time to peak (s), the maximum amplitude (F/F_0) of the calcium peak as well as the average upstroke and downstroke velocities ($F/F_0/s$). Any videos acquired in which the inter-spike-interval was less than 0.95 or greater than 1.05 were excluded due to insufficient pacing.

Patch clamp of CM

Patch clamp was done for the CM + EPC(+SB) and the CM(+SB) control only. The direct 2D co-cultures were replated at a density of ~10,000 cells/cm^2 onto 35 mm TC-treated petri dishes coated with 17.36 μg/cm^2 Cultrex-RGF. After 24 hours patch clamp measurements of the replated CM were taken. The patch clamp and AP recording method is described in a previous publication(Xie et al., 2022). In brief, an Axopatch-200B amplifier (Molecular Devices, Foster City, USA) was used to record APs by a ruptured whole-cell current-clamp technique. For AP recordings, pipettes were filled with (in mmol/L) 120 potassium gluconate, 20 KCl, 5 NaCl, 5 HEPES, 0.02 EGTA, 0.05 $CaCl_2$, and 5 MgATP (pH 7.2, $[Ca2^{2+}]_i \approx 100$ nmol/L). The extracellular bathing solution contained (in mmol/L) 140 NaCl, 5.4 KCl, 1 $MgCl_2$, 10 HEPES, 1.8 $CaCl_2$, and 5.5 glucose (pH 7.4). Pipette resistances were ~3 MΩ. The recordings were low pass filtered at 10 kHz and digitized at 20 kHz.

Traction force microscopy of 2D co-culture CM

Traction force microscopy (TFM) was executed by the construction and micropatterning of polyacrylamide gel constructs as previously described(Garay et al., 2022; Hald et al., 2016; Rothermel et al., 2022; Win et al., 2017). Briefly, stamps were made from PDMS from a master silicon wafer that was fabricated using photolithography. The stamps are rectangular arrays containing ~20 μm x 140 μm features used to micropattern islands that can be coated with protein for cell adhesion. For sterilization, the stamps were sonicated with 70% ethanol for 30 minutes then coated with ~123 μg of Cultrex-RGF per substrate diluted in DMEM/F12 with

HEPES and left at 37°C overnight. Next, 15 mm glass coverslips were rinsed with acetone and ethanol and then plasma treated for proper cleaning. The Cultrex-RGF stamps were then placed feature side down on the plasma-treated coverslips. The polyacrylamide gel was created and bound to a 25 mm glass coverslip as follows. The 25 mm glass coverslip was cleaned and treated with UV for 8 minutes. The glass was functionalized with 3% silane solution for 5 minutes. The prepolymer polyacrylamide gel solution was composed of 10/0.13% w/v acrylamide/bisacrylamide that was doped with 2% v/v 0.2 μm diameter red fluorescent microspheres. After the solution was degassed, 0.002/0.05% w/v initiators tetramethylethylenediamine/ammonium persulfate, 0.005% w/v N-hydroxysuccinimide ester, and 0.014% 1 M HCl were added to the gel. Then 10 μL of gel solution was pipetted onto the center of the silane-treated glass coverslip. The Cultrex-RGF coated coverslips were placed pattern side down onto the polyacrylamide gel solution and polymerized at room temperature for 1 hour. The polyacrylamide gel was rehydrated and the Cultrex-RGF coverslip was removed. The Young's modulus of the polymerized polyacrylamide gel was previously determined to be 13.5 kPa(Hald et al., 2016). At day 35 of the 2D direct co-culture experiments the co-cultures were singularized using 0.25% trypsin-EDTA as previously described and plated at a density of 35,000 cells per micropatterned polyacrylamide gel substrate into LASR medium containing 10 μM Rock Inhibitor. The following day the cells were given fresh LASR media. After 48 hours of recovery on the substrates, TFM measurements were acquired. Videos were taken on an inverted microscope in a CO_2 controlled chamber at 37°C. CM beat rate was controlled using 1 Hz electrical stimulation with a 40 ms bipolar pulse with a MyoPacer external field stimulator (IonOptix). Only micropatterned hiPSC-CM that were paced were imaged for these studies. Both brightfield and fluorescent images were acquired of the micropatterned hiPSC-CM at a frame rate of 30-100 frames per second. The images were taken for 3 seconds so that three consecutive contractions could be imaged in each video. Following image acquisition, the cells were lysed using sodium dodecyl sulfate, and cell free images of the fluorescent bead layer were acquired. The TFM measurements were analyzed in ImageJ as previously described(Garay et al., 2022; Hald et al., 2016; Rothermel et al., 2022; Win et al., 2017). The cell-induced bead displacement between the cell-attached and cell-free images at each time point was calculated using a particle image velocimetry algorithm in ImageJ. To generate a field of substrate traction stress vectors, an unconstrained Fourier-transform traction cytometry algorithm(Butler et al., 2002) was applied

to the field of cell-induced bead displacements with a regularization factor of 1×10^{-9} and a Poisson's ratio of 0.5. Substrate traction stress vectors acting on point n are defined by $\boldsymbol{T^n} = T_x^n \boldsymbol{e_x} + T_y^n \boldsymbol{e_y}$, where T_i^n is the traction stress acting in the ith direction ($i = x, y$), and $\boldsymbol{e_i}$ is the unit vector in the ith direction. Substrate traction forces acting at point n were taken as the substrate traction stress vector $\boldsymbol{T^n}$ multiplied by the area a^n over which $\boldsymbol{T^n}$ acts. At the cell-gel interface, substrate traction forces are balanced by internal cell forces $\boldsymbol{f^n}$ such that $\boldsymbol{f^n} + \boldsymbol{T^n} a^n = 0$. Substrate traction forces in turn are defined as $\boldsymbol{f^n} = f_x^n \boldsymbol{e_x} + f_y^n \boldsymbol{e_y}$, where f_i^n is the internal cell force acting on point n in the ith direction. The total internal cell force f_i acting in direction i was summed around the midline of the cell, such that $2f_i = \sum_n {f_i^n r_i^n}/{|r_i^n|}$, where r_i^n is the distance in the ith direction between the cell midline and the location n of the traction force. The force of contraction was defined as the difference in internal cell force between the cell at its maximum contraction and the uncontracted state. The contraction force was calculated for three consecutive contractions of the cell and then averaged.

Optical Mapping of EHTs

The EHT device underwent a 20-minute immersion in a 10 µM voltage-sensitive dye di-4-ANEPPS solution prepared in TSS. Subsequently, the dye mixture was replaced with fresh TSS. After a stabilization period of 2-5 minutes, EHT were excited using two continuous-excitation green lasers (532 nm, 1 W; Shanghai Dream Lasers Technology, Shanghai, China). Fluorescence intensity was captured for 10 seconds using high-resolution cameras (14-bit, 80 × 80-pixel, Little Joe, RedShirt Imaging, SciMeasure, Decatur, GA) at a rate of 500 frames per second(Garay et al., 2022; Hald et al., 2016; Rothermel et al., 2022; Win et al., 2017). Pacing at 1 Hz was conducted through a bipolar electrode, and optical mapping recordings were taken during this pacing and filtering using a bandpass filter 3-30 Hz. The duration of the optical APs (APD) was assessed at 80% repolarization. From this 2D APD maps were generated to illustrate the spatial dispersion in APD. We also quantified activation times (AT) at the peak of the derivative of voltage over time (dV/dt max), and calculated local conduction velocity (CV) as described previously(Kupfer et al., 2020; Lin et al., 2022).

Twitch force and stress measurements of EHTs

After 29-31 days in culture the EHTs were taken off the PDMS posts and mounted at their initial length on needles on the Mach-1 Micromechanical Tissue Tester (Biomomentum) using a 10g force transducer. The EHTs were paced using a platinum electrode at 1 Hz with a voltage of 7-9 V and pulse duration of 0.02s. The force measurements were acquired for 20s at strains of 0, 5, 10, 15, and 20%. Using MATLAB a low pass frequency filter was used to remove noise from force data and the average twitch force was calculated. The maximum force for each EHT at any given strain was reported as the maximum twitch force. Cryosections of the EHTs were used to determine the average cross-sectional area and the twitch stress was calculated by dividing the average twitch force by the average cross-sectional area of the cryosections (**Figure S8A-B**).

Murine E10, E12, and E17 heart harvesting, sectioning, and staining

All mouse experiments were performed using the outbred CD-1 mouse line. Upon mating, the detection of a mucus plug was considered day 1 of embryonic development. At the indicated time points, pregnant dams were euthanized, and embryos were extracted from the uterus. For the embryonic day (E) 10 and E12 whole embryos were used, while for E17 samples the heart was first excised from the embryo. For E17, hearts were immediately embedded in Tissue-Tek O.C.T. For E10 and E12 samples, embryos were fixed in fresh 4% paraformaldehyde (PFA) in PBS for 1-1.5 hours in a 12-well plate at room temperature, followed by a 3x15min wash in PBS. Afterward, embryos were placed in a 5% sucrose in PBS solution at 4°C until the tissue sunk to the bottom of the plate, followed by 10%, 15%, and finally 20% sucrose solution. E10 and E12 embryos were embedded in a 7.5% gelatin in a sucrose solution. Tissue blocks were sectioned into 4μm slices. E17 samples were fixed in 4% PFA solution for 10 minutes at room temperature, followed by two washes for 5 minutes in PBS and then directed to permeabilization. For E10 and E12 samples, prewarmed PBS was used to wash off gelatin. Slides were permeabilized in 2% BSA, 0.5% Tween-20 in PBS for 10-15 minutes and blocked in 2% BSA, 0.1% Tween-20 for 1 hour both at room temperature. Slides were incubated overnight at 4°C in a blocking solution with primary antibodies of nkx2.5, alpha-actinin, Ki67, and Wheat Germ Agglutinin (W11261, Invitrogen, 1:200 dilution). Antibody catalog number and dilution in **Table**

S2. After four times washing for 2 minutes in PBS, slides were incubated in secondary antibodies at room temperature at 1:400 dilution and DAPI for 1 hour. The images were taken with a Zeiss upright microscope equipped with an AxioCam MRm camera, using the 40x and 20x objectives, and processed using Fiji software. CM size was quantified using Fiji by tracing the area of individual CM. Around 120 CM sizes were measured for each time point. The percentage of proliferative ventricular CM was quantified using Cellprofiler. Sarcomere lengths were measured using the Fiji plugin SarcOptiM. At least 100 CM sarcomeres were measured for E12 and E17 time points, and 90 were measured for E10 samples.

Flow cytometry for CM proliferation, phenotype, and differentiation quality control

For proliferation assessment, the co-cultures were treated with EdU for 24 hours before harvesting on day 35. For co-culture proliferation and ventricular phenotype assessment, the co-cultures were singularized using 0.25% Trypsin-EDTA for 20-minutes at 37°C. Quality control samples of day 26 CM, EPD-FBs, and aLVCF were all singularized using the methods describes above. Once singularized, the cells were spun down at 200g for 5 minutes, the supernatant was poured off and the cells were resuspended in 1% PFA in PBS for 20 minutes. After 20 minutes the cells were spun down at 200g for 5 minutes, the fixative was removed, and the cells were resuspended in -20°C 90% v/v Methanol. The samples were stored in the freezer at -20°C until staining for flow cytometry. For staining, the cells were strained with a 70 µm cell strainer (Corning 352350) to remove any large clumps. ~300,000-1,000,000 cells per sample were separated into individual conical tubes and the methanol was washed off the cells by adding 2 mL of flow buffer (PBS with 5% BSA and 0.2% Triton-X-100) spinning down the cells at 200g for 5 minutes, pouring off the supernatant, then repeating this process once more. After the second wash, a P200 micropipette was used to remove as much residual flow buffer from the pellet as possible, and then the cells were either resuspended in 100 µL of Click-It EdU reaction mixture with Cy5-Azide for 30 minutes at room temperature (for proliferation assay only) or 100 µL of primary antibody dilution for 1hr at room temperature. For the proliferation assay, after the Click-It reaction, the cells were washed twice with 2mL of flow buffer as described above and then resuspended in 100 µL of primary antibody dilution for 1hr at room temperature. Following the primary antibody incubation, the samples were washed twice with flow buffer as described

above and then resuspended in 100 μL of secondary antibody solution. The cells were incubated at room temperature for 30 minutes and then washed twice with flow buffer. Following staining the cells were resuspended in ~300 μL of flow buffer and data was acquired for 10,000 events within the defined cell population using a BD Accuri C6 flow cytometer. The antibody dilutions and catalog number used for flow cytometry can be found in **Table S2**.

CM morphology assessment

At the endpoint of co-cultures, day 35, the 2D co-cultured were using 0.25% Trypsin-EDTA for 20 minutes at 37°C and replated onto 17.36 µg/mL Cultrex-RGF coated glass chamber slides at a density of ~10,000 cells/cm^2. After 24 hours the cells were fixed in diastole using 4% paraformaldehyde in PBS with 90mM KCl, 4mM EDTA, and 4mM $MgCl_2$ for diastolic arrest of the CM. The chamber slides were stored in PBS with 0.1% sodium azide at 4°C until staining. To stain, samples were permeabilized using 0.2% Triton-X-100 for 1 hour, blocked in BGST (50g/L BSA, 10g/L glycine, 2% Goat Serum, 0.1% Triton-X-100) for 2 hours and then incubated with anti-αActinin antibody for 3 hours s at room temperature. The primary antibody was washed off and the cells were incubated with AF-488 Goat anti-mouse secondary antibody for 1 hour. The antibody dilutions and catalog number can be found in **Table S2**. The sample was washed with PBS and the chamber attachment was removed from the slide and mounted with DAPI DABCO mounting medium (90% Glycerol, 25mg/mL DABCO, 2.5ng/mL DAPI in 1X PBS, pH 8.6) mounting medium and sealed with a coverslip. The slides were imaged using a 40X oil immersion lens on the FITC and DAPI channels. Five fields of view were imaged in each well, for each condition three wells were imaged. Data was collected in this fashion for three replicates for each of the M4C8, F7C6, and CCND2-hiPSC lines. The collected data was de-identified and given to a researcher for blinded analysis of CM a) sarcomere length using SarcOptiM ImageJ plugin(Pasqualin et al., 2016) b) cell perimeter c) cell area d) aspect ratio e) nucleation. The aspect ratio was defined as the longest axis of the cell that intersects the nucleus (the long axis) divided by the longest distance that is orthogonal to the long axis and intersects the nucleus (the short axis).

Immunocytochemistry and immunohistochemistry of direct co-cultures and EHTs

2D co-cultures seeded in 48-well plates and EHTs were preserved in 4% paraformaldehyde in PBS with 90mM KCl, 4mM EDTA, and 4mM $MgCl_2$ for diastolic arrest of the CM(Yücel et al., 2020). Briefly, the cells or EHTs were washed with DPBS without calcium or magnesium and then incubated for either 15 minutes (2D) or 45 minutes (EHTs) at room temperature followed by washing with PBS twice before storing in PBS with 0.1% sodium azide at 4°C until staining (2D) or cryo-embedding (EHTs). To process EHTs for cryo-embedding the fixed tissue was dehydrated in 30% sucrose for 48 hours. After dehydration the EHTs were cut in half and transferred into 50:50 30% Sucrose:OCT for 30 minutes then transferred into cryo-molds containing OCT and frozen in the -80°C until cryo-sectioning. Using a cryostat (Leica CM1900) the EHTs were sectioned across the center to determine the cross-sectional area as well as transversely at a 5-10 μm thickness. For staining, both the 2D wells and EHT sections were permeabilized using 0.2% Triton-X-100 for 1 hour and blocked in BGST for 3 hours before an overnight incubation at 4°C with the primary antibody dilutions in BGST. After the overnight incubation, the samples were washed twice with 0.2% Tween-20 and then twice with PBS. The samples were then incubated with secondary antibody dilution in BGST for 1.5 hours and then washed twice with 0.2% Tween-20 and with PBS. The samples were incubated with 1g/L DAPI in PBS for 20 minutes and washed with PBS 3 times. The EHT sections were mounted in Prolong™ Glass Antifade Mountant (Thermo P36982). The 2D wells were imaged at 20X magnification on a fluorescent microscope (Leica) and the EHT sections were imaged on an Olympus FluoView IX2 Inverted Confocal microscope with a 60X oil immersion lens. Phalloidin and DAPI images for tissue alignment as well as brightfield and phalloidin images for EHT cross-sectional area measurements were imaged at 20X magnification on a fluorescent microscope (Leica) using the ImageJ OrientationJ plugin (**Figure S8C-F**). The antibody dilutions and catalog number used for IHC can be found in **Table S2**.

Transmission electron microscopy of co-cultured CM

The 2D co-culture of conditions CM + EPC(+SB) and CM(+SB) were seeded as described above on 13 mm Thermonox® (Nalge Nunc International) plastic coverslips coated with 17.36 $\mu g/cm^2$ Cultrex-RGF and cultured for 9 days. The cells on the coverslips were fixed in 3%

paraformaldehyde and 1.5% glutaraldehyde in 0.1M sodium cacodylate buffer with 2.5% sucrose, 5mM calcium chloride, and 5mM magnesium chloride (pH 7.4) for 1-2 hr at room temperature (RT) after 8-days of co-culture. Cell cultures were rinsed in buffer (3x, 5 min ea), fixed in 1% OsO4 in 0.1M sodium cacodylate buffer for 1 hr at RT, rinsed in ultrapure water (NANOpure Infinity®; Barnstead/Thermo Fisher Scientific; Waltham, Maryland) (3x, 5 min ea), en bloc stained with 1% aqueous uranyl acetate for 1 hour at RT, and rinsed in ultrapure water (3x, 5 min ea). Samples were then dehydrated in an ethanol series (50%, 75%, 95% (all 2x), 100% (3x), 5 min ea) and infiltrated with Embed 812 resin (Electron Microscopy Sciences, Hatfield, Pennsylvania) (1:1 ethanol:resin, 2 hrs, 100% resin with catalyst, 2 hrs, 2x). Cultures were embedded by placing the coverslips upside-down on top of resin-filled capsule molds, then polymerized in a 60C oven for 48 hours. Ultrathin sections 80–100 nm thick were cut on a Leica Ultracut UCT microtome using a diamond knife and collected on formvar/carbon-coated copper slot (2mm x 1mm) grids (Electron Microscopy Sciences, Hatfield, Pennsylvania). They were stained with 3% aqueous uranyl acetate for 20 min, rinsed in ultrapure water (15 sec, 5x), stained with Sato's triple-lead stain (Sato 1968) for 3 min, and rinsed in ultrapure water (15 sec, 5x). Sections were examined with a JEOL JEM1400-Plus transmission electron microscope operating at 60 kV. Images were recorded with an Advanced Microscopy Techniques XR16 camera using AMT Capture Engine software ver. 7.0.0.187.

Single-cell RNA sequencing Sample Preparation

Single-cell RNA sequencing was done following the supplier's manual for the Chromium Next GEM Single Cell Sequencing Kits. The 2D co-cultures were singularized using 0.25% trypsin-EDTA for 20 minutes at 37°C as described above. The viability of the cells was determined via propidium iodide on the BD Accuri C6 flow analyzer and then the cells were prepped for fixed single-cell sequencing according to the Chromium Next GEM Single Cell Fixed RNA Sample Preparation Kit (10X Genomics 1000414). Libraries for single-cell sequencing were created using the Chromium Fixed RNA Kit, Human Transcriptome, 4 rxns x 4 BC (10X Genomics 1000475) by following the manual for probe hybridization and library creation. The samples were sequenced according to the 10X genomics FLEX single-cell sequencing workflow at the University of Minnesota Genomics Center (UMGC). Briefly, pooled libraries were denatured

and diluted to the appropriate clustering concentration. The libraries were then loaded onto the NovaSeq paired end flow cell and clustering occurred on board the instrument. Once clustering was completed, the sequencing reaction immediately began using the Illumina 2-color SBS chemistry. Upon completion of read 1, 2 separate 8 or 10 base pair index reads were performed. Finally, the clustered library fragments were re-synthesized in the reverse direction thus producing the template for paired end read 2.

Single-cell RNA sequencing analysis

Reads were aligned to the human reference genome provided by 10X Genomics (refdata-gex-GRCh38-2020-A) using cellRanger (v 7.1.0) and the Chromium_Human_Transcriptome_Probe_Set_v1.0_GRCh38-2020-A. Analysis was. Performed using R (v 4.1.0). Libraries were quality-controlled individually as follows: 1) The matrix of filtered counts per cell barcode was analyzed using Seurat (v 4.3.0.1). 2) Empty droplets were filtered out with a minimum total RNA and Feature counts per barcode based on their count distribution on a per-sample basis. 3) The presence of multiplets was assessed using the scDblFinder (v 1.8.0) package, followed by manual filtering of multiplets of the same cell type using an upper RNA count limit. 4) Gene expression was estimated on the clean data using the transformed counts (SCTransform) with the percent of mitochondrial reads as covariate.
To take full advantage of replication while avoiding confounding batch effects, biological replicates of the same condition were integrated into a single object using Seurat’s standard workflow. Then, integrated treatment objects were merged into a single object and renormalized (SCTransform). This combined integration/modeling approach yielded cell type proportions that correlated with the expected experimental outcomes based on the seeded cell types, while the default integration or covariate batch modeling only did not. Principal component (PC) analysis of the combined object was performed for dimensionality reduction. UMAP reductions were used only for visualization purposes. Cell classification was performed using the FindNeighbors and FindClusters functions (Seurat) based on the first 25 PCs and the normalized counts. Cell cluster identification was performed based on their relative expression of genes of interest for the CM and EPC populations, their marker genes, and cluster abundance per treatment. Differential gene expression tests were done with the default parameters (test.use = "wilcox”).

Overrepresentation Analysis (ORA) was performed on all sets of differentially expressed genes (DEGs) using the clusterProfiler (v 4.2.2) package and the gene ontology and KEGG databases in the msigdbr (v 7.5.1) package. Term and gene set databases were filtered to contain only genes detected across all treatments in the experiment (14263 genes). In detail, DEGs for each identity class were separated into up or down-regulated lists, the maximum between all or the top 300 of each list, ranked by the average log2 fold change, was tested with the enricher function using p-value and q-value cutoffs of 0.05 and the Benjamini–Hochberg correction for multiple hypothesis testing. Each of the three subcategories of the GO terms (BP, MF, and CC) and the KEGG database were tested separately.

qPCR for maturation markers

The EHTs from different groups were lysed using 600ul of RLT-lysis buffer containing β-mercaptoethanol (RNeasy kit (Qiagen)). Subsequently, the lysate was further homogenized using 21G and 25G needles (~10 times each) for complete cell lysis. Total RNA was isolated using the RNeasy kit according to the manufacturer's protocol, followed by on-column DNA digestion using DNase1 to remove any traces of DNA as per the instructions. The elution was performed using pre-heated (80C) DNase/RNase-free water. To obtain a higher yield, we perform a second round of elution using the eluate. The quantity and quality were assessed by measuring absorbance at 260nm and 280nm using a microplate reader instrument. For cDNA synthesis, 250 ng of total RNA was used and synthesized using the SuperScript IV VILO kit (Thermo Fisher Scientific) according to the manufacturer's protocol in a 20ul reaction volume. The reaction mix was diluted 10 times using DNase/RNase-free water for the next steps. Quantitative PCR (qPCR) was performed using gene-specific oligos and the SYBR-green method. The list of oligos used is provided in **Table S3**. For the qPCR analysis, each transcript was normalized to the housekeeping gene, *EDF1*. To visualize CM-specific changes, we further normalized the transcript levels to the levels of *TNNT2*.

Supplemental References

Butler, J. P., Tolić-Nørrelykke, I. M., Fabry, B., & Fredberg, J. J. (2002). Traction fields, moments, and strain energy that cells exert on their surroundings. *American Journal of Physiology-Cell Physiology*, *282*(3), C595–C605. https://doi.org/10.1152/ajpcell.00270.2001

Garay, B. I., Givens, S., Abreu, P., Liu, M., Yücel, D., Baik, J., Stanis, N., Rothermel, T. M., Magli, A., Abrahante, J. E., Goloviznina, N. A., Soliman, H. A. N., Dhoke, N. R., Kyba, M., Alford, P. W., Dudley, S. C., Van Berlo, J. H., Ogle, B., & Perlingeiro, R. R. C. (2022). Dual inhibition of MAPK and PI3K/AKT pathways enhances maturation of human iPSC-derived cardiomyocytes. *Stem Cell Reports*, *17*(9), 2005–2022. https://doi.org/10.1016/j.stemcr.2022.07.003

Hald, E. S., Timm, C. D., & Alford, P. W. (2016). Amyloid Beta Influences Vascular Smooth Muscle Contractility and Mechanoadaptation. *Journal of Biomechanical Engineering*, *138*(11), 111007. https://doi.org/10.1115/1.4034560

Kupfer, M. E., Lin, W.-H., Ravikumar, V., Qiu, K., Wang, L., Gao, L., Bhuiyan, D. B., Lenz, M., Ai, J., Mahutga, R. R., Townsend, D., Zhang, J., McAlpine, M. C., Tolkacheva, E. G., & Ogle, B. M. (2020). In Situ Expansion, Differentiation, and Electromechanical Coupling of Human Cardiac Muscle in a 3D Bioprinted, Chambered Organoid. *Circulation Research*, *127*(2), 207–224. https://doi.org/10.1161/CIRCRESAHA.119.316155

Lin, W.-H., Zhu, Z., Ravikumar, V., Sharma, V., Tolkacheva, E. G., McAlpine, M. C., & Ogle, B. M. (2022). A Bionic Testbed for Cardiac Ablation Tools. *International Journal of Molecular Sciences*, *23*(22), 14444. https://doi.org/10.3390/ijms232214444

Pasqualin, C., Gannier, F., Yu, A., Malécot, C. O., Bredeloux, P., & Maupoil, V. (2016). SarcOptiM for ImageJ: High-frequency online sarcomere length computing on stimulated cardiomyocytes. *American Journal of Physiology-Cell Physiology*, *311*(2), C277–C283. https://doi.org/10.1152/ajpcell.00094.2016

Rothermel, T. M., Cook, B. L., & Alford, P. W. (2022). Cellular Microbiaxial Stretching Assay for Measurement and Characterization of the Anisotropic Mechanical Properties of Micropatterned Cells. *Current Protocols*, *2*(2), e370. https://doi.org/10.1002/cpz1.370

Win, Z., Buksa, J. M., Steucke, K. E., Gant Luxton, G. W., Barocas, V. H., & Alford, P. W. (2017). Cellular Microbiaxial Stretching to Measure a Single-Cell Strain Energy Density Function. *Journal of Biomechanical Engineering*, *139*(7), 071006. https://doi.org/10.1115/1.4036440

Xie, A., Liu, H., Kang, G.-J., Feng, F., & Dudley, S. C. (2022). Reduced sarcoplasmic reticulum Ca2+ pump activity is antiarrhythmic in ischemic cardiomyopathy. *Heart Rhythm*, *19*(12), 2107–2114. https://doi.org/10.1016/j.hrthm.2022.08.022

Yücel, D., Solinsky, J., & Van Berlo, J. H. (2020). Isolation of Cardiomyocytes from Fixed Hearts for Immunocytochemistry and Ploidy Analysis. *Journal of Visualized Experiments*, *164*, 60938. https://doi.org/10.3791/60938

Zhu, W., Zhao, M., Mattapally, S., Chen, S., & Zhang, J. (2018). CCND2 Overexpression Enhances the Regenerative Potency of Human Induced Pluripotent Stem Cell–Derived Cardiomyocytes: Remuscularization of Injured Ventricle. *Circulation Research*, *122*(1), 88–96. https://doi.org/10.1161/CIRCRESAHA.117.311504
