# Supplementary material for: Developmental cues from epicardial cells simultaneously promote cardiomyocyte proliferation and electrochemical maturation
Source: Stem Cell Reports. 2025 Jul 3;20(8):102572. doi: 10.1016/j.stemcr.2025.102572 (PMC12365841; doi:10.1016/j.stemcr.2025.102572)
Supplement: Document S2. Article plus supplemental information [file mmc7.pdf]

Stem Cell Reports
Article
OPEN ACCESS

Developmental cues from epicardial cells simultaneously promote cardiomyocyte proliferation and electrochemical maturation

Sophie E. Givens,[1] Abygail A. Andebrhan,[1] Ruchen Wang,[2] Xiangzhen Kong,[1] Taylor M. Rothermel,[1] Sanaz Hosseini,[3] An Xie,[2] Mohammad Shameem,[1,7] Andrea A. Torniainen,[2] Somayeh Ebrahimi-Barough,[1] Samuel F. Boland,[1] Maya Johnson,[1] Natalia Calixto Mancipe,[8] Bhairab N. Singh,[1,4,7] Samuel Dudley,[2] Patrick W. Alford,[1] Elena G. Tolkacheva,[1,2,3,6] Jop H. van Berlo,[2,4] and Brenda M. Ogle[1,4,5,6,9,*]
[1]Biomedical Engineering, University of Minnesota, Minneapolis, MN, USA
[2]Lillehei Heart Institute (LHI), Department of Medicine, University of Minnesota, Minneapolis, MN, USA
[3]Electrical Engineering, University of Minnesota, Minneapolis, MN, USA
[4]Stem Cell Institute, University of Minnesota, Minneapolis, MN, USA
[5]Department of Pediatrics, University of Minnesota, Minneapolis, MN, USA
[6]Institute of Engineering in Medicine, University of Minnesota, Minneapolis, MN, USA
[7]Department of Rehabilitation Medicine, University of Minnesota, Minneapolis, MN, USA
[8]Minnesota Supercomputing Institute, University of Minnesota, Minneapolis, MN, USA
[9]Lead contact
*Correspondence: ogle@umn.edu
https://doi.org/10.1016/j.stemcr.2025.102572

SUMMARY

Accumulating evidence indicates that maturation limits cardiomyocyte proliferation. We expand on that theory by co-culturing human induced pluripotent stem cell (hiPSC)-cardiomyocytes (CM) with epicardial cells (EPCs) and epicardial-derived cells in both 2D co-cultures and 3D engineered heart tissues (EHTs). In 2D co-cultures, the percentage of proliferating CM increased in parallel with stark electrophysiologic improvements. Single-cell transcriptomics revealed a significant shift in the bulk CM population of the epicardial-CM co-cultures as characterized by more fetal-like myofilament isoforms but with enhanced pathways associated with electrochemical maturation. The 3D-EHTs containing EPCs showed more limited proliferation but a similar improvement in CM electrophysiologic function. Next, epicardial-derived fibroblasts (EPD-FBs) were added to the EHTs containing EPCs, and we observed significant myofilament maturation and increased force generation. Our results suggest that some aspects of CM maturation (i.e., electrochemical) can occur when proliferation rates are relatively high, and that sarcomere-associated mechanical maturation occurs at later developmental stages when proliferation has largely ceased.

INTRODUCTION

The use of human induced pluripotent stem cell (hiPSC)-derived cardiac cells to study health and disease has exploded in the last decade with the advent of robust and reproducible methods for the differentiation of CM (Lian et al., 2012). Unfortunately, CM generated from such protocols are largely immature (Guo and Pu, 2020). This is evident by their small and rounded morphology, sarcomere disorganization, automaticity, the primary use of glycolysis for adenosine triphosphate (ATP) production, the expression of fetal myofibril isoforms, a fetal ion channel composition and lack of expression of major calcium handling proteins of the sarcoplasmic reticulum and T-tubule (Guo and Pu, 2020). Copious efforts to drive hiPSC-CM maturation *in vitro* have been attempted over the last decade with various levels of success. Approaches to drive maturation include the use of 3D culture systems that more accurately mimic the complex milieu of signals CM received *in vivo* such as organoids, engineered heart tissues (EHTs), and 3D printed chambered structures (Kupfer et al., 2020; Lemoine et al., 2017; Mills et al., 2017). The switch from 2D to 3D culture alone promotes some level of CM maturation (Branco et al., 2019; Correia et al., 2018; Ergir et al., 2022). Further, in EHTs, the imposition of increased mechanical "afterload" enhances force generation and maturation (Leonard et al., 2018). Additionally, some more user-friendly techniques exist including the use of "maturation media" to impose a switch from glucose to fatty acid-based metabolism and treatment with small molecule mitogen-activated protein kinase (MAPK) inhibitors that mimic the downregulation of the MAPK pathway seen in human ventricular tissues during development (Garay et al., 2022; Horikoshi et al., 2019). Finally, the co-culture of CM with other cell types present in the developing heart such as cardiac fibroblasts, endothelial cells, and epicardial-derived cells can enhance hiPSC-CM maturation (Bargehr et al., 2019; Beauchamp et al., 2020; Dunn et al., 2019; Giacomelli et al., 2020).

Most of these methods impose changes characteristic of post-natal heart development. The stark changes in afterload and preload caused by birth that result in developmental cardiac hypertrophy, the electrical stimulation present from the development of the cardiac pacemaker system, the switch to fatty acid metabolism, and the inhibition of pathways such as MAPK do not occur until postnatal development (Marchianò et al., 2019; Salameh et al., 2023). Freshly differentiated hiPSC-CM resemble early fetal

Published by Elsevier Inc. on behalf of International Society for Stem Cell Research.
This is an open access article under the CC BY license (http://creativecommons.org/licenses/by/4.0/).

CM that are largely undefined in patterning that takes place during embryonic development; during embryonic development, atrial, ventricular, compact, and trabecular myocardium show distinct, spatial, genetic, and functional patterning (Lai et al., 2010; Tian et al., 2017; Vicente-Steijn et al., 2015). Therefore, it might be advantageous to first mimic embryonic development before the implementation of post-natal conditions. This study explores the role of epicardial and epicardial-derived cells on hiPSC-CM and their ability to promote hiPSC-CM maturation by mimicking embryonic heart development.

The epicardium originates during fetal heart development in the proepicardial organ by E9.5 and migrates to cover the primary heart tube by E10.5-E12 in mice (Rudat and Kispert, 2012; Vicente-Steijn et al., 2015). Epicardial cells (EPCs) serve three major functions during embryonic development: (1) secretion of key growth factors that drive CM proliferation and formation of the compact myocardium (T. H.-P. Chen et al., 2002; Li et al., 2011); (2) the main source of interstitial fibroblasts in the myocardium (Acharya et al., 2012); and (3) the production of vascular smooth muscle cells that are responsible for driving coronary vascular formation (Grieskamp et al., 2011). The epicardium is so important for proper cardiac development that murine models with deficiencies in the epicardium have thin ventricular walls, impaired coronary artery angiogenesis, and embryonic or immediate perinatal lethality (Wu et al., 2013; Zamora et al., 2007). These findings indicate that the epicardium is essential for proper ventricular morphogenesis. Thus, this study explores the impact of epicardial and epicardial-derived cells on hiPSC-CM maturation by mimicking *embryonic* heart development.

Recently, protocols to differentiate and maintain EPCs using small molecule transforming growth factor β (TGF-β) inhibitors have been described (Bao et al., 2017). So far, only a few studies have looked at the effect of hiPSC-derived epicardial and epicardial-derived cells on hiPSC-CM phenotype and maturation. The first study showed that epicardial to epicardial-derived cells (EPC->DC)-containing EHTs exhibited increased force generation, enhanced electrophysiologic properties, and more mature CM phenotype, as seen by increased sarcomere length.[24] In another study, EPCs were co-cultured with cardiac progenitor cells with and without TGF-β inhibition (Floy et al., 2022). This study reported an increase in CM proliferation and an associated increase in sarcomere disarray expected from proliferating CM. However, the co-culture of EPCs with cardiac progenitors does not fully recapitulate embryonic development as the primary heart tube is already spontaneously contracting by E8.0 indicating they have the contractile machinery to be defined as CM (Tyser and Srinivas, 2020). Thus, a study determining the effect of epicardial and epicardial-derived cells on differentiated CM might more accurately recapitulate development. More recently, proepicardial cells have been combined with ventricular CM in aggregates, and these showed increased calcium handling and CM sarcomere length as well as IGF2-induced CM proliferation, indicating a role of EPCs in both CM maturation and proliferation (Tan et al., 2021). The tissues were cultured in a media formulated to reduce epicardial epithelial-to-mesenchymal transition (EMT) but there were still ∼8% uncharacterized cells in their culture indicating potential epicardial-EMT that TGF-β inhibitors might have ameliorated (Tan et al., 2021). The CM functional assessment in this study was limited to calcium and contractility and maturation markers were evaluated using bulk qPCR versus more powerful techniques such as single-cell RNA sequencing (scRNA-seq) necessary to determine cell-specific effects of the EPC on the ventricular CM (Tan et al., 2021).

The building literature and new technical advances provided a scientific premise to evaluate the hypothesis that embryonic-to-fetal multicellular interactions drive CM maturation in ways distinct from stimuli of the adult heart. Here we mimic fetal development by creating co-cultures of CM with fetal-like hiPSC-derived EPCs and find that they simultaneously enhance hiPSC-CM electrochemical function, proliferation, and fetal sarcomeric expression profiles. When fibroblasts, cells essential for post-natal heart maturation, are added to this multicellular experimental framework, proliferation is lost, electrochemical maturation is sustained, and sarcomere-mediated mechanical maturation is added. Thus, this work defines an intermediate condition on the road to CM maturation wherein electrochemical maturation, but not mechanical maturation can occur with CM proliferation.

RESULTS

Mouse embryonic CM maturation and proliferation at E10, E12, and E17

The degree of CM maturation that occurs during embryonic development is understudied. To explore this and the potential role of the epicardium in embryonic CM maturation, three embryonic time points were identified that correspond to well-known dynamics of the formation of the epicardium. E10 was chosen as it corresponds to a time point where the primary heart tube is spontaneously contracting and the proepicardial organ is fully formed but few EPCs have covered the heart tube (Rudat and Kispert, 2012; Vicente-Steijn et al., 2015). E12 corresponds to a time point where EPCs have fully encapsulated the heart to form the epicardium but substantial amounts of EMT have not occurred (Acharya et al., 2012; Vicente-Steijn et al., 2015). E17 corresponds to a

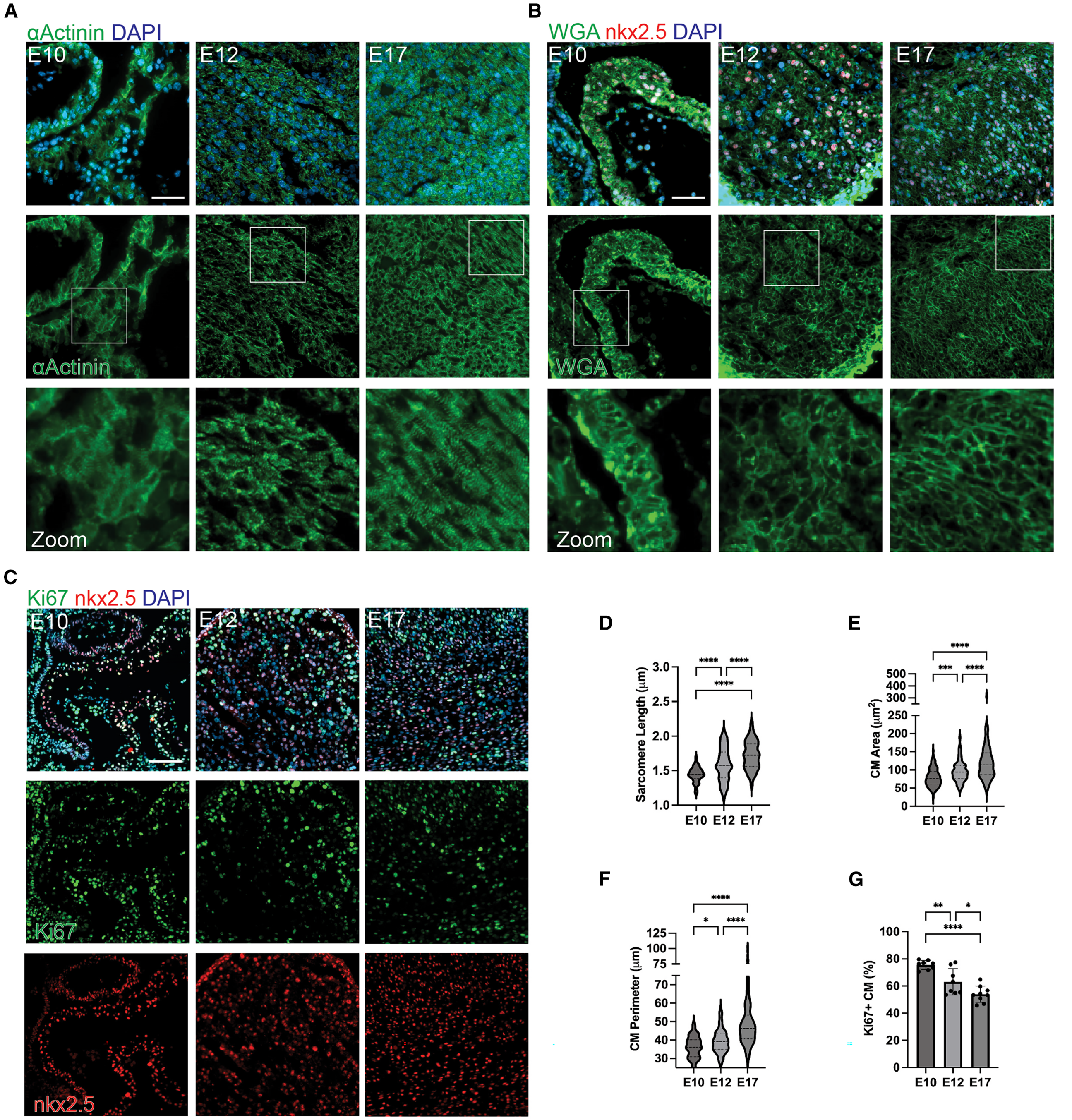

Figure 1. Mouse cardiomyocyte maturation during embryonic development is substantial

(A) Representative images from E10 (left), E12 (middle), and E17 (right) mouse hearts stained for α-actinin (green) for CM sarcomere visualization and DAPI (blue).

(B) Representative images of wheat germ glutenin (WGA; green) for cell membrane visualization, nkx2.5 (red) marking first heart field cardiomyocytes, and DAPI for nuclei visualization (blue) from E10 (left), E12 (middle), and E17 (right) mouse heart sections.

(C) Representative images of mouse heart sections stained for nkx2.5 (red) marking first heart field cardiomyocytes, Ki67 (green) marking proliferation, and DAPI (blue) marking the nucleus from E10 (left), E12 (middle), and E17 (right). The quantified average percent proliferating CM as determined by the co-localization for Ki67 with nkx2.5 and DAPI. Scale bar, 100 μm.

(D–G) (D) Quantified CM sarcomere length, (E) CM area, (F) CM perimeter, and (G) the percentage of proliferating CM at each developmental time point. For (D–F), the violin plot center dashed line represents the median and outer dashed lines represent upper and lower quartiles

(legend continued on next page)

time point where substantial amounts of EMT have occurred and stromal cells are present throughout the myocardium (Acharya et al., 2012).

During embryonic development, CM remains proliferative but almost entirely exits the cell cycle in the first week after birth (Bergmann et al., 2015). Thus, decreases in proliferation are a sign of advancing maturation in CM. CM proliferation was examined in parallel with common hiPSC-CM maturation parameters to see if the maturation indices (1) happened during embryonic development and (2) showed the same proliferation-maturation dichotomy as has been demonstrated in postnatal development (Singh et al., 2023). Sarcomere length was assessed using staining for α-actinin (Figure 1A). CM area and perimeter was assessed via wheat-germ agglutinin (WGA) for membrane visualization, nkx2.5 to mark CM, and DAPI staining (Figure 1B). Lastly, the percent of proliferating CM were visualized and quantified using the co-localization nkx2.5 with Ki67 and DAPI (Figure 1C). The sarcomere length increased substantially over these developmental time points from 1.44 ± 0.11 μm at E10 to 1.59 ± 0.23 μm at E12 and 1.73 ± 0.20 μm at E17 (Figure 1D). CM area and perimeter showed increases as well (Figures 1E and 1F). CM proliferation at E10 was the greatest (75% ± 3%) and decreased by ∼10% at E12 (63 ± 10%) and another 10% at E17 (54% ± 6%) (Figure 1G). These findings confirm that even at an early time point, when CM proliferation is high, CM proliferation is inversely related to maturation *in vivo*. They also confirm that substantial amounts of CM maturation occur during embryonic development; thus, hiPSC-CM can potentially be driven to mature by mimicking embryonic interactions with EPCs.

Epicardial co-culture composition in 2D

The effect of EPCs on hiPSC-CM maturation and function was first explored in 2D. EPCs were seeded on top of CM and the co-cultures were either maintained in TGF-β inhibitor (SB431542) to prevent EMT (CM + EPC[+SB]) or in basal epicardial media to allow spontaneous EMT into epicardial derived cells (CM + EPC->DC) to occur (Figure 2A). The CM + EPC->DC condition allowed us to study a situation in which the EPC population is gradually being diluted by the emergence of stromal cells, as in development. Extended exposure to ascorbic acid-containing media was used to drive a ventricular phenotype of the hiPSC-CM in all conditions to limit variation between other CM subtypes (Kim et al., 2023). Due to the expansion of the epicardial population in the presence of the TGF-β inhibitor, the CM + EPC(+SB) conditions contained 67% ± 14% cardiac troponin T (cTnT) positive CM and the CM + EPC->DC condition contained 78% ± 14% after 8 days of co-culture (Figure 2B). The CM population in the CM controls without (97% ± 2%) and with (96% ± 3%) TGF-β inhibition remained high (Figure 2B). Epicardial co-culture did not increase the percentage of ventricular CM in culture since all conditions contained ≥97% myosin light chain 2v positive (MLC2v) CM (Figure 2C).

Immunocytochemistry (ICC) for cTnT, Wilms tumor 1 (WT1), an epicardial marker, and DAPI were used to assess the percentage of EPCs remaining after 8 days of co-culture (Figure 2D). There was substantial migration of EPCs into the CM layer depicted by red nuclei interspersed within the cTnT positive area (Figure 2D). Additionally, large patches of highly confluent epithelial cells can be seen separate from the CM in bright field images (Figure S1A). Quantification of the WT1 cell population determined that the CM + EPC(+SB) condition contained 35% ± 11% WT1 positive cells while a significant portion of the EPCs in the EPC->DC conditions underwent EMT and only 10% ± 7% WT1 positive cells remained (Figure 2E). Three hiPSC-lines were used for these experiments and the maintenance of WT1 positive cells as well as the cTnT positive population is consistent between all three lines in each condition (Figures S1B–S1D). The $MLC2v^+$ CM population was consistent across the two lines tested (Figures S1E–S1G). These data serve to validate the composition of cells present in the 2D *in vitro* model.

Epicardial cells enhance CM proliferation in 2D

Since multiple animal studies show that EPCs are essential for CM proliferation during development, we aimed to explore this using hiPSC-derived cells. The co-cultures and controls were given ethynyl deoxy-uridine (EdU) for the last 24 h in culture and stained for both cTnT and EdU or Ki67 a nuclear transcription factor that marks cell proliferation (Figures S1H and S1I). The percent proliferation, as indicated by the $cTnT^+EdU^+$ population, increased from ∼20%, in both CM and CM(+SB) conditions, to 27% ± 2% in the EPC(+SB) conditions and 25% ± 4% in the EPC->DC condition (Figures 2F and 2G). This increase in proliferation was consistent with the expression of Ki67 (Figure 2H). Histograms of the CM population versus EdU or Ki67 were used to determine the percentage of proliferative CM (Figures 2I and 2J). This confirms that hiPSC-EPCs, which are maintained or are currently undergoing EMT,

of the distribution. For (G), the bar graph and error bars represent the mean ± SD and each dot represents the average percent Ki67 and nxk2.5 co-positive nuclei from multiple sections across E10 (*n* = 8), E12 (*n* = 8), and E17 (*n* = 9) embryonic mouse hearts. For (D), E10 (*n* = 87), E12 (*n* = 101), and E17 (*n* = 108) sarcomeres across E10 (*n* = 8), E12 (*n* = 8), and E17 (*n* = 9) embryonic mouse hearts were measured. For (E and F), E10 (*n* = 116), E12 (*n* = 122), and E17 (*n* = 128) were measured. *$p < 0.05$, **$p < 0.01$, ***$p < 0.001$, and ****$p < 0.0001$.

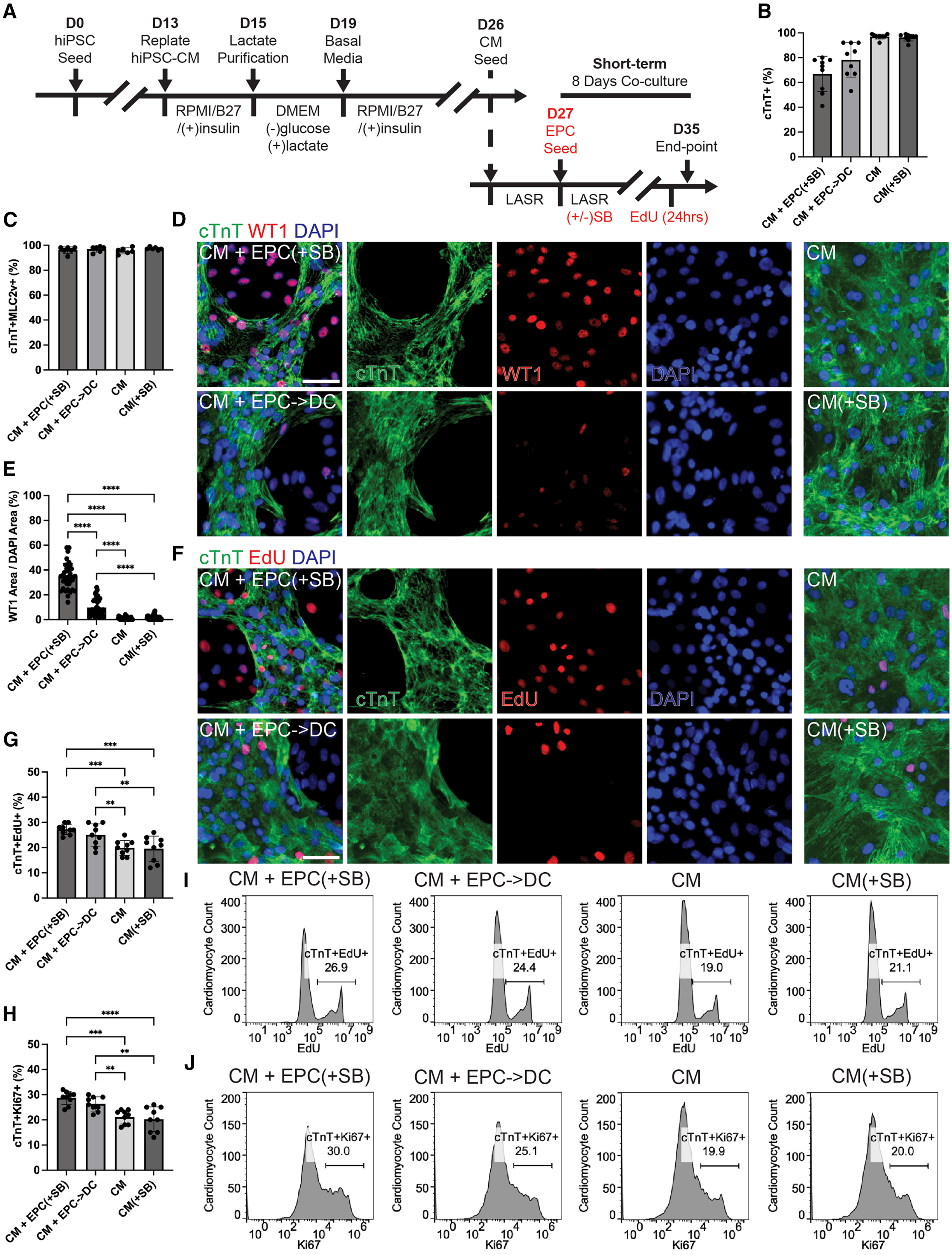

Figure 2. Epicardial cells increase CM proliferation in 2D co-cultures

(A) 2D co-culture experimental timeline where red text indicates experimental variables. Where either epicardial cells (EPCs) were seeded on day 27 or not, and each condition with or without EPCs was maintained in LASR media with or without TGF-β inhibitors (+/−SB). Cells fated for flow cytometry were incubated in EdU for 24 h before fixation while the others were assessed functionally.

(B) Flow cytometry for cardiac troponin T (cTnT) indicating the percentage of CM in each experimental condition.

(C) Flow cytometry for cTnT and myosin light chain 2 (MLC2v) indicating the percentage of ventricular CM in each condition.

(legend continued on next page)

both increase CM proliferation as in animal models during development. This finding was consistent for the CM + EPC (+SB) condition in all three hiPSC-lines (Figures S1J and S1K). It was also consistent for the CM + EPC->DC condition for the M4 and F7 lines but not the CCND2-hiPSC line. We noted that the co-cultures from the CCND2 line exhibited a lower percentage of EPCs in the CM + EPC->DC condition (Figure S1B). This could reflect a more robust EMT of the CCND2-EPCs (Figure S1C). In summary, EPCs spur proliferation, but a large enough dose may be required to do so, and that dose might be dependent on the characteristics of the stem cell source line.

Epicardial cells enhance select indices of CM functional maturation in 2D

To determine whether and to what extent functional maturation of CM in the epicardial and epicardial-derived cell co-cultures occurred, calcium transient (CaT) assessment was conducted at the experimental endpoint. From embryonic to post-natal and adult development, there are stark increases in the amount and rate of release and sequestration of calcium from the sarcoplasmic reticulum as calcium is the key mediator of excitation-contraction coupling (Liu et al., 2002). The CM co-cultures calcium transients were assessed using calcium sensitive dye under paced conditions (Figure 3A). Most notably, the presence of EPCs resulted in a stark decrease in CaT time to peak (Figure 3B). The robust decrease in time to peak for the epicardial co-culture conditions was consistent across all three hiPSC-lines (Figure S3). Overall, the CM + EPC(+SB) condition showed the most mature calcium handling dynamics followed by the CM + EPC->DC condition. Both conditions showed increased maximum amplitude and CaT upstroke velocity (Figures 3C and 3D). This increase was seen across all three hiPSC-lines in the CM + EPC(+SB) condition but was lacking in the CCND2 CM + EPC->DC condition most likely due to the lowest remaining amount of EPCs in this line (Figure S3). There was also a significant increase in downstroke velocity for the CM + EPC(+SB) condition on average (Figures S2A–S2C).

To further assess the electrophysiologic properties of CM in the CM + EPC(+SB) condition and its control, CM(+SB), the cells were replated, and patch clamp for CM action potential (AP) was performed (Figure 3E). The APs of ventricular hiPSC-CM have slower upstroke velocities during the rapid depolarization phase, lack of a "notch," and shorter plateau phases due to immature ion channel compositions in comparison to adult CM (Goversen et al., 2018). The CM from the epicardial co-culture had a significant decrease in beat rate frequency (f) (Figure 3F). Both CM(+SB) and CM + EPC(+SB) have a long AP duration at 90% repolarization (APD90) of ~800 ms, which is characteristic of ventricular CM (Figure 3G). Consistent with the CaT data, the maximum upstroke velocity was almost doubled in the CM + EPC(+SB) (Figure 3H). Additionally, a decrease in maximum diastolic potential (MDP) from -65 ± 6 to -69 ± 3 mV was seen in the epicardial co-cultures suggesting a trend toward the resting potential of mature adult ventricular CM of -85 mV (Guo and Pu, 2020). In summary, patch clamp data further support increased electrochemical maturation of CM in epicardial co-cultures.

Another functional attribute of maturation is increased force generation at the tissue and cellular levels (Wheelwright et al., 2018). To explore the cellular level mechanical function of the hiPSC-CM, the co-cultures were singularized and traction force microscopy (TFM) was performed (Figures 3J and 3K). There were no significant differences between the CM-only conditions and the EPC co-culture conditions. There was, however, a significant decrease in force in the CM(+SB) condition compared to the CM condition (Figure 3L). The negative effect of the TGF-β inhibitor were ameliorated by the positive effect of the EPCs (Figure 3L). However, when normalized to the CM area, there are no significant differences in CM stress generation (Figure 3M). The force generation data are broken down by line in the supplement (Figures S2D–S2F). These data indicate that the effect of EPCs on CM maturation is more substantial with regard to electrochemical parameters and does not appear to include mechanical maturation at a cellular level.

Epicardial cells enhance CM morphologic maturation in 2D

We next evaluated the CM of the co-cultures for morphologic features of maturation. The sarcomeres were visualized via staining for α-actinin and the ventricular

(D) Representative IHC images from M4-hiPSC 2D co-cultures at day 35 stained for cTnT (green), WT1 (red—epicardial cells), and the nucleus (DAPI—blue).
(E) Image quantification of WT1 area/DAPI area to determine the percentage of epicardial cells in all four conditions.
(F) Representative images of cTnT (green) and proliferating cells after 24-h EdU incubation (red).
(G) Flow cytometry quantification of $cTnT^{+}EdU^{+}$ and (H) $cTnT^{+}Ki67^{+}$ percentage for all experimental replicates.
(I and J) Representative M4 flow cytometry histograms of co-cultures with visualization of the cTnT+ population (CM count) with (I) EdU or (J) Ki67 on the x axis. Scale bars, 50 μm. Bar graphs represent the mean ± SD.
For (B, E, G, and H), $n = 3$ independent experiments for each hiPSC-line (M4, F7, and CCND2) and (C) $n = 3$ independent experiments for two of the hiPSC-lines (M4 and F7). $^{*}p < 0.05$, $^{**}p < 0.01$, $^{***}p < 0.001$, and $^{****}p < 0.0001$ for (C, E, G, and H). See also Figure S1.

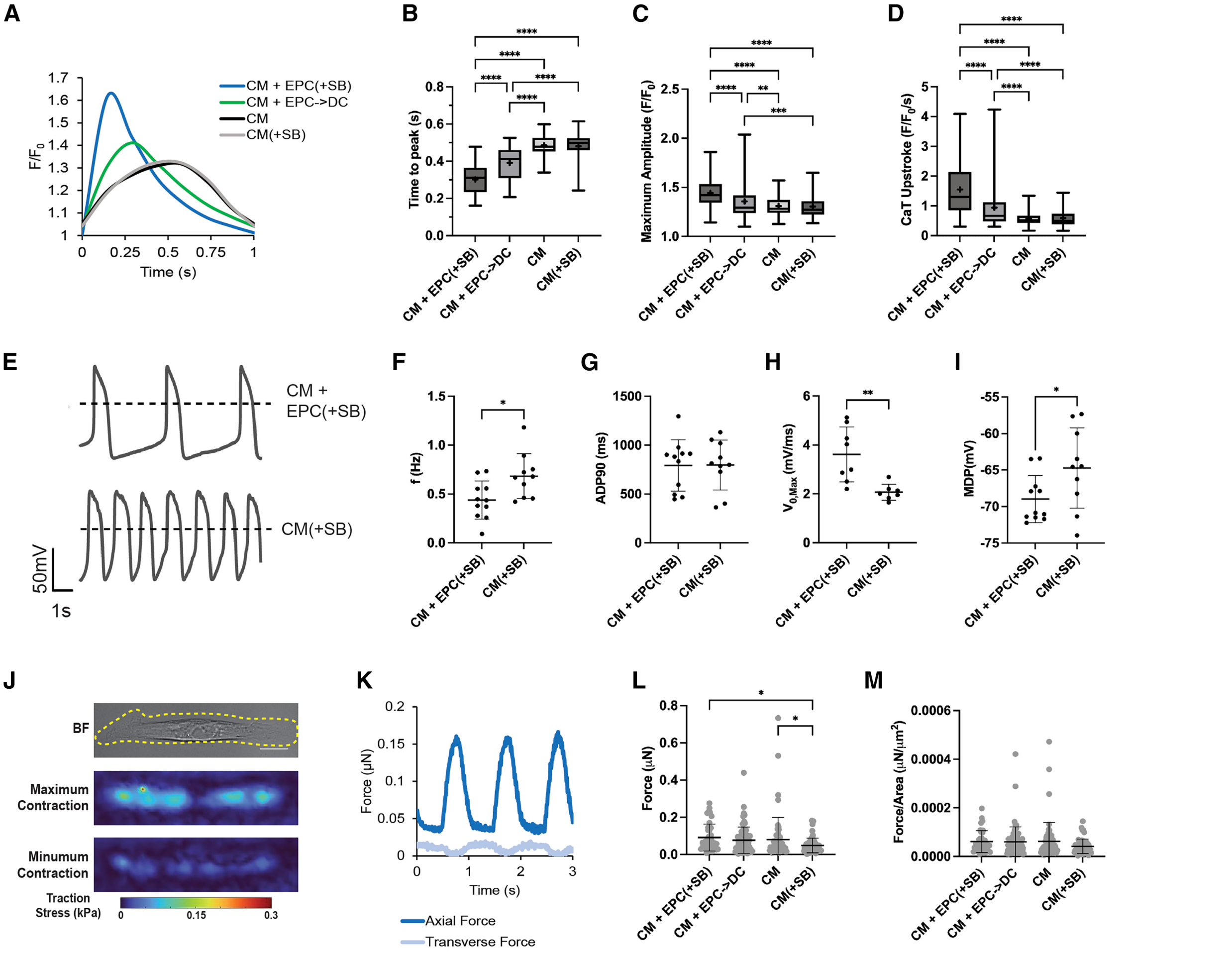

Figure 3. Epicardial cells enhance CM function in 2D co-cultures

(A) Representative direct co-culture calcium transient (CaT) traces of CM from the M4-line for each condition.

(B–D) Quantification of CaT parameters (B) time to peak, (C) maximum amplitude, and (D) CaT upstroke velocity.

(E) Representative patch clamp trace for the CM + EPC(+SB) and CM(+SB) conditions.

(F–I) Quantification of patch clamp parameters (F) beating frequency (f), (G) the AP duration at 90% repolarization (APD90), (H) the AP maximum upstroke velocity ($V_{0,max}$), and (I) the maximum diastolic potential (MDP).

(J) The maximum and minimum traction stress maps for a representative CM determined using traction force microscopy (TFM).

(K) Axial and transverse dorce versus time plot for three contractions of a representative cell. Scale bars, 20 μm.

(L and M) (L) CM contractile force determined by TFM and (M) CM contractile force normalized to ell area.

(B–D) Box and whisker plots with + marking the mean. (B–D) Data compiled from $n = 3$ independent experiments from each hiPSC-lines (M4, F7, and CCND2). For (F–I) and (L and M), center lines and error bars represent the mean ± SD. For (F, G, and I), CM + EPC(+SB) ($n = 11$) and CM(+SB) ($n = 10$); for (H), CM + EPC(+SB) ($n = 8$) and CM(+SB) ($n = 7$) cells across $n = 3$ independent experiments from the M4-line; and for (L and M), CM + EPC(+SB) ($n = 41$), CM + EPC->DC ($n = 74$), CM ($n = 60$), and CM(+SB) ($n = 54$) across $n = 3$ independent experiments from each hiPSC-line. *$p < 0.05$, **$p < 0.01$, ***$p < 0.001$, and ****$p < 0.0001$. See also Figure S2.

phenotype was validated with MLC2v staining (Figure 4A). The adult myocardium has an average sarcomere length of 2.25 μm (Sonnenblick et al., 1967). The longest sarcomere length was detected in the CM + EPC(+SB) co-cultures at 1.94 ± 0.17 μm followed by 1.87 ± 0.16 μm in the CM + EPC->DC condition, while the CM condition had an average length of 1.85 ± 0.17 μm (Figure 4B). Without the aid of EPCs, the TGF-β inhibition resulted in a significant decrease in CM(+SB) sarcomere length (1.81 ± 0.18 μm). The increase in CM sarcomere length with EPC co-culture was consistent across all three hiPSC-lines whereas TGF-β inhibition only negatively affected sarcomere length in the M4-hiPSC line (Figures S2G–S2I).

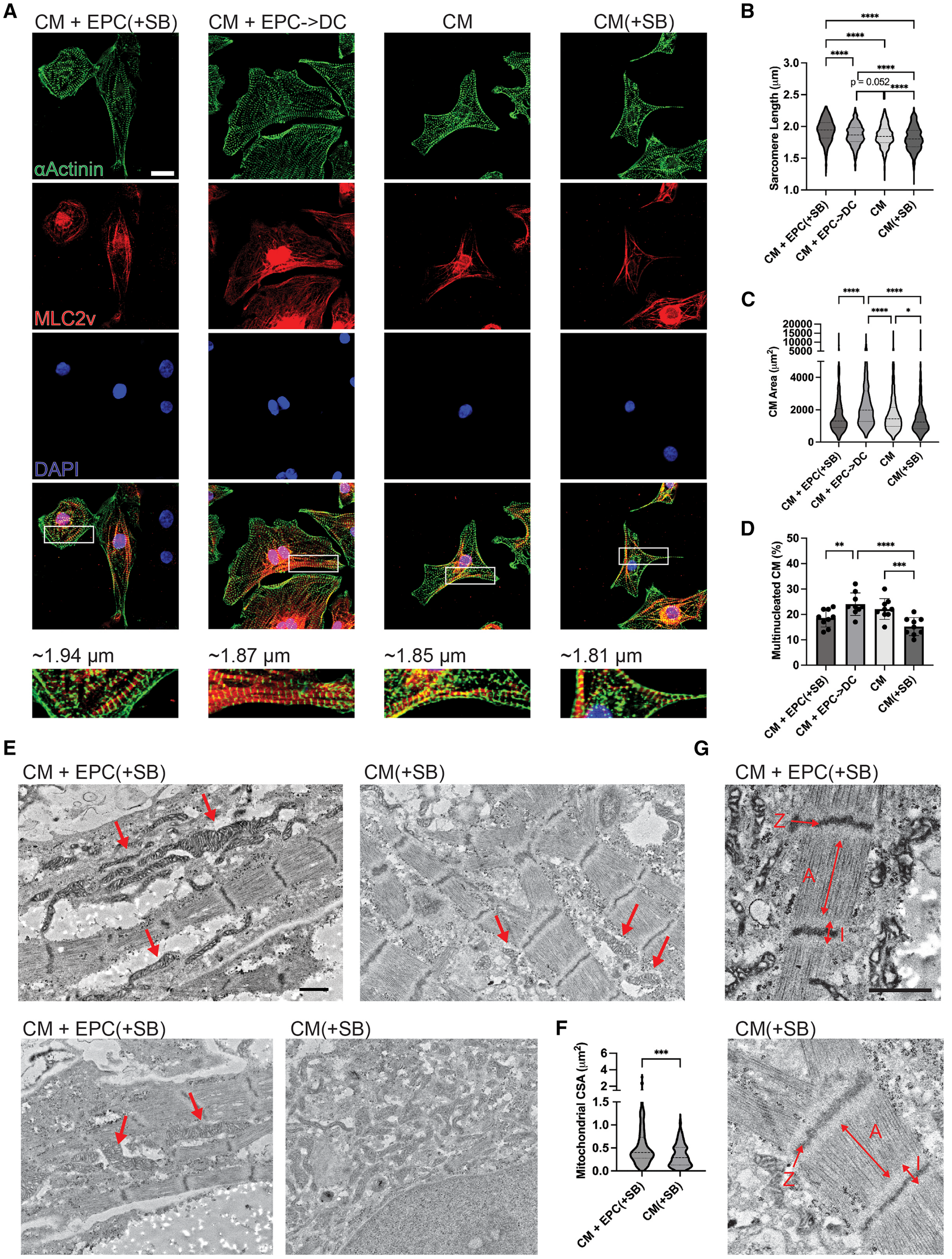

Figure 4. Epicardial cells modestly increase CM morphologic maturation

(A) Representative IHC images of 2D co-cultures replated onto glass slides and stained with α-actinin (green) for sarcomere visualization, MLC2v for ventricular phenotype validation (red), and DAPI (blue) for nuclear visualization. Scale bars, 20 μm.

(B–D) Violin plots showing (B) quantified sarcomere length (C) CM area, and (D) the percent multinucleated CM.

(legend continued on next page)

Additionally, the epicardial-derived co-culture resulted in CM hypertrophy, an indicator of maturation, as seen by a significant increase in CM area (Figure 4C). This could indicate a specific role of epicardial-derived cells on CM hypertrophy not afforded by the EPCs. The increased CM area in the CM + EPC->DC condition is consistent across all three hiPSC-lines (Figure S5). Another morphologic indicator of maturation is the percent of multinucleation in hiPSC-CM as the adult human heart contains ~26% multinucleated CM (Bergmann et al., 2015). No significant increases in multinucleation were seen in the epicardial co-cultures (Figure 4D). Though TGF-β inhibition caused a significant reduction in the percentage of multinucleated CM in the CM(+SB) condition, the presence of EPCs ameliorated this negative effect. The results were similar in all three hiPSC-lines (Figures S2G–S2I). In summary, in the CM + EPC(+SB) condition, CM showed increases in sarcomere length, while in the CM + EPC->DC condition, the CM showed increased hypertrophy; this outcome indicates differential effects of EPCs and EPDCs on hiPSC-CM phenotype.

Since the most significant maturation effects were seen in the CM + EPC(+SB) condition, transmission electron microscopy (TEM) was conducted on the CM + EPC(+SB) conditions and its control, CM(+SB), to better visualize CM ultrastructure as well as to quantify the size of mitochondria present. The mitochondria of CM + EPC(+SB) tended to be in direct apposition to sarcomeres, a characteristic of maturing CM, whereas the mitochondria of CM(+SB) were present most frequently in separate clusters (Dorn et al., 2015) (Figure 4E). The CM + EPC(+SB) condition had a significant increase in mitochondrial cross-sectional area in comparison to the CM(+SB) control (Figure 4F). Mature adult-CM sarcomere TEM images contain the distinct presence of Z-lines, I-bands, A-bands, and M-lines (Ronaldson-Bouchard et al., 2018). Both conditions have the distinct presence of Z-lines, I-bands, and A-bands but lack the presence of M-lines in CM (Figure 4G). The M-line typically appears last with long-term culture, 3D cultures, and extended exposure to electrical stimulation and so was not expected here (Ronaldson-Bouchard et al., 2018).

scRNA-seq reveals a unique population of vCM in both epicardial co-cultures

To further understand the genetic landscape of the CM in the co-cultures scRNA-seq was conducted on all 4 conditions in duplicate. The cells from all the conditions were merged and 14 clusters were identified (Figure 5A). The proportions of cells in each cluster by conditions can be visualized in the supplemental information (Figure S3A). These were identified as seven cell type categories: the ventricular CM (vCM; clusters 0, 3, 5, 9, and 10), proliferative vCM (clusters 2, 6, and 7), EPC (cluster 1), proliferative EPC (cluster 8), EPC->DC (clusters 4 and 11), atrial CM (aCM; cluster 12), and non-differentiated cells (cluster 13) that represent the 1%–4% cTnT-negative population from the initial hiPSC-differentiation (Figure 5B). The identity of each cluster was determined by looking at the differentially expressed genes (DEGs) between each cluster (Figure S3B). In particular, the vCM expressed cardiac markers as well as the ventricular-specific myofilament isoform *MYL2*. The proliferative vCM had similar gene expression as the vCM but also expressed markers related to mitotic cell division, chromosome segregation, and/or cytokinesis. The aCM expressed general cardiac markers, *TNNT2*, and the atrial marker natriuretic peptide A (*NPPA*) but lacked expression of *MYL2*. The proportion of cell types across conditions shows a large population of EPC in the CM + EPC (+SB) and a genetically distinct population of cells emerging in the CM + EPC->DC conditions labeled EPC->DC (Figure S3C). To guide analysis, the 9 clusters expressing CM marker genes were grouped into 6 categories: (1) $vCM_{CM\text{-}only\_bulk}$ (cluster 0); (2) $vCM_{EPC(+SB)\_bulk}$ (cluster 3); (3) $vCM_{EPC\text{->}DC\_bulk}$ (cluster 5); (4) proliferative vCM (clusters 2, 6, and 7); (5) vCM (clusters 9 and 10), and (6) aCM (cluster 12) (Figure 5C). The majority of CM from the CM-only conditions fall into the $vCM_{CM\text{-}only\_bulk}$ category while 69% and 17% of the CM in the CM + EPC(+SB) and CM + EPC->DC conditions respectively fall into the $vCM_{EPC(+SB)\_bulk}$ category (cluster 3). The majority of the CM from the CM + EPC->DC condition falls into the $vCM_{EPC\text{->}DC\_bulk}$ category (Figure 5C). Separate clustering of vCM from the

(E and F) (E) Representative TEM images with red arrows indicating mitochondria in close apposition to sarcomeres and (F) the quantified mitochondrial cross-sectional area (CSA).

(G) TEM image of sarcomeres showing the Z-line (Z), I-band (I), and A-band (A) for each condition. Scale bars, 1 μm.

In (B, C, and F), violin plots center dashed line represents the median, and outer dashed lines represent the upper and lower quartiles. In (B and C), the data represent CM across $n = 3$ independent experiments for all three hiPSC-lines (M4, F7, and CCND2); for (B), CM + EPC(+SB) ($n = 784$), CM + EPC->DC ($n = 839$), CM ($n = 799$), and CM(+SB) ($n = 713$); and for (C), CM + EPC(+SB) ($n = 878$), CM + EPC->DC ($n = 878$), CM ($n = 873$), and CM(+SB) ($n = 817$). For (D), the bar represents the mean ± SD for $n = 3$ independent experiments for all three hiPSC-lines (M4, F7, and CCND2) and each dot represents the averages of 5 fields of view across three wells for each independent experimental replicate. In (F), the CSA of CM + EPC(+SB) ($n = 79$) and CM(+SB) ($n = 81$) mitochondria across 10 cells per condition for $n = 1$ independent experiment from the M4-hiPSC line was measured. *$p < 0.05$, **$p < 0.01$, ***$p < 0.001$, and ****$p < 0.0001$. See also Figure S2.

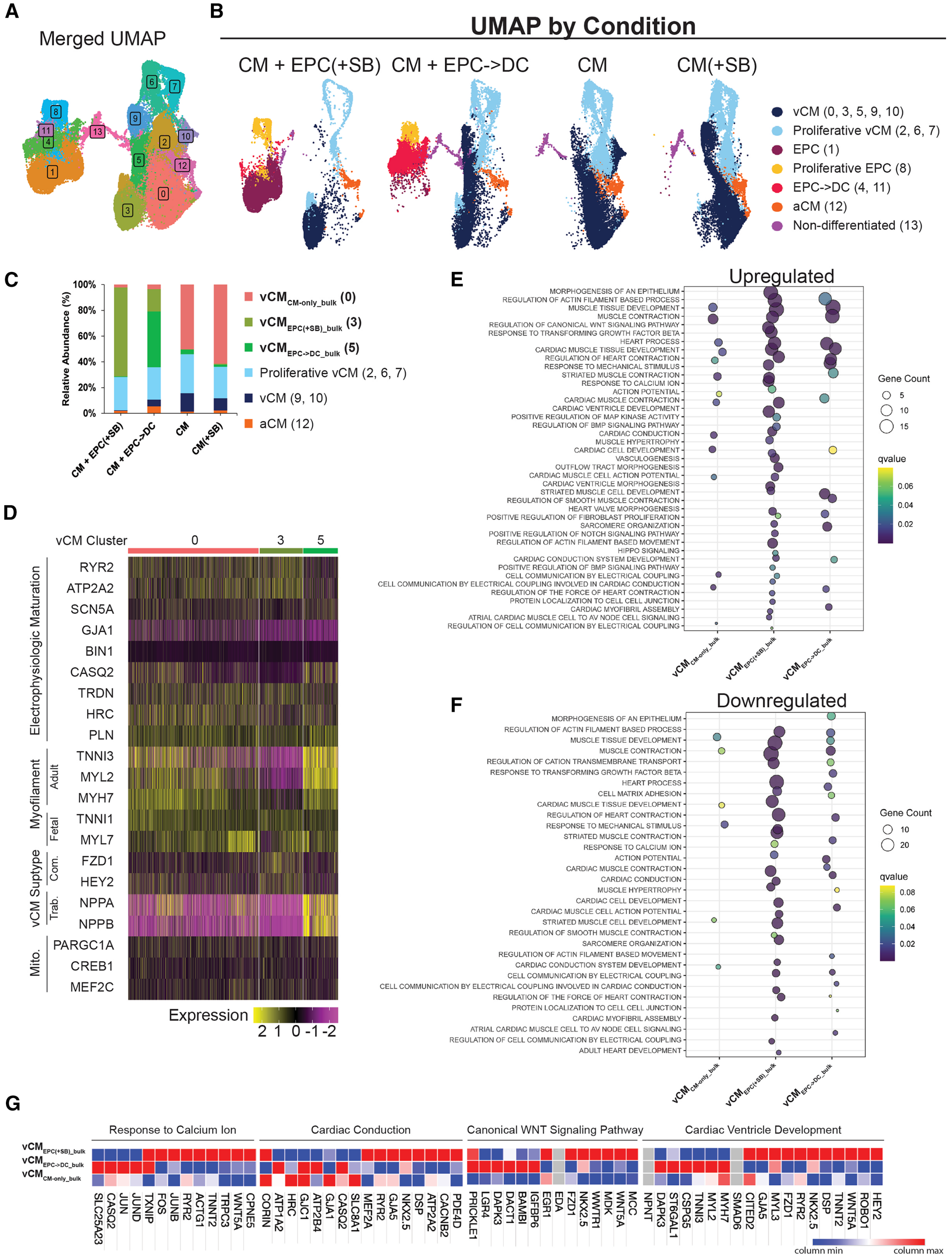

Figure 5. Single-cell RNA-seq reveals unique vCM phenotype in epicardial and epicardial derived co-cultures

(A) Single-cell RNA-seq UMAP of clustering where 14 different clusters (0–13) were identified.

(B) UMAP of each condition where the clusters were labeled into 7 major cell identities: ventricular CM (vCM; clusters 0, 3, 5, 9, and 10), proliferative vCM (clusters 2, 6 and 7), epicardial cells (EPCs; cluster 1), proliferative EPCs (cluster 8), epicardial to epicardial-derived cells (EPC->DC; clusters 4 and 11), atrial CM (aCM; cluster 12), and non-differentiated cells (cluster 13).

(legend continued on next page)

co-cultures indicated significant differences in gene expression stimulated by EPC and EPC->DC in the co-cultures.

To further understand these genetic shifts, genes related to CM maturation were examined, between the $vCM_{CM\text{-}only\_bulk}$, $vCM_{EPC(+SB)\_bulk}$, and $vCM_{EPC\text{->}DC\_bulk}$ (clusters 0, 3, and 5). Largely consistent with functional characterization, the $vCM_{EPC(+SB)\_bulk}$ (cluster 3) showed an increase in the key electrochemical maturation genes ryanodine receptor 2 (*RYR2*), ATPase sarcoplasmic reticulum calcium transporter 2 (*ATP2A2*) and triadin (Figure 5D) (Clemens et al., 2023; Guo et al., 2023). Surprisingly, they also showed a marked decrease in other key electrochemical maturation genes, such as calsequestrin 2 (*CASQ2*), histidine-rich calcium-binding protein (*HRC*), and phospholamban (*PLN*) (Arvanitis et al., 2011; G. Chen et al., 2015; J. Liu et al., 2009). Additionally, though sarcomere length was increased in the CM + EPC(+SB) co-culture, there was a decrease in the adult ventricular myofilament isoforms cardiac muscle troponin I (*TNN13*), myosin light chain 2 ventricular form (*MYL2*), and myosin heavy chain 7 (*MYH7*). Nonetheless, the vCM were more specified showing increases in the compact ventricular markers frizzled 1 (*FZD1*) and the related family bHLH transcription factor with YRPW motif 2 (*HEY2*) along with decreases in trabecular ventricular myocardium markers NPPA and natriuretic peptide B (*NPPB*) (Funakoshi et al., 2021; Li et al., 2016). Conversely, the $vCM_{EPC\text{->}DC\_bulk}$ (cluster 5) showed a distinct increase in the adult myofilament isoforms *TNN13* and *MYL2* with an accompanying decrease in the fetal isoforms skeletal muscle troponin I (*TNNI1*) lacking in the $vCM_{CM\text{-}only\_bulk}$; this fits with the CM hypertrophy seen in the morphological assessment. Interestingly, the $vCM_{EPC\text{->}DC\_bulk}$ (cluster 5) also showed high expression of the trabecular myocardial markers *NPPA* and *NPPB*. These shifts corresponded to an increase in the electrophysiologic maturation marker *CASQ2* and *PLN* but decreases in *RYR2* and *ATP2A2*. These results indicate differential enhancement of electrophysiologic and myofilaments maturation in vCM spurred by either EPCs or EPDCs.

The $vCM_{CM\text{-}only\_bulk}$ did not have a defined up- or downregulation of compact or trabecular markers consistent with early fetal development before ventricular patterning is spurred by the endocardium and epicardium (Tian et al., 2017). The same heatmap was made for all the vCM pooled by condition (Figure S4A). In this iteration, the trends are the same as described previously, but the CM + EPC->DC condition has a portion of cells resembling the $vCM_{EPC(+SB)\_bulk}$ in addition to the $vCM_{EPC\text{->}DC\_bulk}$. This outcome is consistent with the composition of the CM + EPC->DC condition wherein most, but not all, EPCs undergo EMT. The proliferative vCM show a similar heatmap but with muted expression patterns due to the downregulation of cardiac muscle genes during the proliferation process (Figure S5A).

Differentially expressed genes and pathways analysis of vCM in epicardial co-cultures

DEGs were determined between each vCM cluster (clusters 0, 3, 5, 9, and 10) (Figure S3D). The largest number of DEGs was present in the $vCM_{EPC(+SB)\_bulk}$ (cluster 3), followed by the $vCM_{EPC\text{->}DC\_bulk}$ (cluster 5, Table S1). Over-representation analysis (ORA) of gene ontology terms related to biological processes was performed, resulting in many pathways being up- and downregulated between the different vCM clusters (Table S1). For the $vCM_{EPC(+SB)\_bulk}$ multiple pathways related to calcium ion handling, cardiac conduction, and ventricular development were upregulated (Figure 5E). Conversely, some of these pathways were also downregulated, particularly those related to heart contraction due to decreases in myofilament genes MYL2 and TNNI3 (Figure 5F). Additionally, pathways related to key signaling cascades that occur during heart embryogenesis were upregulated in the $vCM_{EPC(+SB)\_bulk}$ such as WNT, bone morphogenic protein, Notch, and MAPK and Hippo signaling pathways. These signaling pathways are all highly interconnected and multiple of the same DEGs occur in each pathway. Of particular interest are the WNT signaling pathways because of the role they play in CM proliferation and specification of the compact myocardium *in vivo* and *in vitro* (Buikema et al., 2020; Fan et al., 2018; Funakoshi et al., 2021). The $vCM_{EPC\text{->}DC\_bulk}$ showed significant increases in pathways related to myofibrils and muscle contraction but downregulation in some conduction-related pathways (Figures 5E and 5F). The $vCM_{CM\text{-}only\_bulk}$ had the fewest upregulated pathways and among them were a few pathways related to conduction due to increased expression of *GJA1*, *SLC8A1*, and *HRC*

(C) Graph of the percentage of CM that fall into each of the 6 CM categories listed.

(D) Heatmap of cardiac genes of interest for the bulk vCM clusters present in the CM-only controls (cluster 0), CM + EPC(+SB) (cluster 3), and CM + EPC->DC (cluster 5).

(E and F) ORA for biological processes (BP) showing a dot plot for the (E) upregulated and (F) downregulated pathways for the three bulk vCM clusters as compared to all other vCM clusters.

(G) Heatmap of some of the differentially expressed genes contributing to enrichment in four BP pathways: (1) cardiac ventricle development; (2) regulation of canonical WNT signaling pathway; (3) cardiac conduction; and (4) response to calcium ions. See also Figures S3–S5.

(Figures 5E and 5F). A heatmap showing specific genes that were up- and downregulated in four of the enriched pathways shows some of the key DEGs that contributed to the enrichment (Figure 5G). DEGs and pathways enrichment was also done for the vCM by condition (Figures S4B–S4E; Table S1). The same pathways of interest were identified in this analysis. When the proliferative vCM were analyzed by condition, there were fewer DEGs and BP pathways that were enriched (Figures S5B–S5E; Table S1). However, the ones that were statistically significant confirm results seen when the vCM are analyzed by cluster and by condition.

Overall, these results indicate a significant genetic shift in vCM occurs when they are co-cultured with EPC or EPC->DCs. EPCs promote a more fetal-like but electrically conductive vCM population, which could be driven by the upregulation of the WNT signaling pathway. The EPC->DC co-cultures bring a population of vCM further along the developmental progression where they are electrically conductive (though with gene expression distinct from EPC co-cultures), beginning to express adult myofilament isoforms, and with a population of both trabecular and compact myocardium-like vCM. Next, we moved onto a 3D EHT model to study the effects of EPCs in a more complex system.

CM proliferation and function in EHTs generated with epicardial and epicardial derived cells

To determine whether the impact of EPCs on CM proliferation and maturation could persist in the context of the more intricate interplay of cell and matrix in a 3D tissue, EHTs were generated with the same four conditions used in 2D and assessed for proliferation and function after 29–30 days of culture (Figures 6A and 6B). In addition, a CM Tri-culture condition was added that included CM, EPCs, and fully differentiated epicardial-derived fibroblasts (EPD-FBs) that predominate in the postnatal myocardium. The results for this condition are described in detail later. After 29–30 days of co-culture and similar to 2D, EPCs were still present in the CM + EPC(+SB) condition and minimally present in all other conditions (Figure 6C). EHT proliferation was assessed with cryosections that were co-stained for cTnT and EdU (Figure 6D). After 30 days of culture, there were no differences in proliferation between any of the conditions (Figure 6E). The proliferation of all conditions was substantially decreased from the 2D short-term culture to an average of 5%–7% indicative of more advanced maturation of CM in 3D and a transient effect of EPCs on CM proliferation that eventually subsides in the context of 3D tissue development.

As in 2D, CaT measurements were taken from all five conditions (Figure 7A). Like 2D, all the co-culture conditions resulted in a robust decrease in time to peak indicating more mature calcium handling (Figure 7B). In 3D, the CM(+SB) condition also resulted in a slight decrease in time to peak but a marked decrease in all other parameters such as maximum amplitude and CaT upstroke velocity (Figures 7C and 7D). Differing from the 2D results, the long-term 3D co-cultures required epicardial-derived cells (CM + EPC->DC) to attain a significant increase in maximum amplitude and CaT upstroke velocity (Figures 7C and 7D). This effect may be driven by a combination of epicardial-derived signaling promoting maturation (Bargehr et al., 2019) and the absence of long-term TGF-β inhibition (Umbarkar et al., 2019), which was used in the CM + EPC (+SB) condition and may have impaired long-term function. To further explore the tissue level electrophysiologic maturation, optical mapping was conducted for the CM + EPC->DC condition in comparison to a CM-only control. Maps of APD80 showed extended APD80 in the ventricular CM range for all conditions (Figure 7E). Upon quantification, the longest APD80 was present in the CM-only control (Figure 7F). The maximum upstroke velocity (dV/dt Max) was mapped and quantified, showing a strong but not significant trend upwards in the CM + EPC->DC condition ($p = 0.055$) (Figures 7G and 7H). Lastly, activation time maps were plotted to determine if there was signal propagation from one end of the EHT to another with point electrical stimulation (Figure 7I). If propagation was present, the conduction velocity across the EHT could be calculated. The conduction velocity showed a significant increase in the CM + EPC->DC condition (Figure 7J). Representative AP traces show a more regular ventricular AP shape of the co-culture AP and the reduced upstroke present in some of the CM-only APs (Figures 7K–7M).

Tissue level mechanical function was determined by assessing the twitch force of the EHTs. The long-term incubation with TGF-β inhibitors was detrimental to force generation and both conditions maintained in TGF-β inhibitors did not have a detectable force (Figure 7N). Both CM and CM + EPC->DC either had no detectable force or produced small forces in the 0.01–0.02 mN range (Figure 7N). A similar trend was seen in twitch stress generation (Figure 7O). Taken together, these data largely mirror those of 2D. While EPCs play a role in electrophysiologic maturation in 3D, they do not support robust tissue-level force generation.

Addition of an epicardial-derived fibroblast population in combination with EPCs results in improved force and electrophysiologic function

To determine the impact of a more advanced embryonic-to-fetal developmental stage, EPD-FBs were added to EHTs containing CM and EPCs (CM Tri-culture). In particular, EPCs were maintained in TGF-β inhibitors for 9 days, mimicking

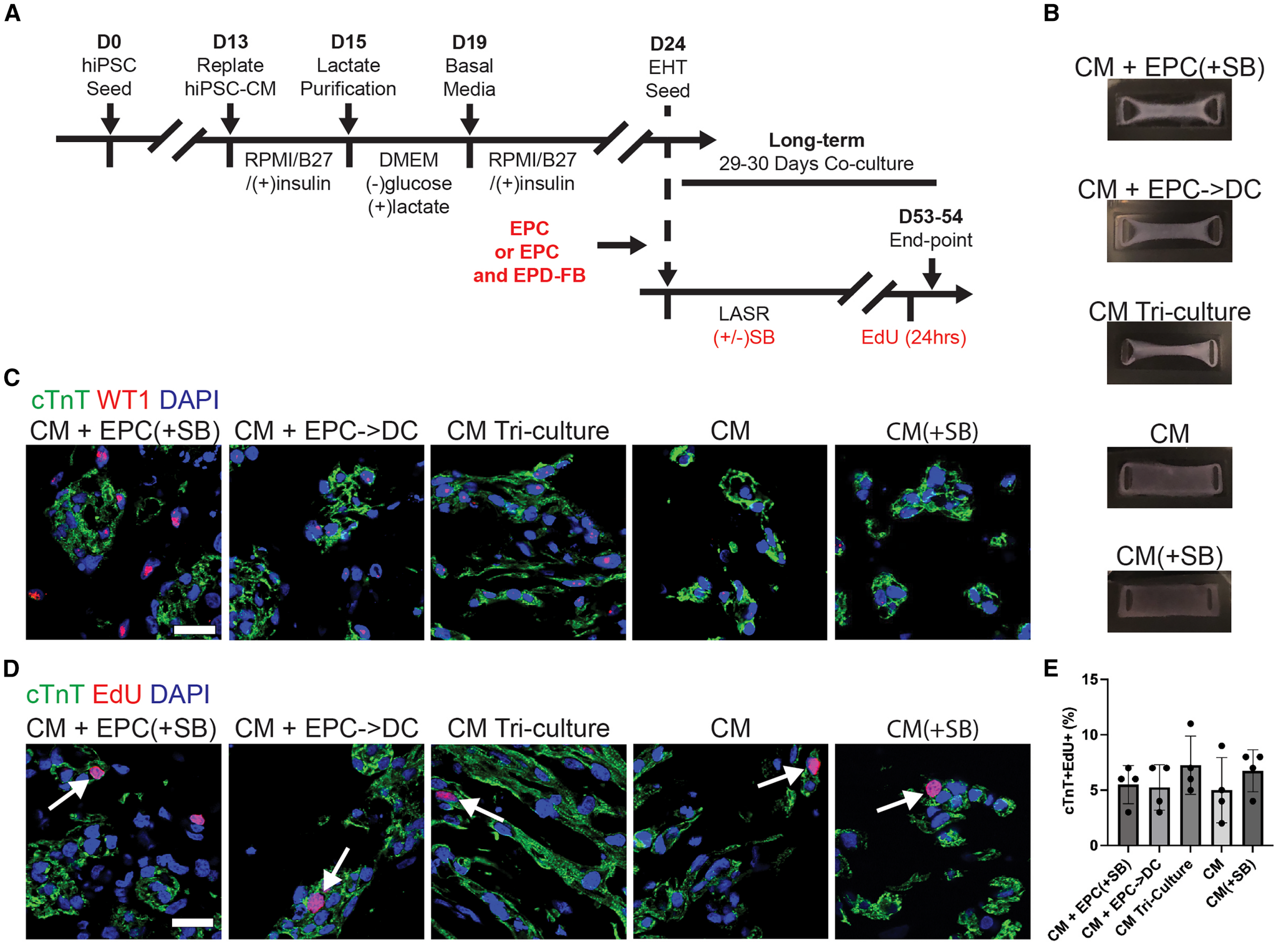

Figure 6. Epicardial engineered heart tissue proliferation

(A) Experimental timeline for engineered heart tissue seeding where the red text represents experimental variables.

(B) Images of engineered heart tissues at day 29 or 30 in PDMS molds.

(C) Representative EHT images of WT1 (red), cTnT (green), and DAPI (blue).

(D) Representative images of cTnT (green), EdU (red), and DAPI (blue) where the white arrows point to proliferating CM.

(E) The quantified EHT CM proliferation via the percentage of $cTnT^{+}EdU^{+}$ cells. Scale bars, 20 μm. In (E), the bar and error bars represent the mean ± SD where each dot represents the average across three sections of one EHT and $n = 4$ EHTs across two independent experiments for each condition was assessed. $^{*}p < 0.05$, $^{**}p < 0.01$, $^{***}p < 0.001$, and $^{****}p < 0.0001$.

the 2D condition, and then the TGF-β inhibitors were removed allowing epicardial EMT. It was necessary to differentiate the EPD-FB separately as the EPCs used in this study lacked *TCF21* and did not spontaneously undergo EMT into fibroblasts on their own. The lack of FB in the EPC->DC conditions was validated by staining for TE7, a fibroblast marker, in the 2D and 3D co-cultures (Figures S6A–S6C). The addition of fibroblasts did not change CM proliferation in the EHTs (Figure 6E). When CaT were assessed for the CM Tri-culture, similar improvements were present as in the CM + EPC->DC conditions (Figures 7A–7D). Videos of the calcium handling for all five conditions can viewed in Videos S1–S5. Optical mapping of voltage determined a significant decrease in the average EHT APD80 as well as a significant increase in the AP dV/dt Max (Figures 7E–7H). All CM Tri-culture EHTs measured did have a measurable conduction velocity (Figures 7I and 7J). A representative AP trace of the CM Tri-Culture EHTs shows a similar shape to the CM + EPC->DC condition (Figure 7L). Most significantly, the addition of the fibroblasts to the EHTs resulted in an order-of-magnitude increase in twitch force and stress generation (Figures 7N and 7O). Taken together, when the last major cellular component of myocardial development is added to engineered cardiac mimics containing EPCs and EPC->DCs, proliferation is lost, electrophysiology is maintained, and force generation is significantly enhanced.

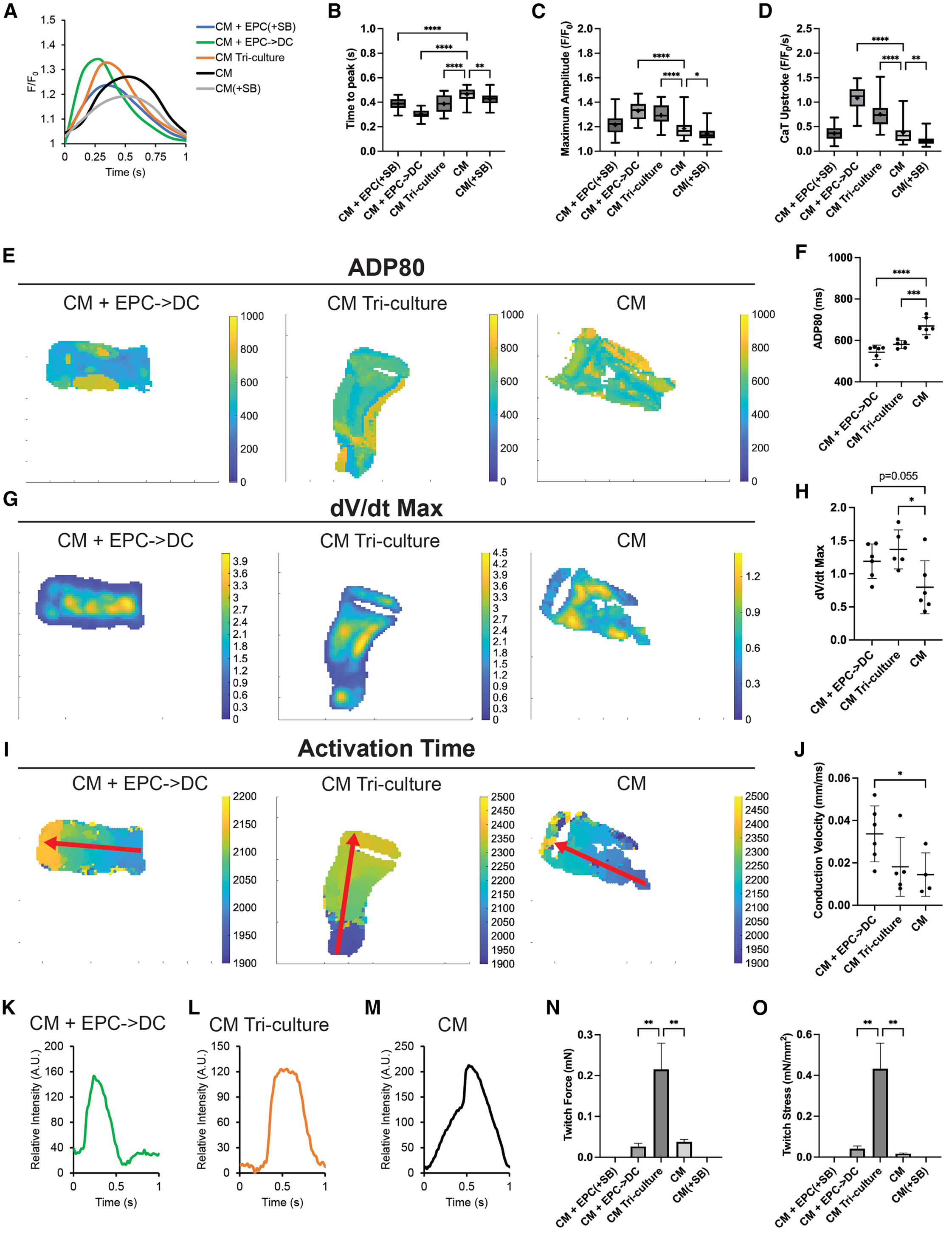

Figure 7. Epicardial cells enhance engineered heart tissue conduction while the CM tri-culture enhances electrochemical function and force

(A–D) (A) Representative EHT calcium transient (CaT) traces and quantified (B) time to peak, (C) maximum amplitude, and (D) CaT upstroke velocity.

(E and F) Voltage optical mapping for the CM + EPC->DC (left), CM Tri-culture (middle), and CM (right) control showing (E) representative APD80 maps and (F) quantified average APD80.

(legend continued on next page)

Further phenotypic evaluation of EHTs reveals enhanced tissue level morphologic maturation and restoration of adult sarcomeric isoforms in CM Tri-culture EHTs

To evaluate CM morphologic maturation in EHTs, tissue cryosections were stained with α-actinin and DAPI to assess CM sarcomere length (Figure S7A). As in 2D, the EPCs resulted in a slight but significant increase in sarcomere length that was present in both co-culture conditions (Figure S7B). The CM Tri-culture condition resulted in the largest increase in sarcomere length. Additionally, cellular alignment was evaluated in the EHTs using phalloidin. All co-cultures increased cellular alignment, but the largest increase was present in the CM Tri-culture: the phalloidin coherency is 0 when cells are randomly aligned and 1 when perfectly aligned (Figure S7C). To evaluate the level of myofibril adult/fetal isoform switching, RT-qPCR was used to determine the ratio of *MYH7* to *MYH6* expression (Figure S7D). We found a significant decrease in the *MYH7/MYH6* ratio in both the epicardial and epicardial-derived co-cultures. The presence of EPD-FBs ameliorated this effect and led to a slight increase in the *MYH7/MYH6* ratio.

Some key markers of electrophysiologic maturation were also assessed. The presence of *GJA1* was significantly increased in the CM + EPC(+SB) condition, possibly because the EPCs themselves were expressing large amounts of *GJA1* as determined by scRNA-seq (Figure S7E). There were no significant differences in the expression levels of *ATP2A2* and *KCNJ2* detected between any of the co-cultures with the CM controls (Figures S7F and S7G). Additionally, EPCs alone did not influence the bulk *RYR2* expressions but the CM Tri-culture conditions had a significant increase compared to the CM-only controls (Figure S7H). *PLN*, a regulator of SERCA2 or *ATP2A2*, was significantly decreased in both the epicardial co-cultures but this effect was ameliorated with the addition of fibroblasts in the CM Tri-culture condition (Figure S7J). To assess protein-level expression, we performed immunostaining for RYR2 and SERCA2 in EHT cryosections. We observed a significant increase in RYR2 expression in both conditions containing EPCs. In contrast, SERCA2 expression remained unchanged, consistent with the RT-qPCR results for *ATP2A2*. In summary, while EPCs spur a more fetal but electrochemically functional phenotype, the addition of EPD-FBs pushes the tissues one step further by sustaining electrochemical function and enhancing mechanical function.

DISCUSSION

Intrigued by embryonic development and the lack of knowledge with regard to CM maturation during this stage, we investigated murine hearts during E10, E12, and E17 and found that substantial amounts of CM maturation occur at this time. We then studied the effect of EPCs on ventricular CM *in vitro* and found that they simultaneously spurred a more electrochemically functional but fetal-like phenotype characterized by increases in proliferation and expression of fetal myofilament isoforms. Single-cell sequencing revealed the upregulation of multiple signaling pathways, including WNT, that are implicated in CM proliferation and compact myocardium specification during development. The increased electrochemical function was validated in 3D-EHTs. To mimic the multicellularity present during development, fully differentiated EPD-FBs were added to the EHTs in a CM Tri-culture. These tissues showed robust electrochemical maturation and force generation as well as increased adult myofilament isoforms. Taken together, we found that mimicking the multicellular progression of the fetal to late neonatal environment best supports hiPSC-CM functional maturation. These results leave us to speculate whether EPCs play a role in electrochemical maturation during *in vivo* development and, more importantly, whether embryonic development is the missing stage of hiPSC-CM maturation *in vitro*.

(G and H) (G) Representative optical mapping maximum upstroke velocity maps and (H) average maximum upstroke velocity.
(I) Optical mapping activation time maps showing signal propagation from where the point electrical stimulator was placed (end of the red arrow) to the other side of the EHT (point of the red arrow). The red arrow shows the distance and direction for which the conduction velocity was calculated across for these three EHTs.
(J) The quantified conduction velocity for all EHTs that showed an analyzable conduction pattern.
(K–M) Representative AP trace cropped to 1 s for the (K) CM + EPC->DC, (L) CM Tri-culture, and (M) CM conditions.
(N and O) (N) The quantified twitch force for EHTs with a detectable force and those with no detectable force (N.D) as well as the (O) twitch stress.
For (B and C), the data are represented as box and whisker plots with + marking the mean and $n = 12$ EHTs per condition from 3 independent experiments. For (F, H, and J), each dot represents the average across all pixels for one EHT, the center line is the mean, and the error bars represent the SD. For (F and H), $n = 6$ EHT across two independent experiments and for (J), $n = 6$ EHTs for CM + EPC->DC and CM Tr-culture and $n = 4$ for CM across two independent experiments. For (N and O), the bar graph represents the mean ± SEM and CM + EPC(+SB) ($n = 10$), CM + EPC->DC ($n = 10$), CM Tri-culture ($n = 8$), CM ($n = 12$), and CM(+SB) ($n = 12$) EHTs across 3 independent experiments were measured. $^*p < 0.05$, $^{**}p < 0.01$, $^{***}p < 0.001$, and $^{****}p < 0.0001$. See also Figure S7.

Here we show that EPCs are powerful mediators of hiPSC-CM electrochemical function. Enhanced calcium handling and AP parameters were seen in both 2D and 3D co-cultures. Some of the potential mediators for this increase were determined to be *RYR2* and *ATP2A2*, as they were significantly upregulated in the EPC(+SB) co-culture. *RYR2* is the dominant calcium channel that releases calcium from the sarcoplasmic reticulum. In a recent mouse model, a RYR2-depleted hearts showed functional deficits, decreased presence of T-tubules, and decreased morphologic maturation (CM sarcomere length, CM area, and elongation) (Guo et al., 2023). Therefore, the epicardial-induced *RYR2* expression could point to the mechanism for morphologic maturation increases as well as more mature calcium handling. Conversely, *ATP2A2* is responsible for bringing calcium back into the sarcoplasmic reticulum. This could be related to the significant increase in CaT downstroke velocity seen in the EPC(+SB) co-cultures. CACNB2 encodes the predominant β-subunit for cardiac L-type calcium channel; CM-specific knockout (KO) of this gene leads to reduced L-type calcium current and vascular dysfunction leading to embryonic lethality (Weissgerber et al., 2006). L-Type calcium currents are regulators of excitation-contraction coupling, and this could serve as another mediator for enhanced conduction seen in the epicardial co-cultures. When *RYR2* and *ATP2A2* were looked at in the EHTs, no changes were detected; this could be due to the loss of single-cell resolution in dense cultures and the challenge of finding a stable cardiac gene to normalize to in these highly heterogeneous cultures. In the epicardial-derived co-culture, *CASQ2* was significantly upregulated. This is the most abundant calcium-binding protein of the sarcoplasmic reticulum. This could be responsible for increased calcium handling seen in these conditions as genetic over-expression of *CASQ2* in hiPSC-CM has this effect (Liu et al., 2009).

Studies of CM electrophysiology at early time points in development are scarce. Multiple studies show that cardiac calcium handling and AP parameters shift between embryonic, post-natal, and adult phases of development but few encompass multiple points of embryonic development (Liu et al., 2002; Swift et al., 2020; Ziman et al., 2010). However, one study looking at mice hearts encompassed three embryonic time points as well as multiple post-natal time points (Peinkofer et al., 2016). Interestingly, the largest increase in AP amplitude, coupled with the largest decrease in MDP occurred between E9-10 and E12-14 of embryonic development. This corresponds to the developmental time point where EPCs have covered the heart and substantial amounts of EMT have occurred (Acharya et al., 2012; Vicente-Steijn et al., 2015; Von Gise et al., 2011). Though this is by no means evidence of epicardial-induced CM electrochemical maturation *in vivo,* it provides the rationale for future study. It also provides evidence that electrochemical maturation does occur during embryonic development and that by immediately pushing post-natal conditions in hiPSC-CM we might be missing this critical phase of maturation.

The parallel findings of increased maturation and proliferation in this study are intriguing as CM proliferation and maturation are described as dichotomous (Singh et al., 2023). This may indeed be the case *in vivo* as our murine data suggest CM proliferation does decrease as sarcomere length and cell area increase. It is important to note that the *in vitro* case is highly artificial in the way that it can exist without the epicardium since mouse models without the epicardium will not survive. It is also important to note that mouse embryonic CM maturation seen in this study occurs while CM proliferation is extremely high (~50%), meaning some amount of maturation does happen *in vivo* while CM are still in a highly proliferative state. The increases in proliferation we see in parallel with maturation could simply be a result of more accurately mimicking *in vivo* cardiac multicellularity. Additionally, the simultaneous increase in proliferation and calcium handling has been seen in previous *in vitro* hiPSC-CM and epicardial co-cultures (Tan et al., 2021). Taken together with the embryonic patch clamp data discussed previously, there is evidence to suggest that proliferation and aspects of CM maturation, such as electrochemical function, do occur simultaneously *in vitro*. Unlike the 2D co-cultures, the 3D EHTs containing EPCs did not have enhanced proliferation after 29–30 days of co-culture. This was the first study that looked at co-cultures for this long and it is very likely that an increase in CM proliferation would be temporal and eventually subside as the 3D environments promotes enhanced CM maturation. Future studies are needed to look at the temporal aspects of epicardial-induced CM proliferation to determine if a transient enhancement is present in the EHTs.

Our data suggest that mechanical maturation as well as the robust expression of adult myofilament isoforms likely occurs after CM-proliferation and some aspects of electrochemical maturation have occurred. We are the first to report that EPCs, before EMT, drive more fetal CM myofilament expression. Interestingly, epicardial-derived cells (in 2D) and EPD-FBs (in 3D) ameliorated this effect resulting in increased TNNI3 and MYL2 expression in 2D and an increase in the MYH7/MYH6 ratio in 3D. This finding fits with the progression seen in embryonic development where EPCs spur the expansion of CM, which leads to the formation of the dense compact myocardium in fetal development, and then myofilament isoform switching does not occur until postnatal maturation (Lu et al., 2022; Vicente-Steijn et al., 2015). The finding that myofilament maturation cannot occur while CM are proliferative

is not necessarily new as it is commonly postulated that the highly organized nature of mature sarcomeres provides a mechanical barrier for cell division as the complex myofilament bundles must disassemble for cytokinesis to occur. However, the uncoupling of sarcomere length and myofilament isoform switching in the proliferation maturation dichotomy is a new finding as we found the longest sarcomere length in the CM + EPC(+SB) condition. Our murine data support this uncoupling as substantial increases in murine sarcomere length occur *in vivo* while the CM are still highly proliferative at a time point in embryonic development where fetal myofilament isoform expression is still dominant (DeLaughter et al., 2016; Lu et al., 2022).

Our TFM data also suggest that myofilament isoform expression is not necessarily related to hiPSC-CM single-cell force generation. TFM reported no significant changes in single cell force generation even though fetal isoforms prevailed in the CM + EPC(+SB) co-culture. Further, sarcomere length seemed to be a better predictor of increased force generation as the only conditions with a significantly different force were the CM + EPC(+SB) and the CM(+SB) conditions, which had the highest and lowest sarcomere length, respectively. This finding is supported by another study where single hiPSC-CM contractility and APs were looked at in a reporter system allowing visualization of MYH6 and MYH7 (Weber et al., 2020). This study found that the myosin heavy chain isoform expression did not correlate to CM mechanical or electrophysiologic function (Weber et al., 2020). In our study, the most mature electrochemical function was seen in the population determined to have a more fetal myofilament composition (CM + EPC [+SB]). Taken together, our results beg the question of whether this "intermediate" stage of development should be achieved before post-natal conditions are imposed on the hiPSC-CM. If we push myofilament switching and reductions in CM proliferation too soon, will proper CM maturation ever be achieved? Here, we provide evidence that mimicking embryonic development can have a powerful impact on the maturation state of the CM. Future studies could impose additional post-natal conditions, such as electrical stimulation, increased afterload, and a switch to fatty acid/glucose depleted media after the imposition of embryonic developmental cues.

Here, we also report the first single-cell sequencing experiment of hiPSC-CM and EPCs in co-culture. We found that EPCs have enormous effects on the phenotype of the hiPSC-CM and vice versa. In addition to the shift in calcium handling and myofilament genes discussed previously, these results also pointed to multiple signaling cascades that were only upregulated in the epicardial co-cultures. Among these, the WNT signaling cascade is of interest because of the role it has been seen to play in spurring CM proliferation *in vivo* and *in vitro* (Buikema et al., 2020; Fan et al., 2018). WNT signaling was increased in both the vCM and the EPCs from the co-culture conditions. Additionally, CM proliferation seen *in vivo* is also coupled with the appearance of the compact myocardium, a portion of the myocardium lacking in epicardial KO mice models (Vicente-Steijn et al., 2015). Interestingly, small molecule WNT activation has also been used to induce expression of the compact myocardium markers *HEY2* and *FZD2* in hiPSC-CM (Funakoshi et al., 2021). These markers, along with increased CM proliferation, were both significantly increased here in the CM co-cultured with EPCs (Funakoshi et al., 2021). This indicates WNT as a potential mechanism for EPC-induced proliferation and compact myocardium specification. In development, the growth factor IGF2 has also been seen to mediate CM proliferation/ventricular compaction (Brade et al., 2011; Li et al., 2011). This mechanism could possibly converge on WNT signaling as well since IGF2 has been seen to activate *WNT5A* expression and the WNT signaling pathway in cancer cells (Belharazem et al., 2016). *WNT5A* is a DEG in that was upregulated in the vCM of our CM + EPC(+SB) co-cultures. This indicates that *WNT5A* could be explored as the potential mediator of IGF2 and WNT-induced CM proliferation perhaps as a more refined and less variable approach to promote CM proliferation, compact myocardium specification, and potentially even electrochemical functionality, than the use of EPC co-culture.

Taken together, this study is the first to describe the robust electrochemical maturation conferred by pure populations of EPCs. It also holds significant implications for the field of cardiac tissue engineering as it identifies the embryonic phase of CM maturation as a unique and perhaps critical component of hiPSC-CM maturation. As developmental biology was mimicked to determine the pathways involved in CM-specification to yield robust hiPSC-CM differentiation protocols, the temporal modulation of CM embryonic maturation cues followed by post-natal cues could be envisioned for the realization of a fully functional adult phenotype in hiPSC-CM.

METHODS

2D co-culture construction and characterization

Briefly, hiPSCs were maintained on Matrigel in mTesR1. The hiPSCs were differentiated into hiPSC-CM and EPCs using WNT modulation. After differentiation, the hiPSC-CM were seeded onto Matrigel; 24 h later, the EPCs were seeded on top of the hiPSC-CM at a ratio of 25% EPCs to 75% hiPSC-CM. The co-cultures were maintained with or without TGF-β inhibitors (SB) for 8 day, and then assessed via flow cytometry, for hiPSC-CM morphology, calcium

transients, patch clamp, TFM, TEM, and scRNA-seq. Detailed methods for the characterization of these co-cultures and the maintenance and differentiation of the hiPSCs that make up the co-cultures are described in the supplemental methods.

Engineered heart tissue construction and characterization

The EHTs are constructed by combining hiPSC-CM and EPCs or EPCs and EPD-FBs (CM Tri-culture) in media with fibrinogen and thrombin to form a fibrin gel. The EHTs are then maintained in media containing trypsin inhibitors for 29–31 days until they were assessed for force generation via a force transducer, calcium transients, protein expression via immunohistochemistry (IHC), and gene expression via RT-qPCR. The detailed methods for EHT construction and characterization are outlined in the supplemental methods.

Statistical analyses

All statistical analyses were done in PRISM using a one-way or two-way ANOVA and post-hoc Fisher least significant difference comparison of means. Significance was indicated as follows: ****$p \leq 0.0001$, ***$p \leq 0.001$, **$p \leq 0.01$, and *$p \leq 0.05$.

RESOURCE AVAILABILITY

Lead contact

The data supporting this manuscript can be found in the figures of the manuscript and supplemental materials or by request to the lead contact, Brenda M. Ogle (ogle@umn.edu).

Material availability

No new materials were generated in the making of this manuscript.

Data and code availability

The single-cell sequencing data are available on GEO: GSE293435. The original code generated for this manuscript is available on GitHub: Sanaz081/OM-Stem-Cell-Analysis. FAIR and CARE data management practices were followed.

ACKNOWLEDGMENTS

The authors would like to awknowledge our funding sources RO1 HL137204, RO1 HL160779, NSF GRFP 2019272039, and the University of Minnesota Doctoral Dissertation Fellowship. The authors would like to thank the University of Minnesota 3D Bioprinting Facility for use of the Mach-1 Micromechanical Tissue Tester; Caleb Vogt for assembly of the Mach-1 for EHT mounting and force measurements; the University of Minnesota Genomics Center (UMGC) for single-cell library preparation and sequencing; Noah Stanis for calcium transient MATLAB code; and the University of Minnesota Imaging Center (UIC) for instruction, housing, and maintenance of the Olympus FluoView IX2 Inverted Confocal Microscope.

AUTHOR CONTRIBUTIONS

S.E.G. designed the study, performed the experiments, analyzed the data, and wrote the manuscript. A.A.A., R.W., X.K., T.M.R., S.H., A.X., M.S., and A.A.T. performed experimentation and analysis. S.F.B., M.J., and N.C.M. performed analysis. B.N.S., S.D., P.W.A., E.G.T., and J.H.v.B. contributed to the interpretation of data and writing of the manuscript. B.M.O. contributed to experimental design, interpretation of data and writing of the manuscript.

DECLARATION OF INTERESTS

The authors declare no competing interests.

SUPPLEMENTAL INFORMATION

Supplemental information can be found online at https://doi.org/10.1016/j.stemcr.2025.102572.

Received: January 14, 2025
Revised: June 5, 2025
Accepted: June 6, 2025
Published: July 3, 2025

REFERENCES

Acharya, A., Baek, S.T., Huang, G., Eskiocak, B., Goetsch, S., Sung, C.Y., Banfi, S., Sauer, M.F., Olsen, G.S., Duffield, J.S., et al. (2012). The bHLH transcription factor Tcf21 is required for lineage-specific EMT of cardiac fibroblast progenitors. Development *139*, 2139–2149. https://doi.org/10.1242/dev.079970.

Arvanitis, D.A., Vafiadaki, E., Sanoudou, D., and Kranias, E.G. (2011). Histidine-rich calcium binding protein: The new regulator of sarcoplasmic reticulum calcium cycling. J. Mol. Cell. Cardiol. *50*, 43–49. https://doi.org/10.1016/j.yjmcc.2010.08.021.

Bao, X., Lian, X., Qian, T., Bhute, V.J., Han, T., and Palecek, S.P. (2017). Directed differentiation and long-term maintenance of epicardial cells derived from human pluripotent stem cells under fully defined conditions. Nat. Protoc. *12*, 1890–1900. https://doi.org/10.1038/nprot.2017.080.

Bargehr, J., Ong, L.P., Colzani, M., Davaapil, H., Hofsteen, P., Bhandari, S., Gambardella, L., Le Novère, N., Iyer, D., Sampaziotis, F., et al. (2019). Epicardial cells derived from human embryonic stem cells augment cardiomyocyte-driven heart regeneration. Nat. Biotechnol. *37*, 895–906. https://doi.org/10.1038/s41587-019-0197-9.

Beauchamp, P., Jackson, C.B., Ozhathil, L.C., Agarkova, I., Galindo, C.L., Sawyer, D.B., Suter, T.M., and Zuppinger, C. (2020). 3D Co-culture of hiPSC-Derived Cardiomyocytes With Cardiac Fibroblasts Improves Tissue-Like Features of Cardiac Spheroids. Front. Mol. Biosci. *7*, 14. https://doi.org/10.3389/fmolb.2020.00014.

Belharazem, D., Magdeburg, J., Berton, A.-K., Beissbarth, L., Sauer, C., Sticht, C., Marx, A., Hofheinz, R., Post, S., Kienle, P., and Ströbel, P. (2016). Carcinoma of the colon and rectum with deregulation of insulin-like growth factor 2 signaling: Clinical and molecular implications. J. Gastroenterol. *51*, 971–984. https://doi.org/10.1007/s00535-016-1181-5.

Bergmann, O., Zdunek, S., Felker, A., Salehpour, M., Alkass, K., Bernard, S., Sjostrom, S.L., Szewczykowska, M., Jackowska, T., dos Remedios, C., et al. (2015). Dynamics of Cell Generation and Turnover in the Human Heart. Cell *161*, 1566–1575. https://doi.org/10.1016/j.cell.2015.05.026.

Brade, T., Kumar, S., Cunningham, T.J., Chatzi, C., Zhao, X., Cavallero, S., Li, P., Sucov, H.M., Ruiz-Lozano, P., and Duester, G. (2011). Retinoic acid stimulates myocardial expansion by induction of hepatic erythropoietin which activates epicardial *Igf2*. Development *138*, 139–148. https://doi.org/10.1242/dev.054239.

Branco, M.A., Cotovio, J.P., Rodrigues, C.A.V., Vaz, S.H., Fernandes, T.G., Moreira, L.M., Cabral, J.M.S., and Diogo, M.M. (2019). Transcriptomic analysis of 3D Cardiac Differentiation of Human Induced Pluripotent Stem Cells Reveals Faster Cardiomyocyte Maturation Compared to 2D Culture. Sci. Rep. *9*, 9229. https://doi.org/10.1038/s41598-019-45047-9.

Buikema, J.W., Lee, S., Goodyer, W.R., Maas, R.G., Chirikian, O., Li, G., Miao, Y., Paige, S.L., Lee, D., Wu, H., et al. (2020). Wnt Activation and Reduced Cell-Cell Contact Synergistically Induce Massive Expansion of Functional Human iPSC-Derived Cardiomyocytes. Cell Stem Cell *27*, 50–63.e5. https://doi.org/10.1016/j.stem.2020.06.001.

Chen, G., Li, S., Karakikes, I., Ren, L., Chow, M.Z.Y., Chopra, A., Keung, W., Yan, B., Chan, C.W.Y., Costa, K.D., et al. (2015). Phospholamban as a Crucial Determinant of the Inotropic Response of Human Pluripotent Stem Cell–Derived Ventricular Cardiomyocytes and Engineered 3-Dimensional Tissue Constructs. Circ. Arrhythm. Electrophysiol. *8*, 193–202. https://doi.org/10.1161/CIRCEP.114.002049.

Chen, T.H.-P., Chang, T.-C., Kang, J.-O., Choudhary, B., Makita, T., Tran, C.M., Burch, J.B.E., Eid, H., and Sucov, H.M. (2002). Epicardial Induction of Fetal Cardiomyocyte Proliferation via a Retinoic Acid-Inducible Trophic Factor. Dev. Biol. *250*, 198–207. https://doi.org/10.1006/dbio.2002.0796.

Clemens, D.J., Ye, D., Wang, L., Kim, C.S.J., Zhou, W., Dotzler, S.M., Tester, D.J., Marty, I., Knollmann, B.C., and Ackerman, M.J. (2023). Cellular and electrophysiological characterization of triadin knockout syndrome using induced pluripotent stem cell-derived cardiomyocytes. Stem Cell Rep. *18*, 1075–1089. https://doi.org/10.1016/j.stemcr.2023.04.005.

Correia, C., Koshkin, A., Duarte, P., Hu, D., Carido, M., Sebastião, M.J., Gomes-Alves, P., Elliott, D.A., Domian, I.J., Teixeira, A.P., et al. (2018). 3D aggregate culture improves metabolic maturation of human pluripotent stem cell derived cardiomyocytes. Biotechnol. Bioeng. *115*, 630–644. https://doi.org/10.1002/bit.26504.

DeLaughter, D.M., Bick, A.G., Wakimoto, H., McKean, D., Gorham, J.M., Kathiriya, I.S., Hinson, J.T., Homsy, J., Gray, J., Pu, W., et al. (2016). Single-Cell Resolution of Temporal Gene Expression during Heart Development. Dev. Cell *39*, 480–490. https://doi.org/10.1016/j.devcel.2016.10.001.

Dorn, G.W., Vega, R.B., and Kelly, D.P. (2015). Mitochondrial biogenesis and dynamics in the developing and diseased heart. Genes Dev. *29*, 1981–1991. https://doi.org/10.1101/gad.269894.115.

Dunn, K.K., Reichardt, I.M., Simmons, A.D., Jin, G., Floy, M.E., Hoon, K.M., and Palecek, S.P. (2019). Coculture of Endothelial Cells with Human Pluripotent Stem Cell-Derived Cardiac Progenitors Reveals a Differentiation Stage-Specific Enhancement of Cardiomyocyte Maturation. Biotechnol. J. *14*, 1800725. https://doi.org/10.1002/biot.201800725.

Ergir, E., Oliver-De La Cruz, J., Fernandes, S., Cassani, M., Niro, F., Pereira-Sousa, D., Vrbský, J., Vinarský, V., Perestrelo, A.R., Debellis, D., et al. (2022). Generation and maturation of human iPSC-derived 3D organotypic cardiac microtissues in long-term culture. Sci. Rep. *12*, 17409. https://doi.org/10.1038/s41598-022-22225-w.

Fan, Y., Ho, B.X., Pang, J.K.S., Pek, N.M.Q., Hor, J.H., Ng, S.-Y., and Soh, B.-S. (2018). Wnt/β-catenin-mediated signaling re-activates proliferation of matured cardiomyocytes. Stem Cell Res. Ther. *9*, 338. https://doi.org/10.1186/s13287-018-1086-8.

Floy, M.E., Dunn, K.K., Mateyka, T.D., Reichardt, I.M., Steinberg, A. B., and Palecek, S.P. (2022). Direct coculture of human pluripotent stem cell-derived cardiac progenitor cells with epicardial cells induces cardiomyocyte proliferation and reduces sarcomere organization. J. Mol. Cell. Cardiol. *162*, 144–157. https://doi.org/10.1016/j.yjmcc.2021.09.009.

Funakoshi, S., Fernandes, I., Mastikhina, O., Wilkinson, D., Tran, T., Dhahri, W., Mazine, A., Yang, D., Burnett, B., Lee, J., et al. (2021). Generation of mature compact ventricular cardiomyocytes from human pluripotent stem cells. Nat. Commun. *12*, 3155. https://doi.org/10.1038/s41467-021-23329-z.

Garay, B.I., Givens, S., Abreu, P., Liu, M., Yücel, D., Baik, J., Stanis, N., Rothermel, T.M., Magli, A., Abrahante, J.E., et al. (2022). Dual inhibition of MAPK and PI3K/AKT pathways enhances maturation of human iPSC-derived cardiomyocytes. Stem Cell Rep. *17*, 2005–2022. https://doi.org/10.1016/j.stemcr.2022.07.003.

Giacomelli, E., Meraviglia, V., Campostrini, G., Cochrane, A., Cao, X., Van Helden, R.W.J., Krotenberg Garcia, A., Mircea, M., Kostidis, S., Davis, R.P., et al. (2020). Human-iPSC-Derived Cardiac Stromal Cells Enhance Maturation in 3D Cardiac Microtissues and Reveal Non-cardiomyocyte Contributions to Heart Disease. Cell Stem Cell *26*, 862–879.e11. https://doi.org/10.1016/j.stem.2020.05.004.

Goversen, B., Van Der Heyden, M.A.G., Van Veen, T.A.B., and De Boer, T.P. (2018). The immature electrophysiological phenotype of iPSC-CMs still hampers in vitro drug screening: Special focus on I K1. Pharmacol. Ther. *183*, 127–136. https://doi.org/10.1016/j.pharmthera.2017.10.001.

Grieskamp, T., Rudat, C., Lüdtke, T.H.-W., Norden, J., and Kispert, A. (2011). Notch Signaling Regulates Smooth Muscle Differentiation of Epicardium-Derived Cells. Circ. Res. *108*, 813–823. https://doi.org/10.1161/CIRCRESAHA.110.228809.

Guo, Y., Cao, Y., Jardin, B.D., Zhang, X., Zhou, P., Guatimosim, S., Lin, J., Chen, Z., Zhang, Y., Mazumdar, N., et al. (2023). Ryanodine receptor 2 (RYR2) dysfunction activates the unfolded protein response and perturbs cardiomyocyte maturation. Cardiovasc. Res. *119*, 221–235. https://doi.org/10.1093/cvr/cvac077.

Guo, Y., and Pu, W.T. (2020). Cardiomyocyte Maturation: New Phase in Development. Circ. Res. *126*, 1086–1106. https://doi.org/10.1161/CIRCRESAHA.119.315862.

Horikoshi, Y., Yan, Y., Terashvili, M., Wells, C., Horikoshi, H., Fujita, S., Bosnjak, Z.J., and Bai, X. (2019). Fatty Acid-Treated Induced Pluripotent Stem Cell-Derived Human Cardiomyocytes Exhibit

Adult Cardiomyocyte-Like Energy Metabolism Phenotypes. Cells *8*, 1095. https://doi.org/10.3390/cells8091095.

Kim, J.E., Kim, E.-M., Lee, H.-A., and Kim, K.-S. (2023). Effective derivation of ventricular cardiomyocytes from hPSCs using ascorbic acid-containing maturation medium. Anim. Cells Syst. *27*, 82–92. https://doi.org/10.1080/19768354.2023.2189932.

Kupfer, M.E., Lin, W.-H., Ravikumar, V., Qiu, K., Wang, L., Gao, L., Bhuiyan, D.B., Lenz, M., Ai, J., Mahutga, R.R., et al. (2020). In Situ Expansion, Differentiation, and Electromechanical Coupling of Human Cardiac Muscle in a 3D Bioprinted, Chambered Organoid. Circ. Res. *127*, 207–224. https://doi.org/10.1161/CIRCRESAHA.119.316155.

Lai, D., Liu, X., Forrai, A., Wolstein, O., Michalicek, J., Ahmed, I., Garratt, A.N., Birchmeier, C., Zhou, M., Hartley, L., et al. (2010). Neuregulin 1 Sustains the Gene Regulatory Network in Both Trabecular and Nontrabecular Myocardium. Circ. Res. *107*, 715–727. https://doi.org/10.1161/CIRCRESAHA.110.218693.

Lemoine, M.D., Mannhardt, I., Breckwoldt, K., Prondzynski, M., Flenner, F., Ulmer, B., Hirt, M.N., Neuber, C., Horváth, A., Kloth, B., et al. (2017). Human iPSC-derived cardiomyocytes cultured in 3D engineered heart tissue show physiological upstroke velocity and sodium current density. Sci. Rep. *7*, 5464. https://doi.org/10.1038/s41598-017-05600-w.

Leonard, A., Bertero, A., Powers, J.D., Beussman, K.M., Bhandari, S., Regnier, M., Murry, C.E., and Sniadecki, N.J. (2018). Afterload promotes maturation of human induced pluripotent stem cell derived cardiomyocytes in engineered heart tissues. J. Mol. Cell. Cardiol. *118*, 147–158. https://doi.org/10.1016/j.yjmcc.2018.03.016.

Li, G., Xu, A., Sim, S., Priest, J.R., Tian, X., Khan, T., Quertermous, T., Zhou, B., Tsao, P.S., Quake, S.R., and Wu, S.M. (2016). Transcriptomic Profiling Maps Anatomically Patterned Subpopulations among Single Embryonic Cardiac Cells. Dev. Cell *39*, 491–507. https://doi.org/10.1016/j.devcel.2016.10.014.

Li, P., Cavallero, S., Gu, Y., Chen, T.H.P., Hughes, J., Hassan, A.B., Brüning, J.C., Pashmforoush, M., and Sucov, H.M. (2011). IGF signaling directs ventricular cardiomyocyte proliferation during embryonic heart development. Development *138*, 1795–1805. https://doi.org/10.1242/dev.054338.

Lian, X., Hsiao, C., Wilson, G., Zhu, K., Hazeltine, L.B., Azarin, S. M., Raval, K.K., Zhang, J., Kamp, T.J., and Palecek, S.P. (2012). Robust cardiomyocyte differentiation from human pluripotent stem cells via temporal modulation of canonical Wnt signaling. Proc. Natl. Acad. Sci. USA *109*, E1848–E1857. https://doi.org/10.1073/pnas.1200250109.

Liu, J., Lieu, D.K., Siu, C.W., Fu, J.-D., Tse, H.-F., and Li, R.A. (2009). Facilitated maturation of Ca $^{2+}$ handling properties of human embryonic stem cell-derived cardiomyocytes by calsequestrin expression. Am. J. Physiol. Cell Physiol. *297*, C152–C159. https://doi.org/10.1152/ajpcell.00060.2009.

Liu, W., Yasui, K., Opthof, T., Ishiki, R., Lee, J.-K., Kamiya, K., Yokota, M., and Kodama, I. (2002). Developmental changes of Ca2+ handling in mouse ventricular cells from early embryo to adulthood. Life Sci. *71*, 1279–1292. https://doi.org/10.1016/S0024-3205(02)01826-X.

Lu, P., Wu, B., Feng, X., Cheng, W., Kitsis, R.N., and Zhou, B. (2022). Cardiac Myosin Heavy Chain Reporter Mice to Study Heart Development and Disease. Circ. Res. *131*, 364–366. https://doi.org/10.1161/CIRCRESAHA.122.321461.

Marchianò, S., Bertero, A., and Murry, C.E. (2019). Learn from Your Elders: Developmental Biology Lessons to Guide Maturation of Stem Cell-Derived Cardiomyocytes. Pediatr. Cardiol. *40*, 1367–1387. https://doi.org/10.1007/s00246-019-02165-5.

Mills, R.J., Titmarsh, D.M., Koenig, X., Parker, B.L., Ryall, J.G., Quaife-Ryan, G.A., Voges, H.K., Hodson, M.P., Ferguson, C., Drowley, L., et al. (2017). Functional screening in human cardiac organoids reveals a metabolic mechanism for cardiomyocyte cell cycle arrest. Proc. Natl. Acad. Sci. USA *114*, E8372–E8381. https://doi.org/10.1073/pnas.1707316114.

Peinkofer, G., Burkert, K., Urban, K., Krausgrill, B., Hescheler, J., Saric, T., and Halbach, M. (2016). From Early Embryonic to Adult Stage: Comparative Study of Action Potentials of Native and Pluripotent Stem Cell-Derived Cardiomyocytes. Stem Cells Dev. *25*, 1397–1406. https://doi.org/10.1089/scd.2016.0073.

Ronaldson-Bouchard, K., Ma, S.P., Yeager, K., Chen, T., Song, L., Sirabella, D., Morikawa, K., Teles, D., Yazawa, M., and Vunjak-Novakovic, G. (2018). Advanced maturation of human cardiac tissue grown from pluripotent stem cells. Nature *556*, 239–243. https://doi.org/10.1038/s41586-018-0016-3.

Rudat, C., and Kispert, A. (2012). Wt1 and Epicardial Fate Mapping. Circ. Res. *111*, 165–169. https://doi.org/10.1161/CIRCRESAHA.112.273946.

Salameh, S., Ogueri, V., and Posnack, N.G. (2023). Adapting to a new environment: Postnatal maturation of the human cardiomyocyte. J. Physiol. *601*, 2593–2619. https://doi.org/10.1113/JP283792.

Singh, B.N., Yucel, D., Garay, B.I., Tolkacheva, E.G., Kyba, M., Perlingeiro, R.C.R., Van Berlo, J.H., and Ogle, B.M. (2023). Proliferation and Maturation: Janus and the Art of Cardiac Tissue Engineering. Circ. Res. *132*, 519–540. https://doi.org/10.1161/CIRCRESAHA.122.321770.

Sonnenblick, E.H., Ross, J., Covell, J.W., Spotnitz, H.M., and Spiro, D. (1967). The Ultrastructure of the Heart in Systole and Diastole: >Changes In Sarcomere Length. Circ. Res. *21*, 423–431. https://doi.org/10.1161/01.RES.21.4.423.

Swift, L.M., Burke, M., Guerrelli, D., Reilly, M., Ramadan, M., McCullough, D., Prudencio, T., Mulvany, C., Chaluvadi, A., Jaimes, R., and Posnack, N.G. (2020). Age-dependent changes in electrophysiology and calcium handling: Implications for pediatric cardiac research. Am. J. Physiol. Heart Circ. Physiol. *318*, H354–H365. https://doi.org/10.1152/ajpheart.00521.2019.

Tan, J.J., Guyette, J.P., Miki, K., Xiao, L., Kaur, G., Wu, T., Zhu, L., Hansen, K.J., Ling, K.-H., Milan, D.J., and Ott, H.C. (2021). Human iPS-derived pre-epicardial cells direct cardiomyocyte aggregation expansion and organization in vitro. Nat. Commun. *12*, 4997. https://doi.org/10.1038/s41467-021-24921-z.

Tian, X., Li, Y., He, L., Zhang, H., Huang, X., Liu, Q., Pu, W., Zhang, L., Li, Y., Zhao, H., et al. (2017). Identification of a hybrid myocardial zone in the mammalian heart after birth. Nat. Commun. *8*, 87. https://doi.org/10.1038/s41467-017-00118-1.

Tyser, R.C.V., and Srinivas, S. (2020). The First Heartbeat—Origin of Cardiac Contractile Activity. Cold Spring Harb. Perspect. Biol. *12*, a037135. https://doi.org/10.1101/cshperspect.a037135.

Umbarkar, P., Singh, A.P., Gupte, M., Verma, V.K., Galindo, C.L., Guo, Y., Zhang, Q., McNamara, J.W., Force, T., and Lal, H. (2019). Cardiomyocyte SMAD4-Dependent TGF-β Signaling is Essential to Maintain Adult Heart Homeostasis. JACC. Basic Transl. Sci. *4*, 41–53. https://doi.org/10.1016/j.jacbts.2018.10.003.

Vicente-Steijn, R., Scherptong, R.W.C., Kruithof, B.P.T., Duim, S.N., Goumans, M.J.T.H., Wisse, L.J., Zhou, B., Pu, W.T., Poelmann, R.E., Schalij, M.J., et al. (2015). Regional differences in WT-1 and Tcf21 expression during ventricular development: Implications for myocardial compaction. PLoS One *10*, e0136025. https://doi.org/10.1371/journal.pone.0136025.

Von Gise, A., Zhou, B., Honor, L.B., Ma, Q., Petryk, A., and Pu, W.T. (2011). WT1 regulates epicardial epithelial to mesenchymal transition through β-catenin and retinoic acid signaling pathways. Dev. Biol. *356*, 421–431. https://doi.org/10.1016/j.ydbio.2011.05.668.

Weber, N., Kowalski, K., Holler, T., Radocaj, A., Fischer, M., Thiemann, S., De La Roche, J., Schwanke, K., Piep, B., Peschel, N., et al. (2020). Advanced Single-Cell Mapping Reveals that in hESC Cardiomyocytes Contraction Kinetics and Action Potential Are Independent of Myosin Isoform. Stem Cell Rep. *14*, 788–802. https://doi.org/10.1016/j.stemcr.2020.03.015.

Weissgerber, P., Held, B., Bloch, W., Kaestner, L., Chien, K.R., Fleischmann, B.K., Lipp, P., Flockerzi, V., and Freichel, M. (2006). Reduced Cardiac L-Type Ca^{2+} Current in $Ca_v\beta_2^{-/-}$ Embryos Impairs Cardiac Development and Contraction With Secondary Defects in Vascular Maturation. Circ. Res. *99*, 749–757. https://doi.org/10.1161/01.RES.0000243978.15182.c1.

Wheelwright, M., Win, Z., Mikkila, J.L., Amen, K.Y., Alford, P.W., and Metzger, J.M. (2018). Investigation of human iPSC-derived cardiac myocyte functional maturation by single cell traction force microscopy. PLoS One *13*, e0194909. https://doi.org/10.1371/journal.pone.0194909.

Wu, S.-P., Dong, X.-R., Regan, J.N., Su, C., and Majesky, M.W. (2013). Tbx18 regulates development of the epicardium and coronary vessels. Dev. Biol. *383*, 307–320. https://doi.org/10.1016/j.ydbio.2013.08.019.

Zamora, M., Männer, J., and Ruiz-Lozano, P. (2007). Epicardium-derived progenitor cells require β-catenin for coronary artery formation. Proc. Natl. Acad. Sci. USA *104*, 18109–18114. https://doi.org/10.1073/pnas.0702415104.

Ziman, A.P., Gómez-Viquez, N.L., Bloch, R.J., and Lederer, W.J. (2010). Excitation–contraction coupling changes during postnatal cardiac development. J. Mol. Cell. Cardiol. *48*, 379–386. https://doi.org/10.1016/j.yjmcc.2009.09.016.

Stem Cell Reports, Volume *20*

Supplemental Information

Developmental cues from epicardial cells simultaneously promote cardiomyocyte proliferation and electrochemical maturation

Sophie E. Givens, Abygail A. Andebrhan, Ruchen Wang, Xiangzhen Kong, Taylor M. Rothermel, Sanaz Hosseini, An Xie, Mohammad Shameem, Andrea A. Torniainen, Somayeh Ebrahimi-Barough, Samuel F. Boland, Maya Johnson, Natalia Calixto Mancipe, Bhairab N. Singh, Samuel Dudley, Patrick W. Alford, Elena G. Tolkacheva, Jop H. van Berlo, and Brenda M. Ogle

Supplemental Materials: Developmental cues from epicardial cells simultaneously promote cardiomyocyte proliferation and electrochemical maturation

Sophie E. Givens[1], Abygail A. Andebrhan[1], Ruchen Wang[2], Xiangzhen Kong[1], Taylor M. Rothermel[1], Sanaz Hosseini[3], An Xie[2], Mohammad Shameem[1,7], Andrea A. Torniainen[2], Somayeh Ebrahimi-Barough[1], Samuel F. Boland[1], Maya Johnson[1], Natalia Calixto Mancipe[8], Bhairab N. Singh[1,4,7], Samuel Dudley[2], Patrick W. Alford[1], Elena G. Tolkacheva[1,2,3,6], Jop H. van Berlo[2,4], Brenda M. Ogle[1,4,5,6]

[1]Biomedical Engineering, University of Minnesota, Minneapolis, MN, USA
[2]Lillehei Heart Institute (LHI), Department of Medicine, University of Minnesota, Minneapolis, MN, USA
[3]Electrical Engineering, University of Minnesota, Minneapolis, MN, USA
[4]Stem Cell Institute, University of Minnesota, Minneapolis, MN, USA
[5]Department of Pediatrics, University of Minnesota, Minneapolis, MN, USA
[6]Institute of Engineering in Medicine, University of Minnesota, MN, USA
[7]Department of Rehabilitation Medicine, University of Minnesota, MN, USA
[8]Minnesota Supercomputing Institute, University of Minnesota, MN, USA

Supplemental Figures and Legends

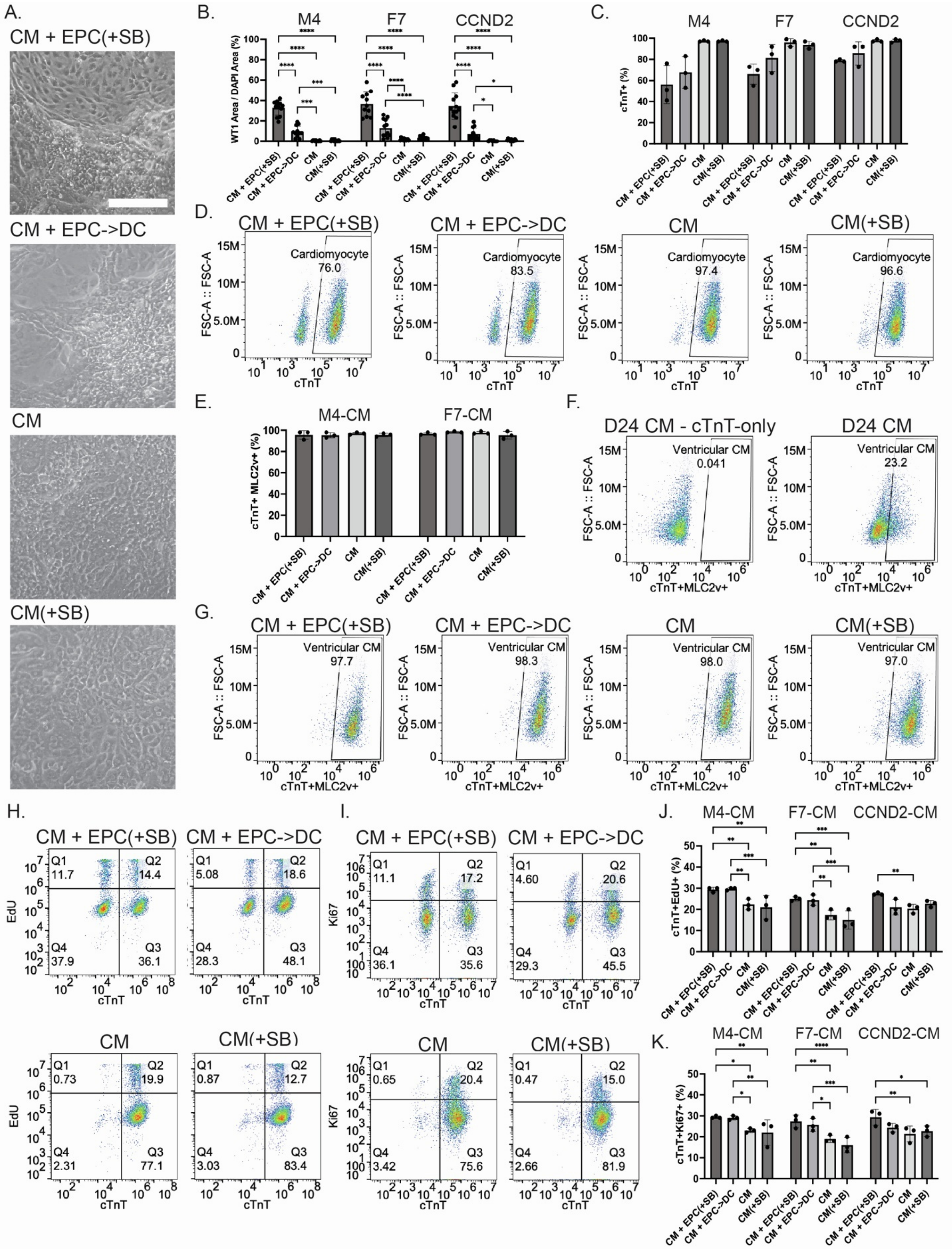

Figure S1 Co-culture composition and proliferation by line, related to Figure 2.

A) Brightfield images of all co-culture conditions at D35. Scale 50 µm. B) Quantification of WT1 Area / DAPI area indicating the percentage of epicardial cells in each co-culture condition broken down by line. C) Quantification of the percentage cTnT+ population from flow cytometry data broken down by lines. D) Representative flow cytometry data for cTnT. E) Quantification of the cTnT+MLC2v+ population for both lines quantified. F) Flow cytometry controls showing the cTnT+ and MLC2v on the x-axis with the day 24 CM stained for cTnT only (left) and D24 CM before treatment with ascorbic acid (right). G) Representative flow cytometry chart of the cTnT+MLC2v+ population for each co-culture condition at the experimental endpoint. H) Representative flow cytometry plots for each co-culture condition with cTnT on the x-axis and EdU on the y-axis as well as another set with I) cTnT on the x-axis and Ki67 on the y-axis. J) Quantification of flow cytometry for cardiomyocyte proliferation showing the percent cTnT+EdU+ population as well as K) the percent cTnT+Ki67+ population. (B-C), (E) and (J-K) Depict bar graphs and error bars representing the mean ± STDEV for n = 3 independent experiments for each condition. $^*p < 0.05$, $^{**}p < 0.01$, $^{***}p < 0.001$, and $^{****}p < 0.0001$ for (B).

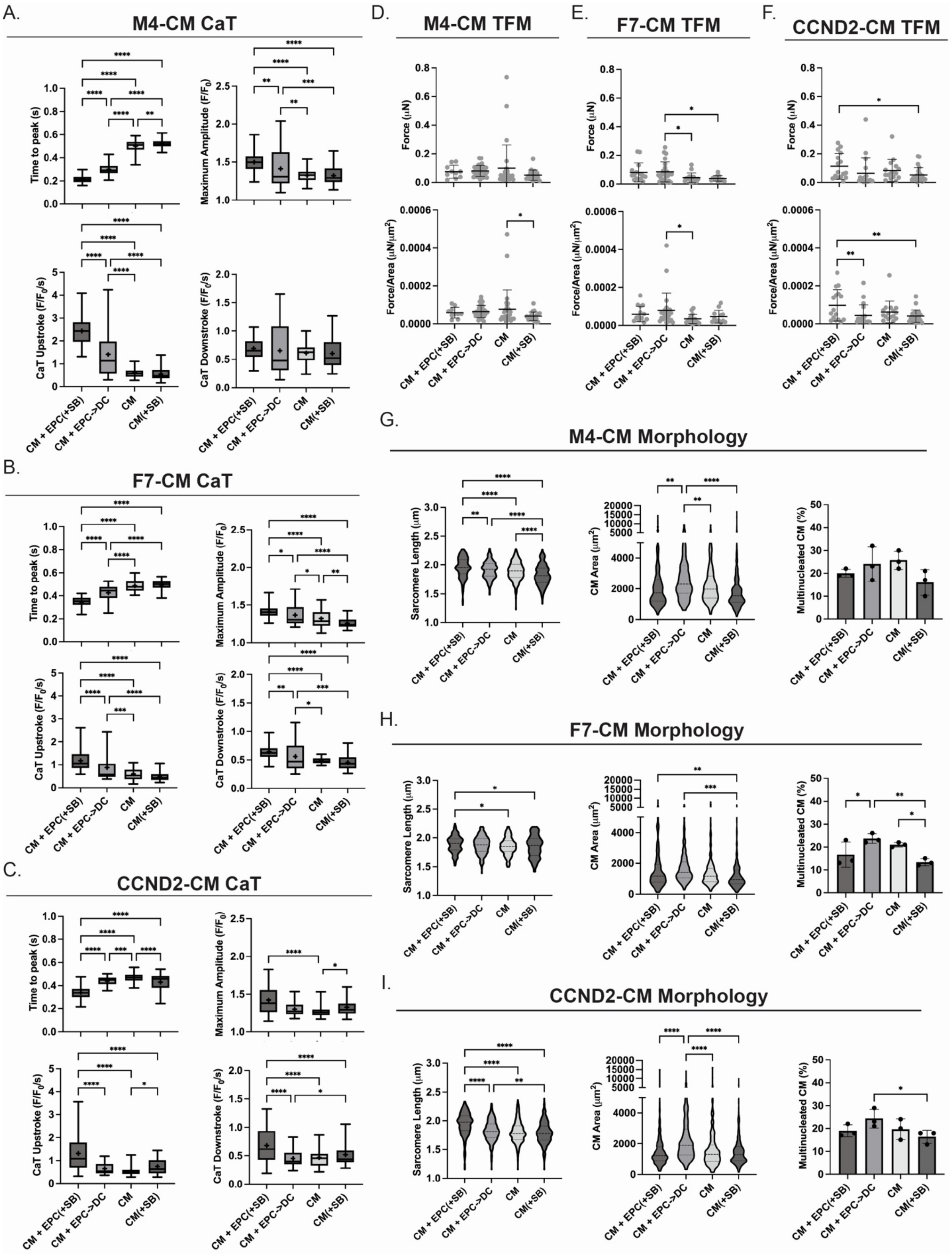
A.
M4-CM CaT
Time to peak (s)
Maximum Amplitude (F/F$_0$)
CaT Upstroke (F/F$_0$/s)
CaT Downstroke (F/F$_0$/s)
CM + EPC(+SB)
CM + EPC->DC
CM
CM(+SB)
B.
F7-CM CaT
C.
CCND2-CM CaT
D.
M4-CM TFM
E.
F7-CM TFM
F.
CCND2-CM TFM
Force (μN)
Force/Area (μN/μm^2)
G.
M4-CM Morphology
Sarcomere Length (μm)
CM Area (μm^2)
Multinucleated CM (%)
H.
F7-CM Morphology
I.
CCND2-CM Morphology

Figure S2. 2D Calcium transients, traction force microscopy, and CM morphology by hiPSC-line, related to Figures 3 and 4.

Quantification of the calcium transient parameters time to peak, maximum amplitude, upstroke, and downstroke velocity under 1 Hz electrical stimulation broken down for the A) M4 B) F7, and C) CCND2 hiPSC-lines. The traction force microscopy for D) M4-CM, E) F7-CM, and F) CCND2-CM showing the force (top) and the force normalized by cell area (bottom) for each co-culture condition. The morphologic assessment of CM replated onto glass slides at D35 and fixed in diastole 24hrs later showing quantification of CM sarcomere length (left), area (middle), and the percent multinucleated (right) for G) M4-CM, H) F7-CM and I) CCND2-CM. For (A-C) each graph is a box and whisker plot where the (+) represents the mean and the data represents three fields of view per well from three wells per replicate and n = 3 independent experiments per line. For (D-F) each data point represents one cardiomyocyte, and the center line and error bars represent the mean ± STDEV across n = 3 independent experiments. For (D) CM + EPC(+SB) (n = 10), CM + EPC->DC (n = 31), CM (n = 28) and CM(+SB) (n = 19). For (E) CM + EPC(+SB) (n = 15), CM + EPC->DC (n = 24), CM (n = 16) and CM(+SB) (n = 12) and for (F) CM + EPC(+SB) (n = 16), CM + EPC->DC (n = 19), CM (n = 16) and CM(+SB) (n = 23). For (G-I) the sarcomere length and area violin plot center dashed line represents the median and outer dashed lines represent upper and lower quartiles. The multinucleated percent bar graph represented the mean ± STDEV where each data point is the average percent of multinucleated CM across three wells and 5 fields of view per well for each independent experimental replicate with n = 3 independent experimental replicates per condition. In (G) for sarcomere length CM + EPC(+SB) (n = 276), CM + EPC->DC (n = 286), CM (n = 272) and CM(+SB) (n = 224) and for CM area CM + EPC(+SB) (n = 275), CM + EPC->DC (n = 280), CM (n = 270) and CM(+SB) (n = 224). In (H) for sarcomere length CM + EPC(+SB) (n = 202), CM + EPC->DC (n = 265), CM (n = 229) and CM(+SB) (n = 181) and for CM area CM + EPC(+SB) (n = 292), CM + EPC->DC (n = 192), CM (n = 263) and CM(+SB) (n = 284). In (I) for sarcomere length CM + EPC(+SB) (n = 410), CM + EPC->DC (n = 393), CM (n = 407) and CM(+SB) (n = 409) and for CM area CM + EPC(+SB) (n = 416), CM + EPC->DC (n = 388), CM (n = 503) and CM(+SB) (n = 413). *p < 0.05, **p < 0.01, ***p < 0.001, and ****p < 0.0001.

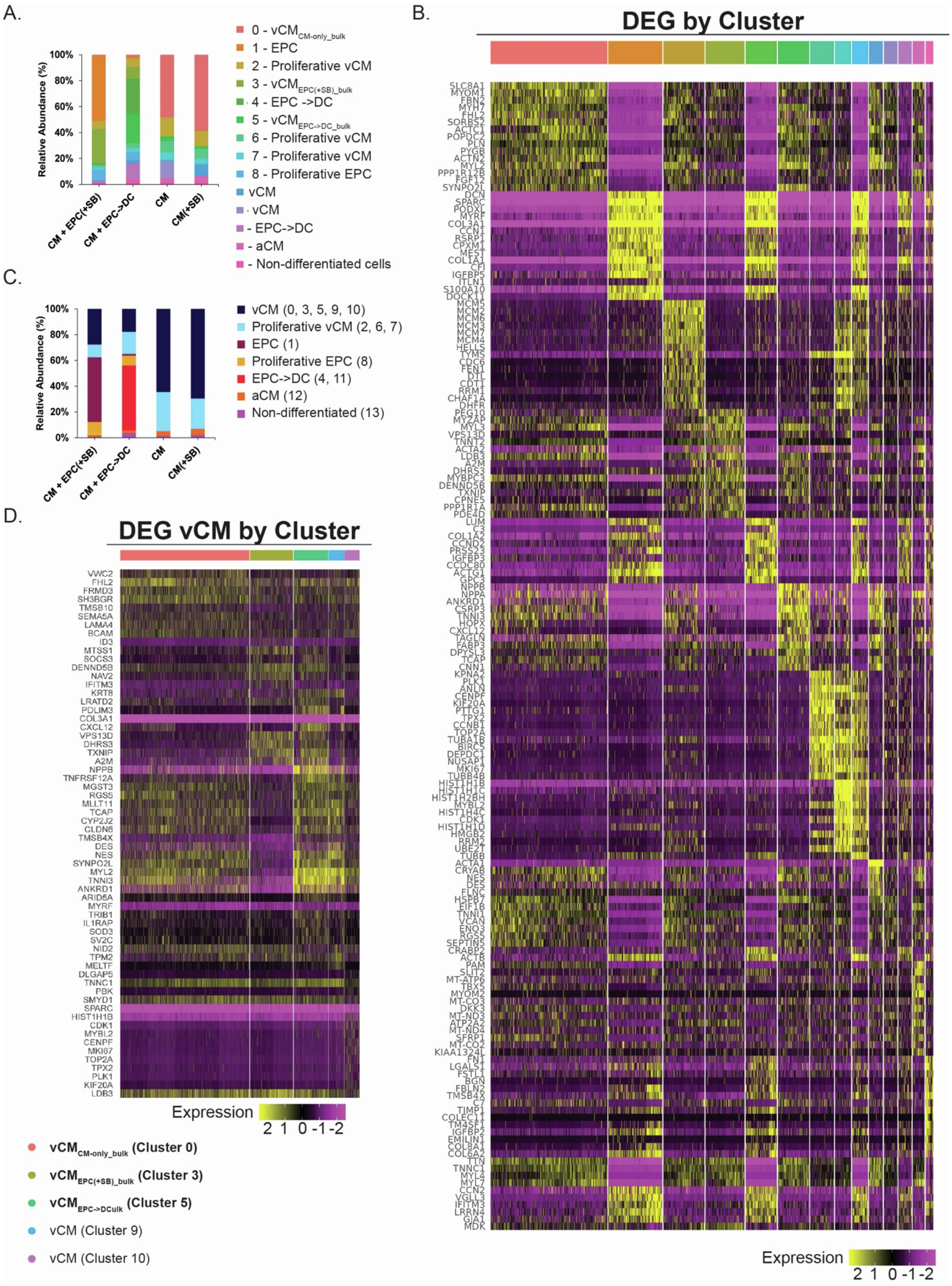
A.
B.
C.
D.
Relative Abundance (%)
100%
80%
60%
40%
20%
0%
CM + EPC(+SB)
CM + EPC->DC
CM
CM(+SB)
0 - vCM$_{CM\text{-only_bulk}}$
1 - EPC
2 - Proliferative vCM
3 - vCM$_{EPC(+SB)_bulk}$
4 - EPC ->DC
5 - vCM$_{EPC\text{->}DC_bulk}$
6 - Proliferative vCM
7 - Proliferative vCM
8 - Proliferative EPC
vCM
vCM
- EPC->DC
- aCM
- Non-differentiated cells
vCM (0, 3, 5, 9, 10)
Proliferative vCM (2, 6, 7)
EPC (1)
Proliferative EPC (8)
EPC->DC (4, 11)
aCM (12)
Non-differentiated (13)
DEG by Cluster
DEG vCM by Cluster
Expression
2 1 0 -1 -2
vCM$_{CM\text{-only_bulk}}$ (Cluster 0)
vCM$_{EPC(+SB)_bulk}$ (Cluster 3)
vCM$_{EPC\text{->}DCulk}$ (Cluster 5)
vCM (Cluster 9)
vCM (Cluster 10)
Expression
2 1 0 -1 -2

Figure S3. Single-cell RNAseq clustering of CM co-cultures, related to Figure 5.

A) Graph showing the proportions of cells from each condition in each cluster. B) A heat map of the top 15 differentially expressed genes (DEG) for each cluster. C) Graph showing the proportion of cells from each condition categorized by the 7 main labels 1) vCM (Cluster 0, 3, 5, 9 and 10), proliferative vCM (Cluster 2, 6 and 7), EPC (Cluster 1), EPC->DC (Cluster 4 and 11), aCM (Cluster 12) and non-differentiated cells (Cluster 13). D) A heat map of the top 15 differentially expressed genes between the 5 vCM clusters (Cluster 0, 3, 5, 9, and 10).

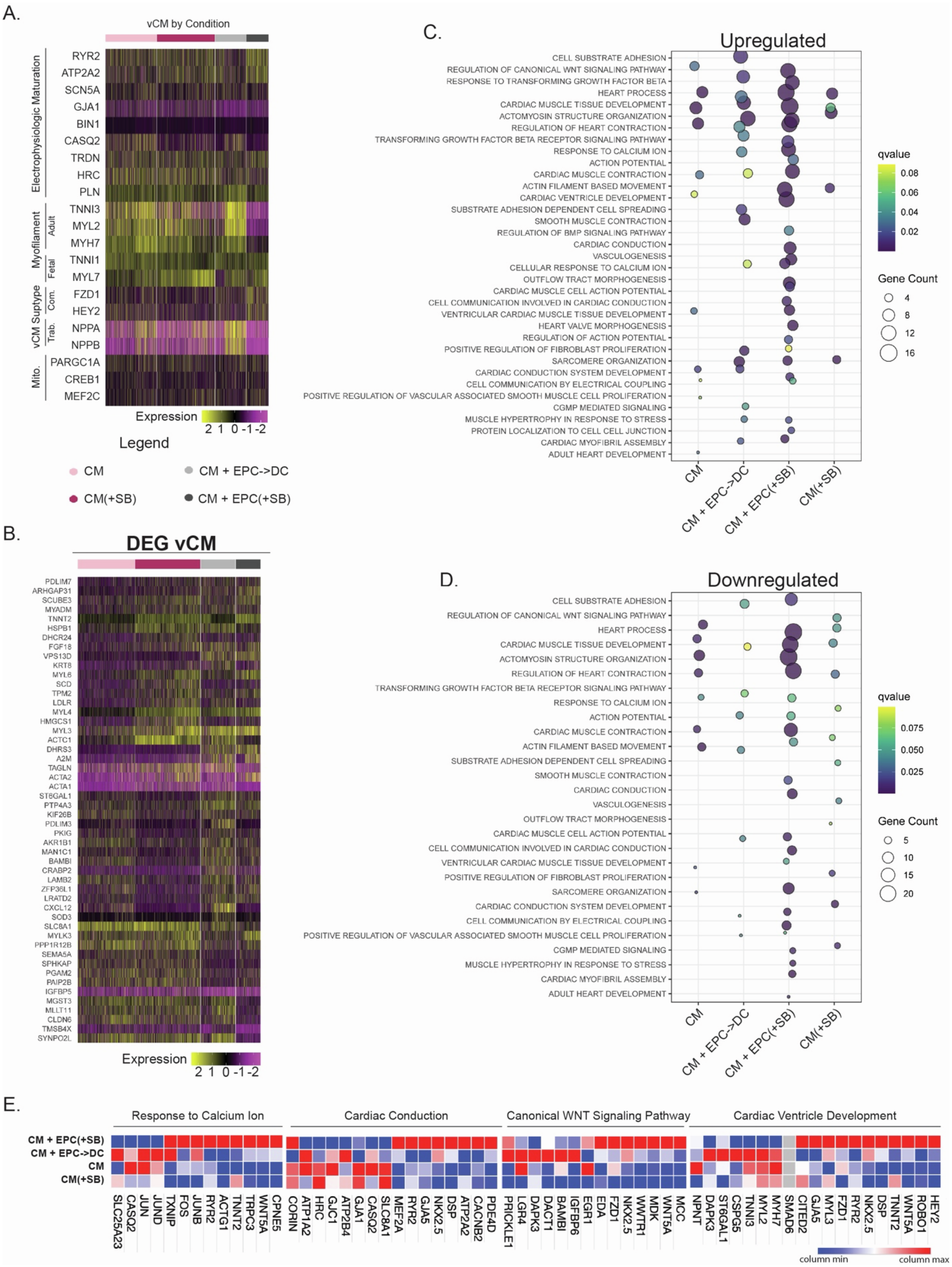
A.
vCM by Condition
Electrophysiologic Maturation
RYR2
ATP2A2
SCN5A
GJA1
BIN1
CASQ2
TRDN
HRC
PLN
Myofilament
Adult
TNNI3
MYL2
MYH7
Fetal
TNNI1
MYL7
vCM Suptype
Com.
FZD1
HEY2
Trab.
NPPA
NPPB
Mito.
PARGC1A
CREB1
MEF2C
Expression
2 1 0 -1 -2
Legend
CM
CM + EPC->DC
CM(+SB)
CM + EPC(+SB)
B.
DEG vCM
PDLIM7
ARHGAP31
SCUBE3
MYADM
TNNT2
HSPB1
DHCR24
FGF18
VPS13D
KRT8
MYL6
SCD
TPM2
LDLR
MYL4
HMGCS1
MYL3
ACTC1
DHRS3
A2M
TAGLN
ACTA2
ACTA1
ST6GAL1
PTP4A3
KIF26B
PDLIM3
PKIG
AKR1B1
MAN1C1
BAMBI
CRABP2
LAMB2
ZFP36L1
LRATD2
CXCL12
SOD3
SLC8A1
MYLK3
PPP1R12B
SEMA5A
SPHKAP
PGAM2
PAIP2B
IGFBP5
MGST3
MLLT11
CLDN6
TMSB4X
SYNPO2L
Expression
2 1 0 -1 -2
C.
Upregulated
CELL SUBSTRATE ADHESION
REGULATION OF CANONICAL WNT SIGNALING PATHWAY
RESPONSE TO TRANSFORMING GROWTH FACTOR BETA
HEART PROCESS
CARDIAC MUSCLE TISSUE DEVELOPMENT
ACTOMYOSIN STRUCTURE ORGANIZATION
REGULATION OF HEART CONTRACTION
TRANSFORMING GROWTH FACTOR BETA RECEPTOR SIGNALING PATHWAY
RESPONSE TO CALCIUM ION
ACTION POTENTIAL
CARDIAC MUSCLE CONTRACTION
ACTIN FILAMENT BASED MOVEMENT
CARDIAC VENTRICLE DEVELOPMENT
SUBSTRATE ADHESION DEPENDENT CELL SPREADING
SMOOTH MUSCLE CONTRACTION
REGULATION OF BMP SIGNALING PATHWAY
CARDIAC CONDUCTION
VASCULOGENESIS
CELLULAR RESPONSE TO CALCIUM ION
OUTFLOW TRACT MORPHOGENESIS
CARDIAC MUSCLE CELL ACTION POTENTIAL
CELL COMMUNICATION INVOLVED IN CARDIAC CONDUCTION
VENTRICULAR CARDIAC MUSCLE TISSUE DEVELOPMENT
HEART VALVE MORPHOGENESIS
REGULATION OF ACTION POTENTIAL
POSITIVE REGULATION OF FIBROBLAST PROLIFERATION
SARCOMERE ORGANIZATION
CARDIAC CONDUCTION SYSTEM DEVELOPMENT
CELL COMMUNICATION BY ELECTRICAL COUPLING
POSITIVE REGULATION OF VASCULAR ASSOCIATED SMOOTH MUSCLE CELL PROLIFERATION
CGMP MEDIATED SIGNALING
MUSCLE HYPERTROPHY IN RESPONSE TO STRESS
PROTEIN LOCALIZATION TO CELL CELL JUNCTION
CARDIAC MYOFIBRIL ASSEMBLY
ADULT HEART DEVELOPMENT
CM
CM + EPC->DC
CM + EPC(+SB)
CM(+SB)
qvalue
0.08
0.06
0.04
0.02
Gene Count
4
8
12
16
D.
Downregulated
CELL SUBSTRATE ADHESION
REGULATION OF CANONICAL WNT SIGNALING PATHWAY
HEART PROCESS
CARDIAC MUSCLE TISSUE DEVELOPMENT
ACTOMYOSIN STRUCTURE ORGANIZATION
REGULATION OF HEART CONTRACTION
TRANSFORMING GROWTH FACTOR BETA RECEPTOR SIGNALING PATHWAY
RESPONSE TO CALCIUM ION
ACTION POTENTIAL
CARDIAC MUSCLE CONTRACTION
ACTIN FILAMENT BASED MOVEMENT
SUBSTRATE ADHESION DEPENDENT CELL SPREADING
SMOOTH MUSCLE CONTRACTION
CARDIAC CONDUCTION
VASCULOGENESIS
OUTFLOW TRACT MORPHOGENESIS
CARDIAC MUSCLE CELL ACTION POTENTIAL
CELL COMMUNICATION INVOLVED IN CARDIAC CONDUCTION
VENTRICULAR CARDIAC MUSCLE TISSUE DEVELOPMENT
POSITIVE REGULATION OF FIBROBLAST PROLIFERATION
SARCOMERE ORGANIZATION
CARDIAC CONDUCTION SYSTEM DEVELOPMENT
CELL COMMUNICATION BY ELECTRICAL COUPLING
POSITIVE REGULATION OF VASCULAR ASSOCIATED SMOOTH MUSCLE CELL PROLIFERATION
CGMP MEDIATED SIGNALING
MUSCLE HYPERTROPHY IN RESPONSE TO STRESS
CARDIAC MYOFIBRIL ASSEMBLY
ADULT HEART DEVELOPMENT
CM
CM + EPC->DC
CM + EPC(+SB)
CM(+SB)
qvalue
0.075
0.050
0.025
Gene Count
5
10
15
20
E.
Response to Calcium Ion
Cardiac Conduction
Canonical WNT Signaling Pathway
Cardiac Ventricle Development
CM + EPC(+SB)
CM + EPC->DC
CM
CM(+SB)
SLC25A23 CASQ2 JUN JUND TXNIP FOS JUNB RYR2 ACTG1 TNNT2 TRPC3 WNT5A CPNE5
CORIN ATP1A2 HRC GJC1 ATP2B4 GJA1 CASQ2 SLC8A1 MEF2A RYR2 GJA5 NKX2.5 DSP ATP2A2 CACNB2 PDE4D
PRICKLE1 LGR4 DAPK3 DACT1 BAMBI IGFBP6 EGR1 EDA FZD1 NKX2.5 WWTR1 MDK WNT5A MCC
NPNT DAPK3 ST6GAL1 CSPG5 TNNI3 MYL2 MYH7 SMAD6 CITED2 GJA5 MYL3 FZD1 RYR2 NKX2.5 DSP TNNT2 WNT5A ROBO1 HEY2
column min
column max

Figure S4. Pooled vCM by condition DEG and ORA, related to Figure 5.

A) A heat map of cardiac genes of interest for all the vCM broken down by condition. B) Heat map of the top 15 differentially expressed genes between the vCM broken down by condition. Dot plots showing some of the ORA biological processes (BP) pathways that were significantly C) upregulated or D) downregulated in the vCM by conditions. E) Heat map of some of the differentially expressed genes contributing to enrichment in four GO BP pathways 1) Cardiac Ventricle Development, 2) Regulation of Canonical WNT Signaling Pathway, 3) Cardiac Conduction, and 4) Response to Calcium Ions.

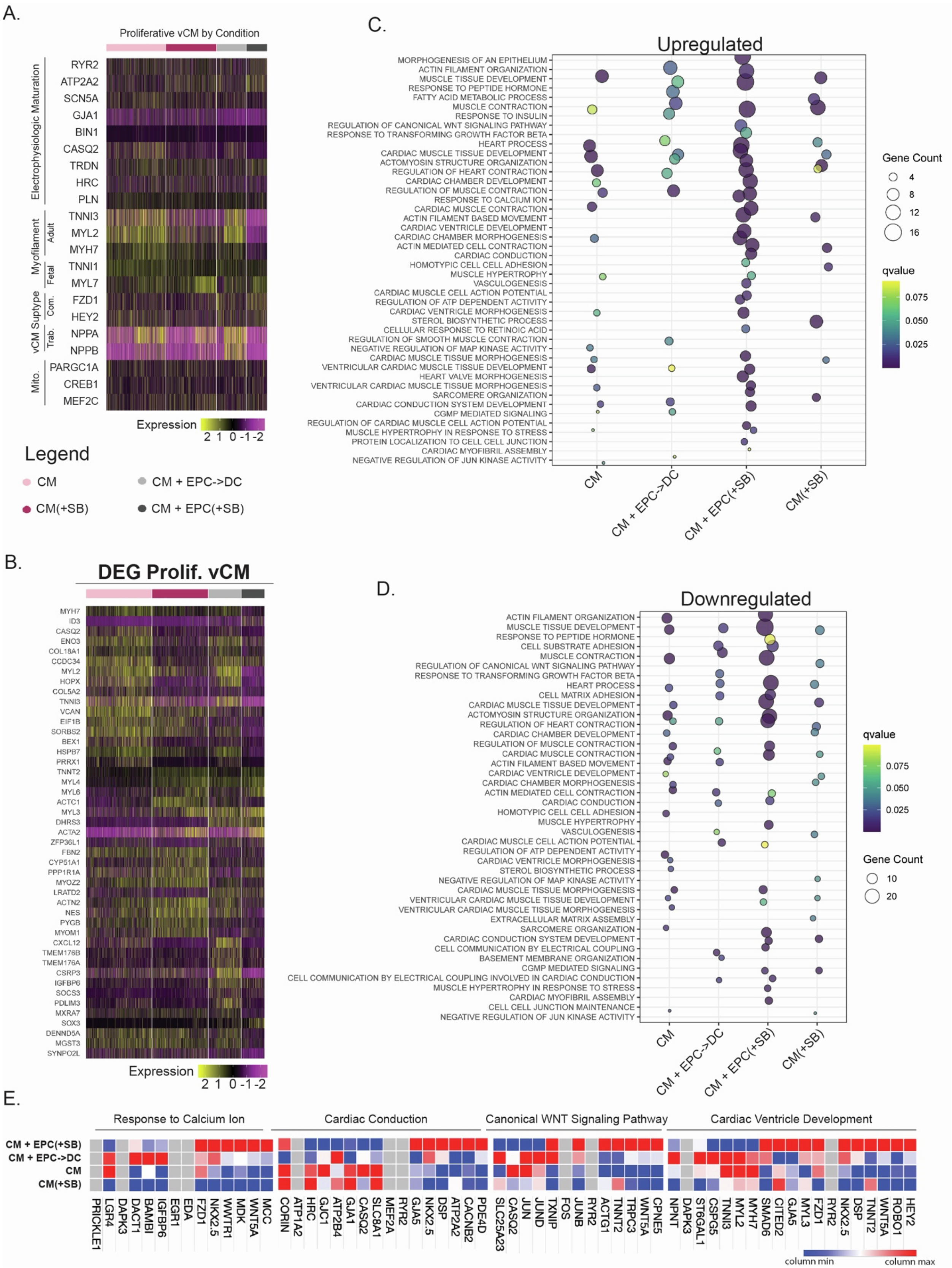
A.
Proliferative vCM by Condition
Electrophysiologic Maturation
RYR2
ATP2A2
SCN5A
GJA1
BIN1
CASQ2
TRDN
HRC
PLN
Myofilament
Adult
TNNI3
MYL2
MYH7
Fetal
TNNI1
MYL7
vCM Suptype
Com.
FZD1
HEY2
Trab.
NPPA
NPPB
Mito.
PARGC1A
CREB1
MEF2C
Expression
2 1 0 -1 -2
Legend
CM
CM + EPC->DC
CM(+SB)
CM + EPC(+SB)
B.
DEG Prolif. vCM
MYH7
ID3
CASQ2
ENO3
COL18A1
CCDC34
MYL2
HOPX
COL5A2
TNNI3
VCAN
EIF1B
SORBS2
BEX1
HSPB7
PRRX1
TNNT2
MYL4
MYL6
ACTC1
MYL3
DHRS3
ACTA2
ZFP36L1
FBN2
CYP51A1
PPP1R1A
MYOZ2
LRATD2
ACTN2
NES
PYGB
MYOM1
CXCL12
TMEM176B
TMEM176A
CSRP3
IGFBP6
SOCS3
PDLIM3
MXRA7
SOX3
DENND5A
MGST3
SYNPO2L
Expression
2 1 0 -1 -2
C.
Upregulated
MORPHOGENESIS OF AN EPITHELIUM
ACTIN FILAMENT ORGANIZATION
MUSCLE TISSUE DEVELOPMENT
RESPONSE TO PEPTIDE HORMONE
FATTY ACID METABOLIC PROCESS
MUSCLE CONTRACTION
RESPONSE TO INSULIN
REGULATION OF CANONICAL WNT SIGNALING PATHWAY
RESPONSE TO TRANSFORMING GROWTH FACTOR BETA
HEART PROCESS
CARDIAC MUSCLE TISSUE DEVELOPMENT
ACTOMYOSIN STRUCTURE ORGANIZATION
REGULATION OF HEART CONTRACTION
CARDIAC CHAMBER DEVELOPMENT
REGULATION OF MUSCLE CONTRACTION
RESPONSE TO CALCIUM ION
CARDIAC MUSCLE CONTRACTION
ACTIN FILAMENT BASED MOVEMENT
CARDIAC VENTRICLE DEVELOPMENT
CARDIAC CHAMBER MORPHOGENESIS
ACTIN MEDIATED CELL CONTRACTION
CARDIAC CONDUCTION
HOMOTYPIC CELL CELL ADHESION
MUSCLE HYPERTROPHY
VASCULOGENESIS
CARDIAC MUSCLE CELL ACTION POTENTIAL
REGULATION OF ATP DEPENDENT ACTIVITY
CARDIAC VENTRICLE MORPHOGENESIS
STEROL BIOSYNTHETIC PROCESS
CELLULAR RESPONSE TO RETINOIC ACID
REGULATION OF SMOOTH MUSCLE CONTRACTION
NEGATIVE REGULATION OF MAP KINASE ACTIVITY
CARDIAC MUSCLE TISSUE MORPHOGENESIS
VENTRICULAR CARDIAC MUSCLE TISSUE DEVELOPMENT
HEART VALVE MORPHOGENESIS
VENTRICULAR CARDIAC MUSCLE TISSUE MORPHOGENESIS
SARCOMERE ORGANIZATION
CARDIAC CONDUCTION SYSTEM DEVELOPMENT
CGMP MEDIATED SIGNALING
REGULATION OF CARDIAC MUSCLE CELL ACTION POTENTIAL
MUSCLE HYPERTROPHY IN RESPONSE TO STRESS
PROTEIN LOCALIZATION TO CELL CELL JUNCTION
CARDIAC MYOFIBRIL ASSEMBLY
NEGATIVE REGULATION OF JUN KINASE ACTIVITY
CM
CM + EPC->DC
CM + EPC(+SB)
CM(+SB)
Gene Count
4
8
12
16
qvalue
0.075
0.050
0.025
D.
Downregulated
ACTIN FILAMENT ORGANIZATION
MUSCLE TISSUE DEVELOPMENT
RESPONSE TO PEPTIDE HORMONE
CELL SUBSTRATE ADHESION
MUSCLE CONTRACTION
REGULATION OF CANONICAL WNT SIGNALING PATHWAY
RESPONSE TO TRANSFORMING GROWTH FACTOR BETA
HEART PROCESS
CELL MATRIX ADHESION
CARDIAC MUSCLE TISSUE DEVELOPMENT
ACTOMYOSIN STRUCTURE ORGANIZATION
REGULATION OF HEART CONTRACTION
CARDIAC CHAMBER DEVELOPMENT
REGULATION OF MUSCLE CONTRACTION
CARDIAC MUSCLE CONTRACTION
ACTIN FILAMENT BASED MOVEMENT
CARDIAC VENTRICLE DEVELOPMENT
CARDIAC CHAMBER MORPHOGENESIS
ACTIN MEDIATED CELL CONTRACTION
CARDIAC CONDUCTION
HOMOTYPIC CELL CELL ADHESION
MUSCLE HYPERTROPHY
VASCULOGENESIS
CARDIAC MUSCLE CELL ACTION POTENTIAL
REGULATION OF ATP DEPENDENT ACTIVITY
CARDIAC VENTRICLE MORPHOGENESIS
STEROL BIOSYNTHETIC PROCESS
NEGATIVE REGULATION OF MAP KINASE ACTIVITY
CARDIAC MUSCLE TISSUE MORPHOGENESIS
VENTRICULAR CARDIAC MUSCLE TISSUE DEVELOPMENT
VENTRICULAR CARDIAC MUSCLE TISSUE MORPHOGENESIS
EXTRACELLULAR MATRIX ASSEMBLY
SARCOMERE ORGANIZATION
CARDIAC CONDUCTION SYSTEM DEVELOPMENT
CELL COMMUNICATION BY ELECTRICAL COUPLING
BASEMENT MEMBRANE ORGANIZATION
CGMP MEDIATED SIGNALING
CELL COMMUNICATION BY ELECTRICAL COUPLING INVOLVED IN CARDIAC CONDUCTION
MUSCLE HYPERTROPHY IN RESPONSE TO STRESS
CARDIAC MYOFIBRIL ASSEMBLY
CELL CELL JUNCTION MAINTENANCE
NEGATIVE REGULATION OF JUN KINASE ACTIVITY
CM
CM + EPC->DC
CM + EPC(+SB)
CM(+SB)
qvalue
0.075
0.050
0.025
Gene Count
10
20
E.
Response to Calcium Ion
Cardiac Conduction
Canonical WNT Signaling Pathway
Cardiac Ventricle Development
CM + EPC(+SB)
CM + EPC->DC
CM
CM(+SB)
PRICKLE1 LGR4 DAPK3 DACT1 BAMBI IGFBP6 EGR1 EDA FZD1 NKX2.5 WWTR1 MDK WNT5A MCC
CORIN ATP1A2 HRC GJC1 ATP2B4 GJA1 CASQ2 SLC8A1 MEF2A RYR2 GJA5 NKX2.5 DSP ATP2A2 CACNB2 PDE4D
SLC25A23 CASQ2 JUN JUND TXNIP FOS JUNB RYR2 ACTG1 TNNT2 TRPC3 WNT5A CPNE5
NPNT DAPK3 ST6GAL1 CSPG5 TNNI3 MYL2 MYH7 SMAD6 CITED2 GJA5 MYL3 FZD1 RYR2 NKX2.5 DSP TNNT2 WNT5A ROBO1 HEY2
column min
column max

Figure S5. Pooled proliferative vCM by condition DEG and ORA, related to Figure 5.

A) A heat map of cardiac genes of interest for all the proliferative vCM broken down by condition. B) Heat map of the top 15 differentially expressed genes between the proliferative vCM broken down by condition. Dot plots showing some of the ORA biological processes (BP) pathways that were significantly C) upregulated or D) downregulated in the proliferative vCM by conditions. E) Heat map of some of the differentially expressed genes contributing to enrichment in four GO BP pathways 1) Cardiac Ventricle Development, 2) Regulation of Canonical WNT Signaling Pathway, 3) Cardiac Conduction, and 4) Response to Calcium Ions.

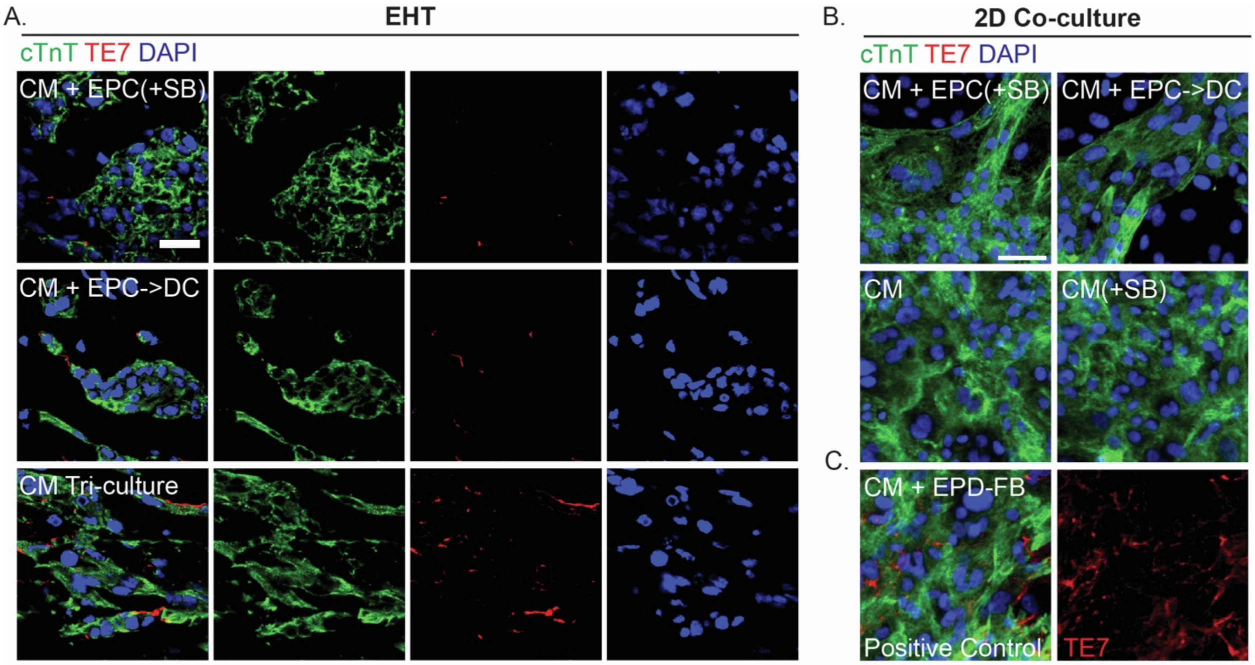

Figure S6. Engineered heart tissues and 2D co-culture fibroblasts content.

A) Staining of cTnT (green – cardiomyocyte), TE7 (red – fibroblasts), and DAPI (blue – nucleus) in EHT sections from the CM + EPC(+SB) (top), CM + EPC->DC (middle) and CM Tri-culture (bottom) conditions. Scale 20 µm. B) Staining of cTnT (green – CM), TE7 (red – fibroblasts), and DAPI (blue – nucleus) in all four 2D co-culture conditions and a C) positive control where fully differentiated EPD-FBs were co-cultured with CM in 2D. Scale 50 µm.

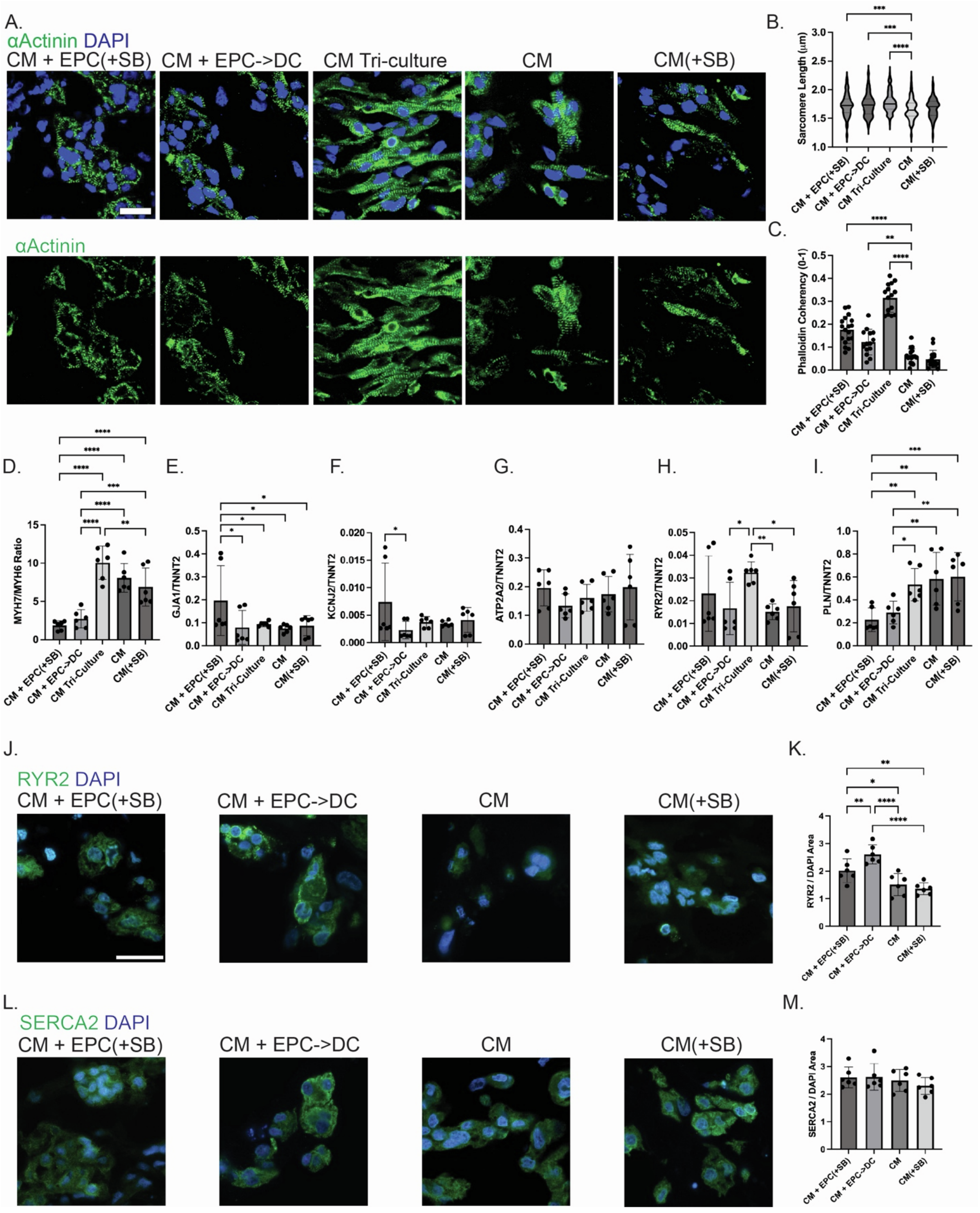
A.
αActinin DAPI
CM + EPC(+SB)
CM + EPC->DC
CM Tri-culture
CM
CM(+SB)
αActinin
B.
Sarcomere Length (μm)
C.
Phalloidin Coherency (0-1)
D.
MYH7/MYH6 Ratio
E.
GJA1/TNNT2
F.
KCNJ2/TNNT2
G.
ATP2A2/TNNT2
H.
RYR2/TNNT2
I.
PLN/TNNT2
J.
RYR2 DAPI
CM + EPC(+SB)
CM + EPC->DC
CM
CM(+SB)
K.
RYR2 / DAPI Area
L.
SERCA2 DAPI
CM + EPC(+SB)
CM + EPC->DC
CM
CM(+SB)
M.
SERCA2 / DAPI Area

Figure S7. EHT Phenotypic maturation, related to Figure 7.

A) Representative images of αActinin (green) and DAPI (blue) in EHT cryosections. Scale 50 μm. B) Quantified sarcomere length and C) Cellular alignment in the EHTs as shown by the average coherency of phalloidin from EHT transverse cross-sections where 0 is randomly aligned and 1 is perfectly aligned. Quantitative rt-PCR of the D) *MYH6/MYH7* ratio and E) *GJA1*, F) KCNJ2, G) *ATP2A2* H) *RYR2,* and I) *PLN* normalized to the housekeeping gene *EDF1* and the cardiac gene *TNNT2*. J) Representative images of RYR2 (green) and DAPI (blue) in EHT cryosections with K) quantification of RYR2 area normalized to DAPI area. L) Representative images of SERCA2 (green) and DAPI (blue) in EHT cryosections with M) quantification of RYR2 area normalized to DAPI area. For (B) violin plot center dashed line represents the median and outer dashed lines represent the upper and lower quartiles of the distribution and CM + EPC(+SB) (n = 162), CM + EPC->DC (n = 174), CM Tri-culture (n = 191), CM (n = 145), CM(+SB) (n = 125) sarcomeres were measured across three sections per EHT and 6 EHTs per condition across three independent experiments. For (C) the phalloidin coherency was determined for CM + EPC(+SB) (n = 18), CM + EPC->DC (n = 14), CM Tri-culture (n = 15), CM (n = 17), CM(+SB) (n = 15) EHT cross-sections across 6 EHTs per conditions from three independent experiments. For (D-I) each dot represents one technical replicate from n = 3 EHTs per condition across three independent experiments. For (K) and (M), each dot represents a field view across two EHTs for three replicates. See also **Table S3**.

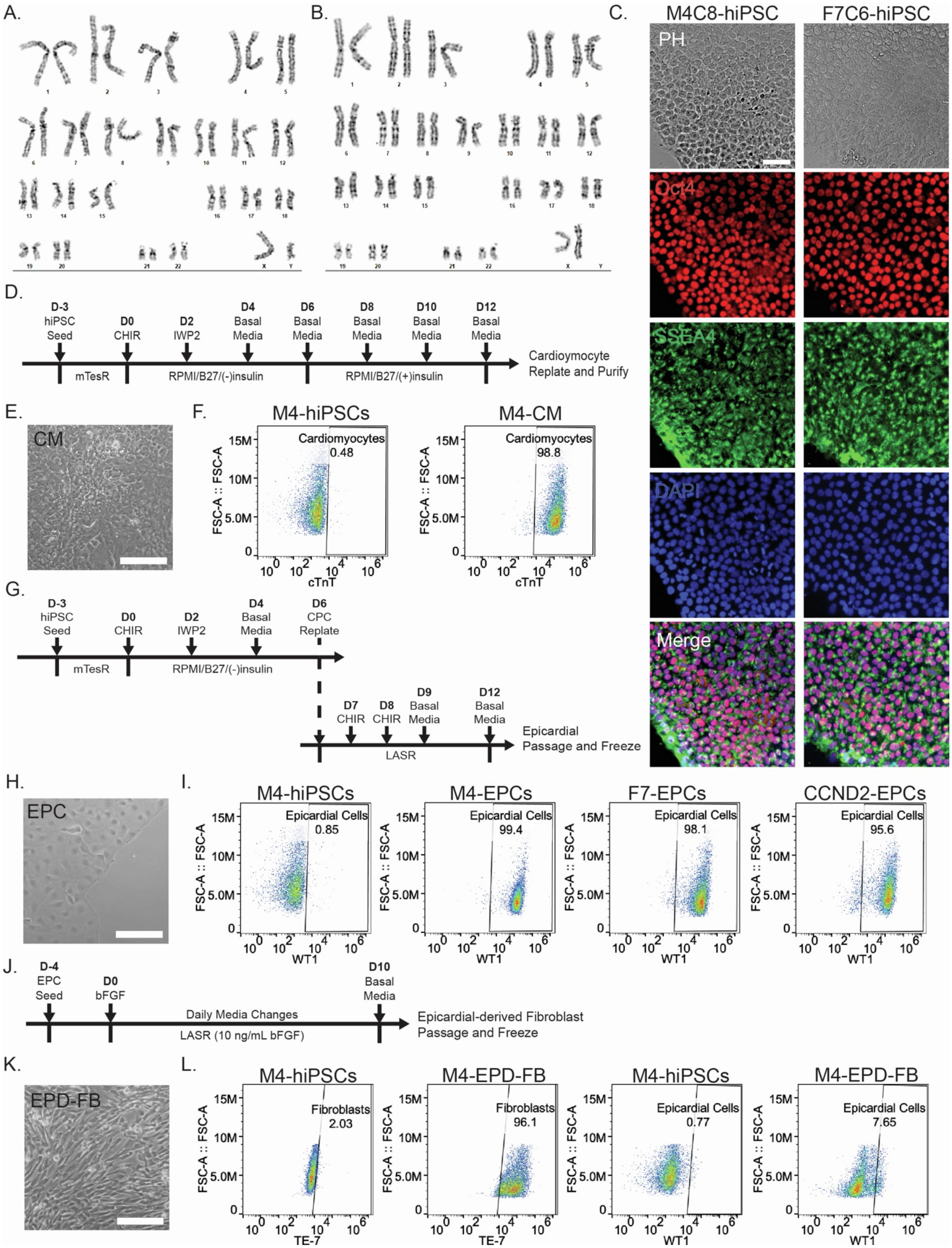
A.
B.
C.
M4C8-hiPSC
F7C6-hiPSC
PH
Oct4
SSEA4
DAPI
Merge
D.
D-3 hiPSC Seed
D0 CHIR
D2 IWP2
D4 Basal Media
D6 Basal Media
D8 Basal Media
D10 Basal Media
D12 Basal Media
Cardioymocyte Replate and Purify
mTesR
RPMI/B27/(-)insulin
RPMI/B27/(+)insulin
E.
CM
F.
M4-hiPSCs
M4-CM
Cardiomyocytes 0.48
Cardiomyocytes 98.8
FSC-A :: FSC-A
cTnT
G.
D-3 hiPSC Seed
D0 CHIR
D2 IWP2
D4 Basal Media
D6 CPC Replate
mTesR
RPMI/B27/(-)insulin
D7 CHIR
D8 CHIR
D9 Basal Media
D12 Basal Media
Epicardial Passage and Freeze
LASR
H.
EPC
I.
M4-hiPSCs
M4-EPCs
F7-EPCs
CCND2-EPCs
Epicardial Cells 0.85
Epicardial Cells 99.4
Epicardial Cells 98.1
Epicardial Cells 95.6
WT1
J.
D-4 EPC Seed
D0 bFGF
D10 Basal Media
Daily Media Changes
LASR (10 ng/mL bFGF)
Epicardial-derived Fibroblast Passage and Freeze
K.
EPD-FB
L.
M4-hiPSCs
M4-EPD-FB
Fibroblasts 2.03
Fibroblasts 96.1
Epicardial Cells 0.77
Epicardial Cells 7.65
TE-7
WT1

Figure S8. M4 and F7-hiPSC Pluripotency and Karyotype and the differentiation and purity of hiPSC-EPC, EPD-FB, and CM.

The full karyotype of the A) M4-hiPSC lines and the B) F7-hiPSC lines. C) Representative images of the M4-hiPSC line (left) and the F7-hiPSC line (right) stained for the pluripotency markers Oct4 (red) and SSEA4 (green) and a nuclear stain DAPI. D) Schematic of the hiPSC-CM small molecule differentiation protocol. E) Brightfield image of D24 CM post-replating and lactate purification as well as F) flow cytometry for CM before engineered heart tissue seeding using M4-hiSPCs as a negative control for cTnT gating. G) Schematic of the hiPSC-EPC small molecule differentiation protocol. H) A brightfield image of the hiPSC-EPCs and I) flow cytometry for the epicardial marker WT1 for M4-hiPSCs as a negative control and P2-4 EPCs from the M4, F7, and CCND2 hiPSC-lines used for this study. J) A schematic of the hiPSC-EPD-FB differentiation protocol and K) a brightfield image of the EPD-FBs. L) Flow cytometry for the fibroblast marker TE7 to assess the purity of the EPF-FB differentiation using M4-hiPSCs as a negative control for gating. Scale 50 μm.

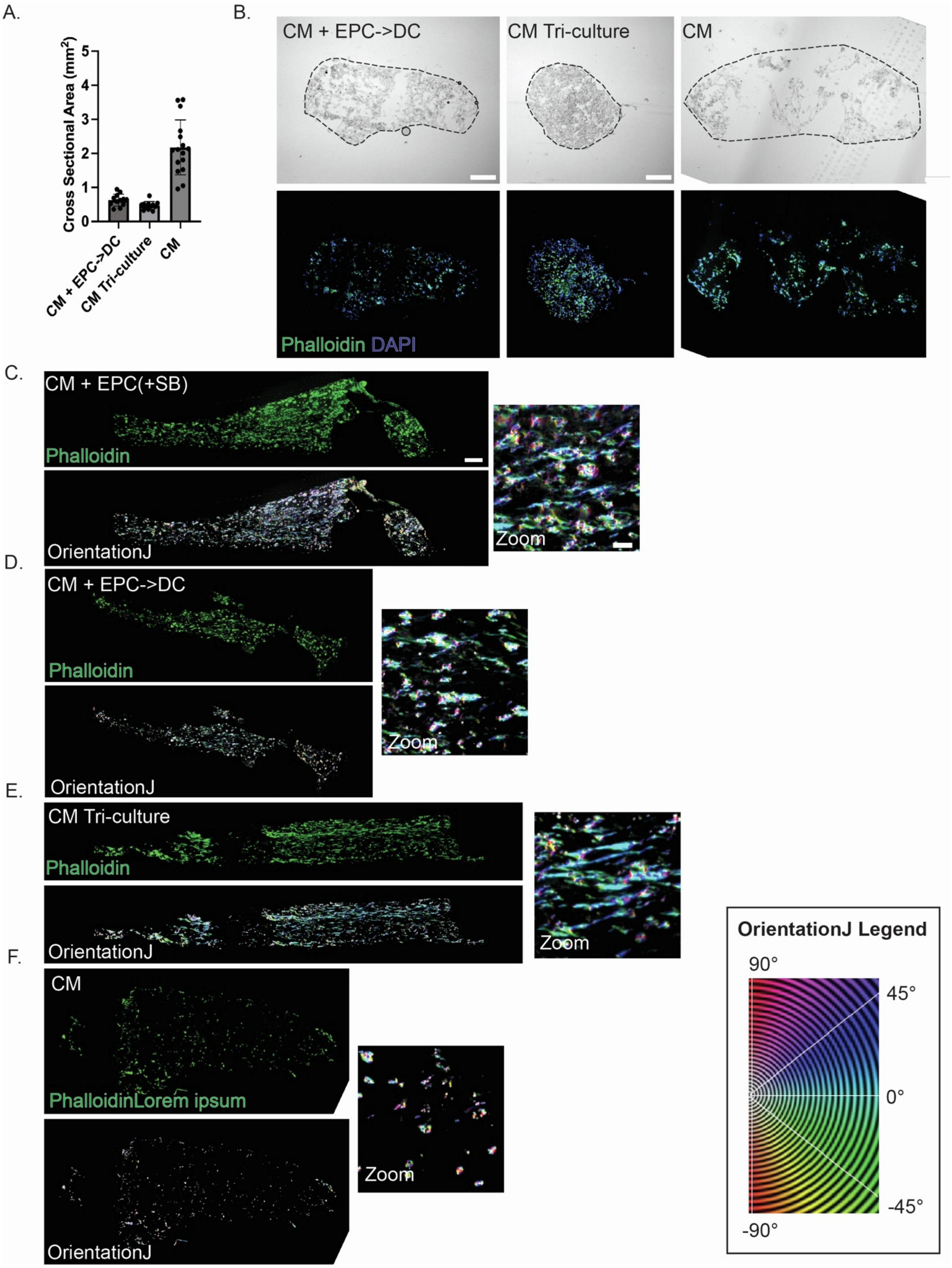

A.
Cross Sectional Area (mm²)
5
4
3
2
1
0
CM + EPC->DC
CM Tri-culture
CM
B.
CM + EPC->DC
CM Tri-culture
CM
Phalloidin DAPI
C.
CM + EPC(+SB)
Phalloidin
OrientationJ
Zoom
D.
CM + EPC->DC
Phalloidin
OrientationJ
Zoom
E.
CM Tri-culture
Phalloidin
OrientationJ
Zoom
F.
CM
PhalloidinLorem ipsum
OrientationJ
Zoom
OrientationJ Legend
90°
45°
0°
-45°
-90°

Figure S9. EHT cross-sectional area measurements for twitch force calculation and EHT phalloidin staining and OrientationJ coherency measurements, related to Figure S7.
A) A bar graph of the average cross-sectional area for each of the force-generating EHT conditions. B) A bright field image with the cross-sectional area of the EHTs highlighted, followed by phalloidin (green) and DAPI (blue) images for better cellular visualization within the EHT cross-sections. Scale 250 µm. Representative transverse cross-sections of EHTs showing F-actin (green - phalloidin - top) and the orientation color map (bottom) showing the average angle of each F-actin fiber. For the C) CM + EPC(+SB), D) CM + EPC->DC, E) CM Tri-culture and F) CM EHTs. Scale 250 µm for whole sections and 20 µm for zoomed-in fields of view.

Supplemental Tables

Table S1 Single-cell RNA sequencing vCM integration DEG and BP pathways analysis, related to Figure 5.

Comparison	Condition	# of DEG	# BP Pathways UP	# BP Pathways DOWN
**vCM by Cluster**	Cluster 0	85	31	2
**vCM by Cluster**	Cluster 3	323	234	147
**vCM by Cluster**	Cluster 5	246	47	81
**vCM by Cluster**	Cluster 9	153	84	4
**vCM by Cluster**	Cluster 10	246	109	131
**vCM by Condition**	CM + EPC(+SB)	332	193	158
**vCM by Condition**	CM + EPC->DC	189	65	9
**vCM by Condition**	CM	106	29	65
**vCM by Condition**	CM(+SB)	139	34	49
**Proliferative vCM by Condition**	CM + EPC(+SB)	295	154	96
**Proliferative vCM by Condition**	CM + EPC->DC	148	15	37
**Proliferative vCM by Condition**	CM	97	28	58
**Proliferative vCM by Condition**	CM(+SB)	119	46	37

Table S2. Antibody dilution and catalog number.

Target	Dilution	Isotype	Conjugate	Assay	Supplier	Catalog #
αActinin	1:200	Mouse IgG1	None	IHC	Abcam	ab9465
cTnT	1:400	Mouse IgG1	None	IHC/FC	Thermo Fisher	MS-295-P1
cTnT	1:200	Rabbit IgG	None	IHC	Abcam	ab209813
Ki67	1:100	Rabbit IgG	None	FC	Thermo Fisher	MA5-14520
Ki67	1:100	Rabbit IgG	None	IHC	Cell Signaling	9129S
MLC2v	1:500	Rabbit IgG	None	IHC	Abcam	ab79935
MLC2v	1:1000	Rabbit IgG	None	FC	Abcam	ab79935
Mouse IgG	1:400	Goat	AF-488	IHC/FC	Thermo Fisher	A11001
Mouse IgG	1:400	Goat	AF-647	FC	Thermo Fisher	A21235
Nkx2.5	1:200	Mouse IgG1	None	IHC	Santa Cruz Biot.	sc-8697
Oct4	1:250	Rabbit IgG	None	IHC	Thermo Fisher	A24869
Rabbit IgG	1:400	Goat	AF-647	IHC/FC	Thermo Fisher	A21245
RYR2	1:500	Mouse	None	IHS	Novus Biologicals	NB300-543
SERCA2 (ATP2A2)	1:100	Mouse	None	IHC	Invitrogen	MA3919
SSEA4	1:250	Mouse IgG3	None	IHC	Thermo Fisher	A24870
TE7	1:100	Mouse IgG1	None	IHC	EMD Millipore	CBL271
WT1	1:200	Rabbit IgG	None	IHC	Abcam	ab88901

Table S3. q-RT-PCR Primers, related to Figure S7.

Gene	Forward Primer	T_m	Reverse Primer	T_m	Product Size
ATP2A2	CCGGACTTTGAAGGCGTGGATTG	62	CCTCAGCAAGGACTGGTTTTCGG	63	132
EDF1	ATCTTAGCGGCACAGAGACGAG	60	TGTCATGGTGCAGCTCCTCTGT	63	133
GJA1	GGAGATGAGCAGTCTGCCTTTCG	62	ATGAGCCAGGTACAAGAGTGTGG	60	150
KCNJ2	AACAGTGCAGGAGCCGCTTTGT	64	AGGACGAAAGCCAGGCAGAAGA	62	160
MYH6	GGAAGACAAGGTCAACAGCCTGT	61	TCCAGTTTCCGCTTTGCTCGC	63	129
MYH7	GGAGTTCACACGCCTCAAAGAGG	61	TCCTCAGCATCTGCCAGGTTGT	63	147
RYR2	TCTTGAGGTTGGCTTTCTGCCAG	61	CTGTGCCAGCAAAGAGAGGAGCA	62	155
TNNT2	AGAAGAGGAAGCAAAGGAGGCTG	61	AAGTCCACTCTCTCTCCATCGG	59	117

Supplemental Methods

Human induced pluripotent stem cell lines

Three hiPSC-lines were used for these studies. All of the lines were derived from left ventricular cardiac fibroblasts—two lines from female donors, CCND2-hiPSC and F7C6-hiPSC, and one line from a male donor M4C8-hiPSC. The CCND2-hiPSCs were obtained from Jianyi Zhang's lab at the University of Alabama Birmingham and were previously characterized(Zhu et al., 2018). The other lines were reprogrammed in-house using Cyto-Tune 2.0 Sendai Virus reagents following the supplier's manual (Thermo A16517). Sendai virus was confirmed to be depleted via qPCR for two passages. A full karyotype was performed on the lines after Sendai virus depletion was confirmed and pluripotency was seen to be high and the karyotype showed no abnormalities (**Figure S7A-C**). Master banks were created and the STEMCELLS were used within 15 passages of the bank being created.

Human induced pluripotent stem cell maintenance

The hiPSCs were cryopreserved in mFresR^{TM} (STEMCELL 05855). The hiPSCs were maintained in a 6 well plate coated with 8.68 μg/cm^2, or 0.5 mg per well, Cultrex-reduced growth factor (RGF) (R&D Systems 3433-005-01) in 2 mL of mTesR^{TM}1 (STEMCELL 85850) and passaged using ReLesSR^{TM} (STEMCELL 05872) upon reaching 70-80% confluency as follows. Exhausted media was aspirated, and cells were washed with 1 mL of DPBS without calcium or magnesium. Then, 1 mL of room temperature ReLesSR^{TM} was added to each well and incubated for 45 seconds. The ReLesSR^{TM} was aspirated, and the plate was put into the incubator at 37°C for 7 minutes. Warm mTesR^{TM}1 was added to the well and the edges of the plate were gently tapped to release and break the hiPSC colonies from the bottom of the plates. The cell suspension was diluted and transferred into a new 6 well-plate. All cultures were visually inspected for the presence of bacterial and fungal contaminants.

CM differentiation and purification

The hiPSCs were maintained until reaching 70-80% confluency and singularized using Accutase® solution (Millipore Sigma A6964) as follows. Exhausted media was aspirated, and the cells were washed with 1mL of DPBS without calcium or magnesium, and 1mL of room temperature Accutase® solution was added to each well and incubated at 37°C for 8 minutes. The hiPSCs were singularized by pipetting them up and down with a P1000 micropipette and quench using ½ mL of mTesR™1. The cell suspension was transferred to a conical tube, mixed, and counted on a hemocytometer using a 1:1 dilution of 0.4% trypan blue. The hiPSCs were seeded onto 8.68 μg/cm^2 Cultrex-RGF at a density of ~132,000 cells/cm^2, or 0.5x10^6 cells per 12 well, into 1mL of mTesR™1 with 5μM Rock Inhibitor (VWR 103538-728). The exhausted media was replaced with 2mL of fresh mTesR™1 until the cells reached 97-100% confluency (2-4 days). All media changes unless otherwise specified were a volume of 2mL. Upon reaching confluency, defined as day 0 of the differentiation, the media was replaced with RPMI/B27(-ins) (Fisher A1895601) containing 6-8 μM CHIR99021 (Sigma SML1046). The exact CHIR99021 concentration for mesoderm specification depends on the hiPSC-line. After 48 hours, on day 2 of differentiation, the media was replaced with RPMI/B27(-ins) containing 7.5 μM of IWP2 (Tocris 686770-61-6). After another 48 hours, on day 4 of differentiation, the media was replaced with fresh RPMI/B27(-ins). On day 6, and every two days until day 13 of differentiation, the media was replaced with fresh RPMI/B27(+ins) (Thermo 17504001). The timeline is depicted visually in the supplemental materials (**Figure S7D**). To aid in lactate purification, the CM were replated at a 1:2 ratio onto 8.68 μg/cm^2 Cultrex-RGF coated 12 well-plates. Briefly, each well was washed with 1mL of DPBS without calcium or magnesium then 1mL 37°C 0.25% trypsin-EDTA solution was added to each well and incubated at 37°C for 20 minutes. The CM were singularized by pipetting with a P1000 micro pipettor and then quenched with 2mL of RPMI/B27(+ins) with 20% FBS. The cell suspension was transferred to a conical tube and centrifuged at 200g for 5 minutes. The supernatant was aspirated from the cell pellet and the cells were resuspended in RPMI/B27(+ins) containing 10 μM Rock Inhibitor. On day 14, the replated cells are given 1mL of RPMI/B27(+ins). The CM were purified using lactate purification media (DMEM no glucose with 4 mM lactate) (Thermo 11966025, Sigma L7022). On day 15 the media was replaced with 1 mL lactate purification medium. On day 17 the cells were washed with 1mL of DPBS without calcium or magnesium, and the media was replaced

with 1 mL lactate medium. On day 19 the cells were washed with DPBS without calcium or magnesium again and given fresh RPMI/B27(+ins) every three days until replated for 2D co-culture or engineered heart tissue seeding. Differentiation quality controls show cTnT > 95% (**Figure S7E-F**).

Epicardial differentiation

The epicardial differentiation protocol was adapted from a previous publication and visually depicted in the supplement (**Figure S7G**)[36]. The CM differentiation as detailed above is followed until day 6. On day 6, the cardiac progenitor population is replated onto a gelatin-coated 6-well plate at a density of 30,000 cells/cm^2. Briefly, each well was washed with 0.5 mL of DPBS without calcium or magnesium then incubated in 0.5 mL of Accutase® for 8 minutes at 37°C. The cardiac progenitors were singularized using a P1000 micro pipettor and then quenched with 0.5 mL LASR media (DMEM/F12 Advanced, 1.25% GlutaMAX, 60 μg/mL Ascorbic Acid). The cell suspension was transferred into a conical tube and counted using a hemocytometer with a 1:1 dilution of 0.4% trypan blue. The cells were plated onto a gelatin-coated 6-well plate in LASR media with a 5 μM Rock Inhibitor. All media changes were a volume of 2 mL for this differentiation process. On days 7 and 8, the medium is replaced with LASR containing 2-3 μM CHIR99021. The exact CHIR99021 concentration for epicardial specification depends on the hiPSC-line. Starting on day 9 the cells are given fresh LASR media every 2-days until day 12. On day 12 the epicardial cells (EPCs) are passaged or cryopreserved at 1 million cells per vial in epicardial freezing medium (LASR media with 30% FBS, 10% DMSO, 5 μM Rock Inhibitor, 2 μM SB431542). Epicardial cells were used between passages 2 and 5 in these experiments and were all cryopreserved before use. Briefly, the epicardial cells can be thawed onto a gelatin-coated 6-well plate into LASR media with 1% FBS, 5 μM Rock Inhibitor, and 2 μM SB431542. The media was changed with LASR media containing 2 μM SB431542 daily until the cells reached 80-90% confluency. At this point, the cells were passaged or used for a co-culture experiment. The epicardial cells were passaged at a 1:3-1:6 ratio onto a gelatin-coated plate using Versene (Thermo 15040066). Each well was washed with 1 mL of DPBS without calcium or magnesium and then incubated in 1 mL of Versene for 8 minutes at 37°C. The Versene was aspirated and 1 mL of fresh LASR media with 1% FBS and 2 μM SB431542 was added to the

cell layer. The cells are broken into clumps using a P1000 micro pipettor, transferred into a conical tube, diluted, and replated into a new gelatin-coated 6-well plate. The differentiated epicardial cells had a distinct epithelial cobblestone morphology (**Figure S7H**) and were determined to be ≥ 95% pure via flow cytometry for WT1 (Wilms tumor 1) (**Figure S7I**).

Epicardial-derived fibroblast differentiation

The epicardial-derived fibroblast differentiation was adapted from a previous publication and can be visualized in the supplemental materials (**Figure S7J**)[38]. At day 12 of epicardial differentiation the epicardial cells were passaged twice, as detailed above without cryopreservation, and then grown until they reached 100% confluency in LASR media with 2 μM SB431542. At this point, the medium was replaced with LASR containing 10 ng/mL bFGF (R&D Systems 233-FB-010) daily for 10 days. At day 10, the cells were replated at a 1:6 ratio onto tissue culture plastic into fully supplemented FibroGRO (Millipore Sigma SCMF002). To passage, the epicardial-derived fibroblasts (EPD-FBs) were washed, with 1 mL of DPBS without calcium or magnesium then incubated in 1 mL of Accutase® for 20 minutes at 37°C. The cells were broken up using a P1000 micro pipettor and plated into FibroGRO on tissue culture plastic at a 1:6-1:18 ratio. The FibroGRO medium was replaced every 2 days with fresh media until the cells reached 70-90% confluency. The EPD-FBs were used in engineered heart tissue experiments between passage 3 and passage 7. The fibroblasts had an elongated stomal cell morphology, were ≥ 95% pure via flow cytometry for TE7, and had less than 10% remaining WT1-positive cells (**Figure S7K-L**).

Direct co-culture of CM with epicardial cells

CM and EPC co-cultures were seeded in a layered fashion to allow for maximum and consistent CM attachment. First, the plates (12 and 48 well-plates) were coated overnight at 37°C with 17.36 μg/cm^2 of Cultrex-RGF. On day 26 of CM differentiation the CM were washed with 0.5 mL of DPBS without calcium or magnesium and 0.5 mL 37°C 0.25% trypsin-EDTA solution was added to each well and incubated at 37°C for 20 minutes. The CM was singularized by pipetting with a P1000 micro pipettor and then quenched with 1mL of RPMI/B27(+ins) with

20% FBS and 5 μM Rock Inhibitor. The cell suspension was transferred to a conical tube and counted with a hemocytometer with a 1:1 dilution of 0.4% trypan blue. The cells were centrifuged at 200g for 5 minutes and plated at a density of 75,000 cells/cm^2 in LASR media containing 10 μM Rock Inhibitor. On day 27, after the CM had fully attached, the epicardial cells were seeded on top of the CM at a density of 25,000 cells/cm^2 in LASR containing 5 μM Rock Inhibitor (CM + EPC->DC) and 2 μM SB431542 (For the CM + EPC(+SB) condition only). On day 27 the CM-only conditions were given LASR containing 5μM Rock Inhibitor (CM) and 2μM SB431542 (For the CM(+SB) condition only). The media was changed to fresh LASR with or without 2μM SB431542 on day 28 and every 2 days until day 35 of CM differentiation (8 days of co-culture total). To label newly synthesized DNA co-cultures controls that were fated for flow cytometry proliferation assessment were given LASR containing 10 μM 5-ethynyl 2'-deoxyuridine (EdU) for 24 hours before singularization and fixation. On day 35, the CM-only controls and co-cultures were assessed and processed in various ways as detailed below. The 2D co-culture timeline is depicted in **Figure 1A**.

Engineered heart tissue seeding

The EHT negative mold was printed on a Stratasys J750 PolyJet 3D Printer using digital acrylonitrile butadiene styrene (ABS). The ABS negative mold was thoroughly cleaned, oxygen plasma treated for 10 minutes (PDC-32G, Harrick Plasma), and treated with silane vapors for 24-hours in a desiccator vacuum. Sylgard-184 polydimethylsiloxane (PDMS) precursor was mixed at a 1:10 mass ratio with a PDMS curing agent and cast onto the ABS negative mold. The curing PDMS was degassed for 1 hour at room temperature and cured overnight at 50°C. The resultant PDMS mold contains 12 rectangular EHT culture wells and each EHT well contains two posts. The PDMS mold was further cured at 50°C for 1 week. The mold was then cut into six 2-mold squares that were sonicated in 70% ethanol for 30 minutes and autoclaved at 121°C for 25 minutes. The sterile two EHT well PDMS squares were transferred into a sterile petri dish and oxygen plasma was treated for 2 minutes. The PDMS EHT molds were then transferred to a 12-well plate and treated with 0.5 w/v% Pluronic F-127 (Sigma P2443) for 30 minutes at room temperature. At day 24 of CM differentiation the purified hiPSC-CM were dissociated in 0.25% Trypsin-EDTA and combined with EPCs or EPCs and EPD-FBs (CM Tri-culture) that were

singularized in Accutase®. The purity of the CM used in the EHTs were assessed to be ≥95% pure via flow cytometry for cardiac troponin T (cTnT) (**Figure S7F**). The hiPSC-CM and EPCs or EPCs and EPD-FBs were combined at a 3:1 ratio at a density of $10x10^6$ cells/mL in a fibrin gel mixture containing 20mg/mL fibrinogen from human plasma (Sigma F3879), LASR with 25 µM Rock Inhibitor and 100U/mL thrombin from human plasma (Sigma 605190) at a 6:3:1 ratio. Each PDMS EHT culture well was made with 100 µL of fibrin gel cell suspension which instantly formed a gel at room temperature upon the addition of thrombin. For the CM-only control conditions, $7.5x10^6$ CM/mL of fibrin gel mixture was used so that all conditions contained the same number of CM. Two EHTs of the same condition were cultured per well of a 12-well plate. The EHTs were cultured in LASR with 10 µM Rock Inhibitor, 20 µg/mL aprotinin (Sigma 10981532001), and (+/-) 2 µM SB431542 for 24 hours after seeding. The EHTs were then given fresh LASR media with 20 µg/mL aprotinin (+/-) 2µM SB431542 every two days until assessment at 29-31 days after EHT seeding. The EHTs fated for immunohistochemical (IHC) analysis were treated with 10 µM EdU for 24 hours before fixation and processing as detailed below.

Calcium transients of direct co-cultures, transwell co-cultures and EHTs

On day 35, the 2D co-cultures and controls that were plated into 12 well-plates were incubated with 5 µM Rhod-2AM calcium-sensitive dye in LASR medium for 30 minutes at 37°C. For the EHTs calcium was assessed 29-31 days after EHT seeding. The EHTs were kept in the PDMS mold and incubated with 5µM Rhod-2AM calcium-sensitive dye in LASR medium for 30 minutes at 37°C. The dye was aspirated and replaced with Tyrode's Salt Solutions with 1 g/L sodium bicarbonate (TSS) (Sigma Aldrich T2145). The 2D cells or EHTs were equilibrated in TSS for 30 minutes at 37°C and then imaged on the TxRed channel with a 30-millisecond exposure and gain of 1.0 at 10X magnification on a fluorescent microscope (Leica Microsystems). Three 20-second videos per well or EHT were taken, with n=3 wells per condition or n=3-4 EHTs per condition for each replicate. Videos were taken at a frame rate of 7 frames/s. The co-cultures and EHTs were paced using a platinum electrode at 1 Hz with a voltage of 7 V and pulse duration of 0.02- milliseconds. The calcium transient data acquired was

processed in ImageJ by importing the images and converting them into image stacks. Three regions of interest within each video were selected and the Z-axis profile of fluorescent intensity versus time was exported as a text file. The text files were analyzed using an in-house MATLAB code to determine the inter-spike-interval (s) of the calcium peaks, the time to peak (s), the maximum amplitude (F/F_0) of the calcium peak as well as the average upstroke and downstroke velocities ($F/F_0/s$). Any videos acquired in which the inter-spike-interval was less than 0.95 or greater than 1.05 were excluded due to insufficient pacing.

Patch clamp of CM

Patch clamp was done for the CM + EPC(+SB) and the CM(+SB) control only. The direct 2D co-cultures were replated at a density of ~10,000 cells/cm^2 onto 35 mm TC-treated petri dishes coated with 17.36 µg/cm^2 Cultrex-RGF. After 24 hours patch clamp measurements of the replated CM were taken. The patch clamp and AP recording method is described in a previous publication(Xie et al., 2022). In brief, an Axopatch-200B amplifier (Molecular Devices, Foster City, USA) was used to record APs by a ruptured whole-cell current-clamp technique. For AP recordings, pipettes were filled with (in mmol/L) 120 potassium gluconate, 20 KCl, 5 NaCl, 5 HEPES, 0.02 EGTA, 0.05 $CaCl_2$, and 5 MgATP (pH 7.2, $[Ca2^{2+}]_i \approx 100$ nmol/L). The extracellular bathing solution contained (in mmol/L) 140 NaCl, 5.4 KCl, 1 $MgCl_2$, 10 HEPES, 1.8 $CaCl_2$, and 5.5 glucose (pH 7.4). Pipette resistances were ~3 MΩ. The recordings were low pass filtered at 10 kHz and digitized at 20 kHz.

Traction force microscopy of 2D co-culture CM

Traction force microscopy (TFM) was executed by the construction and micropatterning of polyacrylamide gel constructs as previously described(Garay et al., 2022; Hald et al., 2016; Rothermel et al., 2022; Win et al., 2017). Briefly, stamps were made from PDMS from a master silicon wafer that was fabricated using photolithography. The stamps are rectangular arrays containing ~20 µm x 140 µm features used to micropattern islands that can be coated with protein for cell adhesion. For sterilization, the stamps were sonicated with 70% ethanol for 30 minutes then coated with ~123 µg of Cultrex-RGF per substrate diluted in DMEM/F12 with

HEPES and left at 37°C overnight. Next, 15 mm glass coverslips were rinsed with acetone and ethanol and then plasma treated for proper cleaning. The Cultrex-RGF stamps were then placed feature side down on the plasma-treated coverslips. The polyacrylamide gel was created and bound to a 25 mm glass coverslip as follows. The 25 mm glass coverslip was cleaned and treated with UV for 8 minutes. The glass was functionalized with 3% silane solution for 5 minutes. The prepolymer polyacrylamide gel solution was composed of 10/0.13% w/v acrylamide/bisacrylamide that was doped with 2% v/v 0.2 μm diameter red fluorescent microspheres. After the solution was degassed, 0.002/0.05% w/v initiators tetramethylethylenediamine/ammonium persulfate, 0.005% w/v N-hydroxysuccinimide ester, and 0.014% 1 M HCl were added to the gel. Then 10 μL of gel solution was pipetted onto the center of the silane-treated glass coverslip. The Cultrex-RGF coated coverslips were placed pattern side down onto the polyacrylamide gel solution and polymerized at room temperature for 1 hour. The polyacrylamide gel was rehydrated and the Cultrex-RGF coverslip was removed. The Young's modulus of the polymerized polyacrylamide gel was previously determined to be 13.5 kPa(Hald et al., 2016). At day 35 of the 2D direct co-culture experiments the co-cultures were singularized using 0.25% trypsin-EDTA as previously described and plated at a density of 35,000 cells per micropatterned polyacrylamide gel substrate into LASR medium containing 10 μM Rock Inhibitor. The following day the cells were given fresh LASR media. After 48 hours of recovery on the substrates, TFM measurements were acquired. Videos were taken on an inverted microscope in a CO_2 controlled chamber at 37°C. CM beat rate was controlled using 1 Hz electrical stimulation with a 40 ms bipolar pulse with a MyoPacer external field stimulator (IonOptix). Only micropatterned hiPSC-CM that were paced were imaged for these studies. Both brightfield and fluorescent images were acquired of the micropatterned hiPSC-CM at a frame rate of 30-100 frames per second. The images were taken for 3 seconds so that three consecutive contractions could be imaged in each video. Following image acquisition, the cells were lysed using sodium dodecyl sulfate, and cell free images of the fluorescent bead layer were acquired. The TFM measurements were analyzed in ImageJ as previously described(Garay et al., 2022; Hald et al., 2016; Rothermel et al., 2022; Win et al., 2017). The cell-induced bead displacement between the cell-attached and cell-free images at each time point was calculated using a particle image velocimetry algorithm in ImageJ. To generate a field of substrate traction stress vectors, an unconstrained Fourier-transform traction cytometry algorithm(Butler et al., 2002) was applied

to the field of cell-induced bead displacements with a regularization factor of $1\text{x}10^{-9}$ and a Poisson's ratio of 0.5. Substrate traction stress vectors acting on point n are defined by $\boldsymbol{T^n} = T_x^n \boldsymbol{e_x} + T_y^n \boldsymbol{e_y}$, where T_i^n is the traction stress acting in the ith direction ($i = x, y$), and $\boldsymbol{e_i}$ is the unit vector in the ith direction. Substrate traction forces acting at point n were taken as the substrate traction stress vector $\boldsymbol{T^n}$ multiplied by the area a^n over which $\boldsymbol{T^n}$ acts. At the cell-gel interface, substrate traction forces are balanced by internal cell forces $\boldsymbol{f^n}$ such that $\boldsymbol{f^n} + \boldsymbol{T^n} a^n = 0$. Substrate traction forces in turn are defined as $\boldsymbol{f^n} = f_x^n \boldsymbol{e_x} + f_y^n \boldsymbol{e_y}$, where f_i^n is the internal cell force acting on point n in the ith direction. The total internal cell force f_i acting in direction i was summed around the midline of the cell, such that $2f_i = \sum_n {f_i^n r_i^n}/{|r_i^n|}$, where r_i^n is the distance in the ith direction between the cell midline and the location n of the traction force. The force of contraction was defined as the difference in internal cell force between the cell at its maximum contraction and the uncontracted state. The contraction force was calculated for three consecutive contractions of the cell and then averaged.

Optical Mapping of EHTs

The EHT device underwent a 20-minute immersion in a 10 µM voltage-sensitive dye di-4-ANEPPS solution prepared in TSS. Subsequently, the dye mixture was replaced with fresh TSS. After a stabilization period of 2-5 minutes, EHT were excited using two continuous-excitation green lasers (532 nm, 1 W; Shanghai Dream Lasers Technology, Shanghai, China). Fluorescence intensity was captured for 10 seconds using high-resolution cameras (14-bit, 80 × 80-pixel, Little Joe, RedShirt Imaging, SciMeasure, Decatur, GA) at a rate of 500 frames per second(Garay et al., 2022; Hald et al., 2016; Rothermel et al., 2022; Win et al., 2017). Pacing at 1 Hz was conducted through a bipolar electrode, and optical mapping recordings were taken during this pacing and filtering using a bandpass filter 3-30 Hz. The duration of the optical APs (APD) was assessed at 80% repolarization. From this 2D APD maps were generated to illustrate the spatial dispersion in APD. We also quantified activation times (AT) at the peak of the derivative of voltage over time (dV/dt max), and calculated local conduction velocity (CV) as described previously(Kupfer et al., 2020; Lin et al., 2022).

Twitch force and stress measurements of EHTs

After 29-31 days in culture the EHTs were taken off the PDMS posts and mounted at their initial length on needles on the Mach-1 Micromechanical Tissue Tester (Biomomentum) using a 10g force transducer. The EHTs were paced using a platinum electrode at 1 Hz with a voltage of 7-9 V and pulse duration of 0.02s. The force measurements were acquired for 20s at strains of 0, 5, 10, 15, and 20%. Using MATLAB a low pass frequency filter was used to remove noise from force data and the average twitch force was calculated. The maximum force for each EHT at any given strain was reported as the maximum twitch force. Cryosections of the EHTs were used to determine the average cross-sectional area and the twitch stress was calculated by dividing the average twitch force by the average cross-sectional area of the cryosections (**Figure S8A-B**).

Murine E10, E12, and E17 heart harvesting, sectioning, and staining

All mouse experiments were performed using the outbred CD-1 mouse line. Upon mating, the detection of a mucus plug was considered day 1 of embryonic development. At the indicated time points, pregnant dams were euthanized, and embryos were extracted from the uterus. For the embryonic day (E) 10 and E12 whole embryos were used, while for E17 samples the heart was first excised from the embryo. For E17, hearts were immediately embedded in Tissue-Tek O.C.T. For E10 and E12 samples, embryos were fixed in fresh 4% paraformaldehyde (PFA) in PBS for 1-1.5 hours in a 12-well plate at room temperature, followed by a 3x15min wash in PBS. Afterward, embryos were placed in a 5% sucrose in PBS solution at 4°C until the tissue sunk to the bottom of the plate, followed by 10%, 15%, and finally 20% sucrose solution. E10 and E12 embryos were embedded in a 7.5% gelatin in a sucrose solution. Tissue blocks were sectioned into 4μm slices. E17 samples were fixed in 4% PFA solution for 10 minutes at room temperature, followed by two washes for 5 minutes in PBS and then directed to permeabilization. For E10 and E12 samples, prewarmed PBS was used to wash off gelatin. Slides were permeabilized in 2% BSA, 0.5% Tween-20 in PBS for 10-15 minutes and blocked in 2% BSA, 0.1% Tween-20 for 1 hour both at room temperature. Slides were incubated overnight at 4°C in a blocking solution with primary antibodies of nkx2.5, alpha-actinin, Ki67, and Wheat Germ Agglutinin (W11261, Invitrogen, 1:200 dilution). Antibody catalog number and dilution in **Table**

S2. After four times washing for 2 minutes in PBS, slides were incubated in secondary antibodies at room temperature at 1:400 dilution and DAPI for 1 hour. The images were taken with a Zeiss upright microscope equipped with an AxioCam MRm camera, using the 40x and 20x objectives, and processed using Fiji software. CM size was quantified using Fiji by tracing the area of individual CM. Around 120 CM sizes were measured for each time point. The percentage of proliferative ventricular CM was quantified using Cellprofiler. Sarcomere lengths were measured using the Fiji plugin SarcOptiM. At least 100 CM sarcomeres were measured for E12 and E17 time points, and 90 were measured for E10 samples.

Flow cytometry for CM proliferation, phenotype, and differentiation quality control

For proliferation assessment, the co-cultures were treated with EdU for 24 hours before harvesting on day 35. For co-culture proliferation and ventricular phenotype assessment, the co-cultures were singularized using 0.25% Trypsin-EDTA for 20-minutes at 37°C. Quality control samples of day 26 CM, EPD-FBs, and aLVCF were all singularized using the methods describes above. Once singularized, the cells were spun down at 200g for 5 minutes, the supernatant was poured off and the cells were resuspended in 1% PFA in PBS for 20 minutes. After 20 minutes the cells were spun down at 200g for 5 minutes, the fixative was removed, and the cells were resuspended in -20°C 90% v/v Methanol. The samples were stored in the freezer at -20°C until staining for flow cytometry. For staining, the cells were strained with a 70 µm cell strainer (Corning 352350) to remove any large clumps. ~300,000-1,000,000 cells per sample were separated into individual conical tubes and the methanol was washed off the cells by adding 2 mL of flow buffer (PBS with 5% BSA and 0.2% Triton-X-100) spinning down the cells at 200g for 5 minutes, pouring off the supernatant, then repeating this process once more. After the second wash, a P200 micropipette was used to remove as much residual flow buffer from the pellet as possible, and then the cells were either resuspended in 100 µL of Click-It EdU reaction mixture with Cy5-Azide for 30 minutes at room temperature (for proliferation assay only) or 100 µL of primary antibody dilution for 1hr at room temperature. For the proliferation assay, after the Click-It reaction, the cells were washed twice with 2mL of flow buffer as described above and then resuspended in 100 µL of primary antibody dilution for 1hr at room temperature. Following the primary antibody incubation, the samples were washed twice with flow buffer as described

above and then resuspended in 100 μL of secondary antibody solution. The cells were incubated at room temperature for 30 minutes and then washed twice with flow buffer. Following staining the cells were resuspended in ~300 μL of flow buffer and data was acquired for 10,000 events within the defined cell population using a BD Accuri C6 flow cytometer. The antibody dilutions and catalog number used for flow cytometry can be found in **Table S2**.

CM morphology assessment

At the endpoint of co-cultures, day 35, the 2D co-cultured were using 0.25% Trypsin-EDTA for 20 minutes at 37°C and replated onto 17.36 μg/mL Cultrex-RGF coated glass chamber slides at a density of ~10,000 cells/cm^2. After 24 hours the cells were fixed in diastole using 4% paraformaldehyde in PBS with 90mM KCl, 4mM EDTA, and 4mM $MgCl_2$ for diastolic arrest of the CM. The chamber slides were stored in PBS with 0.1% sodium azide at 4°C until staining. To stain, samples were permeabilized using 0.2% Triton-X-100 for 1 hour, blocked in BGST (50g/L BSA, 10g/L glycine, 2% Goat Serum, 0.1% Triton-X-100) for 2 hours and then incubated with anti-αActinin antibody for 3 hours s at room temperature. The primary antibody was washed off and the cells were incubated with AF-488 Goat anti-mouse secondary antibody for 1 hour. The antibody dilutions and catalog number can be found in **Table S2**. The sample was washed with PBS and the chamber attachment was removed from the slide and mounted with DAPI DABCO mounting medium (90% Glycerol, 25mg/mL DABCO, 2.5ng/mL DAPI in 1X PBS, pH 8.6) mounting medium and sealed with a coverslip. The slides were imaged using a 40X oil immersion lens on the FITC and DAPI channels. Five fields of view were imaged in each well, for each condition three wells were imaged. Data was collected in this fashion for three replicates for each of the M4C8, F7C6, and CCND2-hiPSC lines. The collected data was de-identified and given to a researcher for blinded analysis of CM a) sarcomere length using SarcOptiM ImageJ plugin(Pasqualin et al., 2016) b) cell perimeter c) cell area d) aspect ratio e) nucleation. The aspect ratio was defined as the longest axis of the cell that intersects the nucleus (the long axis) divided by the longest distance that is orthogonal to the long axis and intersects the nucleus (the short axis).

Immunocytochemistry and immunohistochemistry of direct co-cultures and EHTs

2D co-cultures seeded in 48-well plates and EHTs were preserved in 4% paraformaldehyde in PBS with 90mM KCl, 4mM EDTA, and 4mM $MgCl_2$ for diastolic arrest of the CM(Yücel et al., 2020). Briefly, the cells or EHTs were washed with DPBS without calcium or magnesium and then incubated for either 15 minutes (2D) or 45 minutes (EHTs) at room temperature followed by washing with PBS twice before storing in PBS with 0.1% sodium azide at 4°C until staining (2D) or cryo-embedding (EHTs). To process EHTs for cryo-embedding the fixed tissue was dehydrated in 30% sucrose for 48 hours. After dehydration the EHTs were cut in half and transferred into 50:50 30% Sucrose:OCT for 30 minutes then transferred into cryo-molds containing OCT and frozen in the -80°C until cryo-sectioning. Using a cryostat (Leica CM1900) the EHTs were sectioned across the center to determine the cross-sectional area as well as transversely at a 5-10 μm thickness. For staining, both the 2D wells and EHT sections were permeabilized using 0.2% Triton-X-100 for 1 hour and blocked in BGST for 3 hours before an overnight incubation at 4°C with the primary antibody dilutions in BGST. After the overnight incubation, the samples were washed twice with 0.2% Tween-20 and then twice with PBS. The samples were then incubated with secondary antibody dilution in BGST for 1.5 hours and then washed twice with 0.2% Tween-20 and with PBS. The samples were incubated with 1g/L DAPI in PBS for 20 minutes and washed with PBS 3 times. The EHT sections were mounted in Prolong™ Glass Antifade Mountant (Thermo P36982). The 2D wells were imaged at 20X magnification on a fluorescent microscope (Leica) and the EHT sections were imaged on an Olympus FluoView IX2 Inverted Confocal microscope with a 60X oil immersion lens. Phalloidin and DAPI images for tissue alignment as well as brightfield and phalloidin images for EHT cross-sectional area measurements were imaged at 20X magnification on a fluorescent microscope (Leica) using the ImageJ OrientationJ plugin (**Figure S8C-F**). The antibody dilutions and catalog number used for IHC can be found in **Table S2**.

Transmission electron microscopy of co-cultured CM

The 2D co-culture of conditions CM + EPC(+SB) and CM(+SB) were seeded as described above on 13 mm Thermonox® (Nalge Nunc International) plastic coverslips coated with 17.36 $\mu g/cm^2$ Cultrex-RGF and cultured for 9 days. The cells on the coverslips were fixed in 3%

paraformaldehyde and 1.5% glutaraldehyde in 0.1M sodium cacodylate buffer with 2.5% sucrose, 5mM calcium chloride, and 5mM magnesium chloride (pH 7.4) for 1-2 hr at room temperature (RT) after 8-days of co-culture. Cell cultures were rinsed in buffer (3x, 5 min ea), fixed in 1% OsO4 in 0.1M sodium cacodylate buffer for 1 hr at RT, rinsed in ultrapure water (NANOpure Infinity®; Barnstead/Thermo Fisher Scientific; Waltham, Maryland) (3x, 5 min ea), en bloc stained with 1% aqueous uranyl acetate for 1 hour at RT, and rinsed in ultrapure water (3x, 5 min ea). Samples were then dehydrated in an ethanol series (50%, 75%, 95% (all 2x), 100% (3x), 5 min ea) and infiltrated with Embed 812 resin (Electron Microscopy Sciences, Hatfield, Pennsylvania) (1:1 ethanol:resin, 2 hrs, 100% resin with catalyst, 2 hrs, 2x). Cultures were embedded by placing the coverslips upside-down on top of resin-filled capsule molds, then polymerized in a 60C oven for 48 hours. Ultrathin sections 80–100 nm thick were cut on a Leica Ultracut UCT microtome using a diamond knife and collected on formvar/carbon-coated copper slot (2mm x 1mm) grids (Electron Microscopy Sciences, Hatfield, Pennsylvania). They were stained with 3% aqueous uranyl acetate for 20 min, rinsed in ultrapure water (15 sec, 5x), stained with Sato's triple-lead stain (Sato 1968) for 3 min, and rinsed in ultrapure water (15 sec, 5x). Sections were examined with a JEOL JEM1400-Plus transmission electron microscope operating at 60 kV. Images were recorded with an Advanced Microscopy Techniques XR16 camera using AMT Capture Engine software ver. 7.0.0.187.

Single-cell RNA sequencing Sample Preparation

Single-cell RNA sequencing was done following the supplier's manual for the Chromium Next GEM Single Cell Sequencing Kits. The 2D co-cultures were singularized using 0.25% trypsin-EDTA for 20 minutes at 37°C as described above. The viability of the cells was determined via propidium iodide on the BD Accuri C6 flow analyzer and then the cells were prepped for fixed single-cell sequencing according to the Chromium Next GEM Single Cell Fixed RNA Sample Preparation Kit (10X Genomics 1000414). Libraries for single-cell sequencing were created using the Chromium Fixed RNA Kit, Human Transcriptome, 4 rxns x 4 BC (10X Genomics 1000475) by following the manual for probe hybridization and library creation. The samples were sequenced according to the 10X genomics FLEX single-cell sequencing workflow at the University of Minnesota Genomics Center (UMGC). Briefly, pooled libraries were denatured

and diluted to the appropriate clustering concentration. The libraries were then loaded onto the NovaSeq paired end flow cell and clustering occurred on board the instrument. Once clustering was completed, the sequencing reaction immediately began using the Illumina 2-color SBS chemistry. Upon completion of read 1, 2 separate 8 or 10 base pair index reads were performed. Finally, the clustered library fragments were re-synthesized in the reverse direction thus producing the template for paired end read 2.

Single-cell RNA sequencing analysis

Reads were aligned to the human reference genome provided by 10X Genomics (refdata-gex-GRCh38-2020-A) using cellRanger (v 7.1.0) and the Chromium_Human_Transcriptome_Probe_Set_v1.0_GRCh38-2020-A. Analysis was. Performed using R (v 4.1.0). Libraries were quality-controlled individually as follows: 1) The matrix of filtered counts per cell barcode was analyzed using Seurat (v 4.3.0.1). 2) Empty droplets were filtered out with a minimum total RNA and Feature counts per barcode based on their count distribution on a per-sample basis. 3) The presence of multiplets was assessed using the scDblFinder (v 1.8.0) package, followed by manual filtering of multiplets of the same cell type using an upper RNA count limit. 4) Gene expression was estimated on the clean data using the transformed counts (SCTransform) with the percent of mitochondrial reads as covariate.
To take full advantage of replication while avoiding confounding batch effects, biological replicates of the same condition were integrated into a single object using Seurat's standard workflow. Then, integrated treatment objects were merged into a single object and renormalized (SCTransform). This combined integration/modeling approach yielded cell type proportions that correlated with the expected experimental outcomes based on the seeded cell types, while the default integration or covariate batch modeling only did not. Principal component (PC) analysis of the combined object was performed for dimensionality reduction. UMAP reductions were used only for visualization purposes. Cell classification was performed using the FindNeighbors and FindClusters functions (Seurat) based on the first 25 PCs and the normalized counts. Cell cluster identification was performed based on their relative expression of genes of interest for the CM and EPC populations, their marker genes, and cluster abundance per treatment. Differential gene expression tests were done with the default parameters (test.use = "wilcox").

Overrepresentation Analysis (ORA) was performed on all sets of differentially expressed genes (DEGs) using the clusterProfiler (v 4.2.2) package and the gene ontology and KEGG databases in the msigdbr (v 7.5.1) package. Term and gene set databases were filtered to contain only genes detected across all treatments in the experiment (14263 genes). In detail, DEGs for each identity class were separated into up or down-regulated lists, the maximum between all or the top 300 of each list, ranked by the average log2 fold change, was tested with the enricher function using p-value and q-value cutoffs of 0.05 and the Benjamini–Hochberg correction for multiple hypothesis testing. Each of the three subcategories of the GO terms (BP, MF, and CC) and the KEGG database were tested separately.

qPCR for maturation markers

The EHTs from different groups were lysed using 600ul of RLT-lysis buffer containing β-mercaptoethanol (RNeasy kit (Qiagen)). Subsequently, the lysate was further homogenized using 21G and 25G needles (~10 times each) for complete cell lysis. Total RNA was isolated using the RNeasy kit according to the manufacturer's protocol, followed by on-column DNA digestion using DNase1 to remove any traces of DNA as per the instructions. The elution was performed using pre-heated (80C) DNase/RNase-free water. To obtain a higher yield, we perform a second round of elution using the eluate. The quantity and quality were assessed by measuring absorbance at 260nm and 280nm using a microplate reader instrument. For cDNA synthesis, 250 ng of total RNA was used and synthesized using the SuperScript IV VILO kit (Thermo Fisher Scientific) according to the manufacturer's protocol in a 20ul reaction volume. The reaction mix was diluted 10 times using DNase/RNase-free water for the next steps. Quantitative PCR (qPCR) was performed using gene-specific oligos and the SYBR-green method. The list of oligos used is provided in **Table S3**. For the qPCR analysis, each transcript was normalized to the housekeeping gene, *EDF1*. To visualize CM-specific changes, we further normalized the transcript levels to the levels of *TNNT2*.

Supplemental References

Butler, J. P., Tolić-Nørrelykke, I. M., Fabry, B., & Fredberg, J. J. (2002). Traction fields, moments, and strain energy that cells exert on their surroundings. *American Journal of Physiology-Cell Physiology*, *282*(3), C595–C605. https://doi.org/10.1152/ajpcell.00270.2001

Garay, B. I., Givens, S., Abreu, P., Liu, M., Yücel, D., Baik, J., Stanis, N., Rothermel, T. M., Magli, A., Abrahante, J. E., Goloviznina, N. A., Soliman, H. A. N., Dhoke, N. R., Kyba, M., Alford, P. W., Dudley, S. C., Van Berlo, J. H., Ogle, B., & Perlingeiro, R. R. C. (2022). Dual inhibition of MAPK and PI3K/AKT pathways enhances maturation of human iPSC-derived cardiomyocytes. *Stem Cell Reports*, *17*(9), 2005–2022. https://doi.org/10.1016/j.stemcr.2022.07.003

Hald, E. S., Timm, C. D., & Alford, P. W. (2016). Amyloid Beta Influences Vascular Smooth Muscle Contractility and Mechanoadaptation. *Journal of Biomechanical Engineering*, *138*(11), 111007. https://doi.org/10.1115/1.4034560

Kupfer, M. E., Lin, W.-H., Ravikumar, V., Qiu, K., Wang, L., Gao, L., Bhuiyan, D. B., Lenz, M., Ai, J., Mahutga, R. R., Townsend, D., Zhang, J., McAlpine, M. C., Tolkacheva, E. G., & Ogle, B. M. (2020). In Situ Expansion, Differentiation, and Electromechanical Coupling of Human Cardiac Muscle in a 3D Bioprinted, Chambered Organoid. *Circulation Research*, *127*(2), 207–224. https://doi.org/10.1161/CIRCRESAHA.119.316155

Lin, W.-H., Zhu, Z., Ravikumar, V., Sharma, V., Tolkacheva, E. G., McAlpine, M. C., & Ogle, B. M. (2022). A Bionic Testbed for Cardiac Ablation Tools. *International Journal of Molecular Sciences*, *23*(22), 14444. https://doi.org/10.3390/ijms232214444

Pasqualin, C., Gannier, F., Yu, A., Malécot, C. O., Bredeloux, P., & Maupoil, V. (2016). SarcOptiM for ImageJ: High-frequency online sarcomere length computing on stimulated cardiomyocytes. *American Journal of Physiology-Cell Physiology*, *311*(2), C277–C283. https://doi.org/10.1152/ajpcell.00094.2016

Rothermel, T. M., Cook, B. L., & Alford, P. W. (2022). Cellular Microbiaxial Stretching Assay for Measurement and Characterization of the Anisotropic Mechanical Properties of Micropatterned Cells. *Current Protocols*, *2*(2), e370. https://doi.org/10.1002/cpz1.370

Win, Z., Buksa, J. M., Steucke, K. E., Gant Luxton, G. W., Barocas, V. H., & Alford, P. W. (2017). Cellular Microbiaxial Stretching to Measure a Single-Cell Strain Energy Density Function. *Journal of Biomechanical Engineering*, *139*(7), 071006. https://doi.org/10.1115/1.4036440

Xie, A., Liu, H., Kang, G.-J., Feng, F., & Dudley, S. C. (2022). Reduced sarcoplasmic reticulum Ca2+ pump activity is antiarrhythmic in ischemic cardiomyopathy. *Heart Rhythm*, *19*(12), 2107–2114. https://doi.org/10.1016/j.hrthm.2022.08.022

Yücel, D., Solinsky, J., & Van Berlo, J. H. (2020). Isolation of Cardiomyocytes from Fixed Hearts for Immunocytochemistry and Ploidy Analysis. *Journal of Visualized Experiments*, *164*, 60938. https://doi.org/10.3791/60938

Zhu, W., Zhao, M., Mattapally, S., Chen, S., & Zhang, J. (2018). CCND2 Overexpression Enhances the Regenerative Potency of Human Induced Pluripotent Stem Cell–Derived Cardiomyocytes: Remuscularization of Injured Ventricle. *Circulation Research*, *122*(1), 88–96. https://doi.org/10.1161/CIRCRESAHA.117.311504
